# Supplementary material for: Single-pot mechanochemically-enabled fluorine atom closed-loop economy using PFASs as fluorinating agents
Source: Nat Commun. 2026 Mar 20;17:2696. doi: 10.1038/s41467-026-70766-9 (PMC13009461; doi:10.1038/s41467-026-70766-9)
Supplement: Supplementary file 1 — Supplementary Information [file 41467_2026_70766_MOESM1_ESM.pdf]

Supplementary Information

Single-Pot Mechanochemically-Enabled Fluorine Atom Closed-Loop Economy Using PFASs as Fluorinating Agents

Hao Long, Georgina Kirby, Lutz Ackermann*

Email:* ***Lutz.Ackermann@chemie.uni-goettingen.de*

Table of Contents

1 General Information 3

2 Optimization of the Reaction Conditions 4

3 Substrate Scope 7

3.1 Substrate scope of the fluorination 7

3.2 Setup for fluorination 8

3.3 Scale up of fluorination 8

4 Investigations into the degradation 10

4.1 Effect of milling time on fluoride ion concentration 10

4.2 MCD of PVDF using tBuOK from different brands 11

4.3 Procedure for IR analysis 12

4.4 Comparison of fluoride species from MCD PVDF/PTFE with standard KF 13

4.5 Complete MCD of PVDF 15

4.6 ^{13}C NMR analysis of polymer fragments generated by ball milling 16

5 Characterization Data 17

6 NMR Spectra 38

7 References 79

1 General Information

Materials: Starting materials and catalysts were purchased from Sigma-Aldrich, Alfa Aesar, BLD pharm and TCI Deutschland. When commercially available, substrates and solvents were used without further purification.

General data: If not otherwise noted, yields refer to isolated compounds, estimated to be >95% pure by NMR. NMR spectra were recorded on Bruker-300 MHz spectrometer or Bruker-400 MHz spectrometer. Chemical shifts (δ) are given in ppm relative to TMS. The residual solvent signals were used as references and the chemical shifts converted to the TMS scale ($CDCl_3$: δH = 7.27 ppm, δC = 77.2 ppm). Quantitative ^{19}F NMR spectra were recorded using a 30 second delay, using 4-F-anisole in $CDCl_3$ or hexafluoro-2-propanol (HFIP) in D_2O as an internal standard. All ^{19}F NMR yields were corrected based on response factors.

Infrared spectroscopy (IR): spectra were recorded on a Bruker FT-IR Alpha device. High resolution mass spectra were recorded on a Thermo LTQ Orbitrap XL (ESI+) or (ESI−).

^{19}F NMR yield using internal standard for fluoride yield:

$$Moles\ of\ product = \left(\frac{Integral\ of\ Product}{\left(\frac{Integral\ of\ IS}{number\ of\ fluorines\ in\ IS} \right)} \right) \times Moles\ of\ IS\ added$$

$$Yield\ of\ fluorine = \frac{\left(\frac{moles\ of\ product}{limiting\ reagent\ moles} \times 100 \right)}{number\ of\ fluorines\ in\ PFAS}$$

Mechanochemical reactions: Ball milling was conducted on a Retsch MM 400 mixer mill. Unless otherwise stated, mechanochemical reactions were carried out in either 5 mL Retsch mixer mill stainless-steel jar with stainless-steel ball (7 mm) or Retsch ZrO_2 30 mL jar with ZrO_2 15 mm ball. No precaution was taken to exclude air or water.

***Note**: PFAS-containing waste materials were disposed of in full compliance with standard laboratory hazardous waste protocols which is in full compliance with German and EU waste safety regulations.*

2 Optimization of the Reaction Conditions

Optimization of reaction times

To a 5 mL stainless-steel milling jar equipped with a PTFE sealing ring was added a 7 mm stainless-steel ball, PVDF (1.5 equiv., 0.3 mmol), and K_3PO_4 (1.5 equiv., 0.3 mmol). The jar was sealed tightly, mounted on the shaker mill, and subjected to milling at 30 Hz for 100 minutes (B1). After completion, the jar was opened, and sulfonyl chloride **1** (1.0 equiv., 0.2 mmol) was added to the resulting black mixture. The jar was resealed and subjected to further milling at 30 Hz for X minutes (20–100 min) (B2). Upon completion of B2, 4-fluoroanisole and $CDCl_3$ (0.6 mL) were added to the jar. The jar was sealed and shaken for 20 seconds. The resulting mixture was filtered through a small pad of celite® directly into an NMR tube. Yields were determined by quantitative ^{19}F NMR, and all values were corrected using appropriate response factors.

Note: *Although there are no reported health risks to the mechano-degradation of PFASs it is always imperative to take careful precautions. The ball mill was always opened inside a fume hood and the resulting powder mixture was always handled with gloves inside a fume hood.*

Table S1. Varying the time of B2[a]

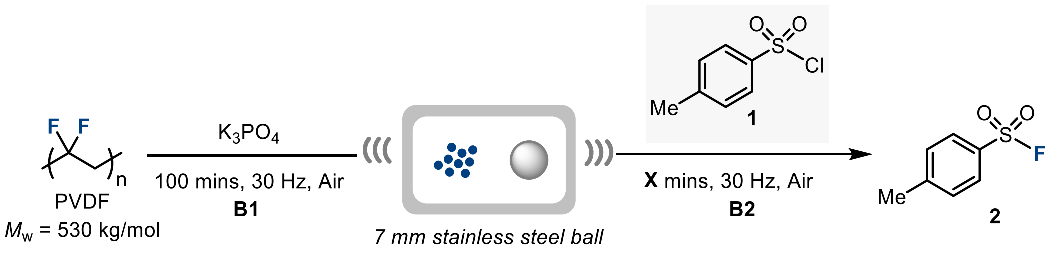

Entry	Time B1 (mins)	Time B2 (mins)	2 %[b]
**1**	100	20	17
**2**	100	40	28
**3**	100	60	41
**4**	100	80	39
**5**	100	100	49*

[a]Reaction conditions: Ball milling was conducted in a 5 mL 304 stainless-steel jar, using a stainless-steel ball with a diameter of 7 mm, PVDF (0.3 mmol, 1.5 equiv.), K_3PO_4 (0.3 mmol, 1.5 equiv.), **1** (0.2 mmol), 30 Hz. [b]Yield determined by ^{19}F NMR analysis is determined by using 4-F-anisole as the internal standard. *Isolated yield

Optimization of bases used

To a 5 mL stainless-steel milling jar equipped with a PTFE sealing ring was added a 7 mm stainless-steel ball, PVDF (1.5 equiv., 0.3 mmol), and bases (1.5 equiv., 0.3 mmol). The jar was sealed tightly, mounted on the shaker mill, and subjected to milling at 30 Hz for 100 minutes (B1). After completion, the jar was opened, and sulfonyl chloride **1** (1.0 equiv., 0.2 mmol) was added to the resulting black mixture. The jar was resealed and subjected to further milling at 30 Hz for 100 minutes (B2). Upon completion of B2, 4-fluoroanisole and $CDCl_3$ (0.6 mL) were added to the jar. The jar was sealed and shaken for 20 seconds. The resulting mixture was filtered through a small pad of celite® directly into an NMR tube. Yields were determined by quantitative ^{19}F NMR, and all values were corrected using appropriate response factors.

Table S2. Trial of different bases[a]

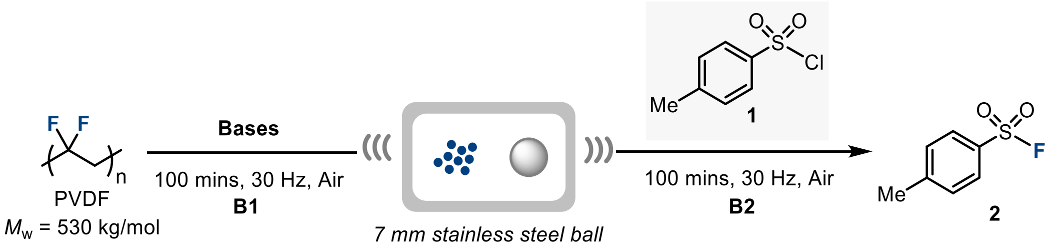

Entry	Base (1.5 equiv.)	2 %[b]
**1**	K_3PO_4	49
**2**	Pyridine	2
**3**	DMPU	-
**4**	DMAP	4
**5**	DBU	32
**6**	*t*BuOK	95*
**7**	Cs_2CO_3	56
**8**	$Ca(OH)_2$	-
**9**	KOAc	trace
**10**	*n*Bu_4NOAc	trace
**11**	MeONa	10
**12**	KOH (B1 = 60 mins)	10
**13**	K_2HPO_4 (B1 = 60 mins)	5

[a]Reaction conditions: Ball milling was conducted in a 5 mL 304 stainless-steel jar, using a stainless-steel ball with a diameter of 7 mm, PVDF (0.3 mmol, 1.5 equiv.), bases (0.3 mmol, 1.5 equiv.), **1** (0.2 mmol), 30 Hz. [b]Yield determined by ^{19}F NMR analysis using 4-F-anisole as the internal standard. *Isolated yield.

Optimization of reaction with PTFE

To a 30 mL ZrO_2 milling jar equipped with a PTFE sealing ring and ensuring no contact between the PTFE sealing ring and the inner surface of the jar, a 15 mm ZrO_2 ball, PTFE (1.5 equiv., 0.3 mmol), and *t*BuOK (1.5 equiv., 0.3 mmol) were added. The jar was sealed tightly, mounted on the shaker mill, and subjected to milling at 30 Hz for 200 minutes (B1). Upon completion, the jar was opened, and sulfonyl chloride **1** (1.0 equiv., 0.2 mmol) was added to the resulting black mixture. The jar was resealed and milled for an additional 100 minutes at 30 Hz (B2). After B2, 4-fluoroanisole and $CDCl_3$ (0.6 mL) were added to the jar, which was then sealed and shaken for 20 seconds. The resulting mixture was filtered through a small pad of Celite® directly into an NMR tube. Yields were determined by quantitative ^{19}F NMR, with all values corrected using appropriate response factors.

Table S3. Further optimization of PTFE[a]

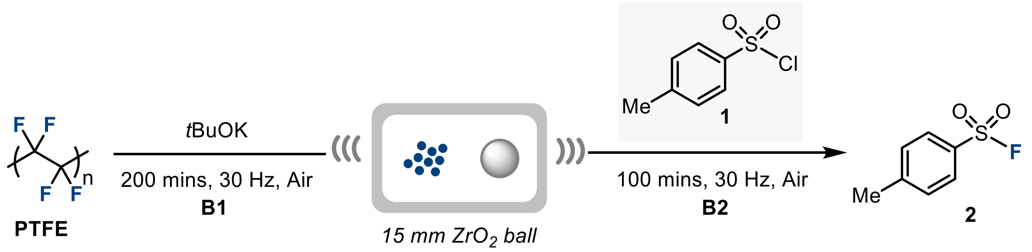

Entry	Base	Jar and ball	Time B1 (mins)	**2** %[b]
**1**	K_3PO_4	SUS jar and 7 mm SUS ball	100	0
**2**	K_3PO_4	SUS jar and 7 mm SUS ball	200	0
**3**	Cs_2CO_3	SUS jar and 7 mm SUS ball	100	0
**4**	KOH	SUS jar and 7 mm SUS ball	100	0
**5**	*t*BuOK	SUS jar and 7 mm SUS ball	100	0
**6**	KOH	ZrO_2 jar and 15 mm ZrO_2 ball	100	trace
**7**	K_3PO_4	ZrO_2 jar and 15 mm ZrO_2 ball	100	0
**8**	Cs_2CO_3	ZrO_2 jar and 15 mm ZrO_2 ball	100	0
**9**	*t*BuOK	ZrO_2 jar and 15 mm ZrO_2 ball	100	55
**10**	*t*BuOK	ZrO_2 jar and 15 mm ZrO_2 ball	200	88*
**11**	*t*BuOK	SUS jar and 2*10 mm SUS balls	300	75

[a]Reaction conditions: Ball milling was conducted in a 30 mL ZrO_2 jar, using a ZrO_2 ball with a diameter of 15 mm, PTFE (0.3 mmol, 1.5 equiv.), *t*BuOK (0.3 mmol, 1.5 equiv.), **1** (0.2 mmol), B1 = 200 mins, B2 = 100 mins, 30 Hz. [b]Yield determined by ^{19}F NMR analysis is determined by using 4-F-anisole as the internal standard. *Isolated yield

3 Substrate Scope

3.1 Substrate scope of the fluorination

General procedure A: To a 5 mL stainless-steel milling jar equipped with a PTFE sealing ring was added a 7 mm stainless-steel ball, PFASs (1.5 equiv., 0.3 mmol), and *t*BuOK (1.5 equiv., 0.3 mmol). The jar was tightly sealed, mounted on the shaker mill, and milled at 30 Hz for 100 minutes (B1). Upon completion of B1, the jar was opened, and sulfonyl chloride (1.0 equiv., 0.2 mmol) was added to the resulting black mixture. The jar was then resealed and subjected to a second milling cycle (B2) for an additional 100 minutes at 30 Hz. After completion of B2, 3 mL of $CHCl_3$ was added to wash the black solid. The mixture was filtered through a small pad of celite®, and the reaction vessel was subsequently rinsed with an additional 2 × 3 mL of $CHCl_3$, followed by a second filtration. The combined filtrates were concentrated under reduced pressure and dried under vacuum to afford the pure target product without the need for further purification.

General procedure B: To a 30 mL ZrO_2 milling jar equipped with a PTFE sealing ring and ensuring no direct contact between the PTFE sealing ring and the inner surface of the jar, a 15 mm ZrO_2 ball, PFASs (1.5 equiv., 0.3 mmol), and *t*BuOK (1.5 equiv., 0.3 mmol) were added. The jar was tightly sealed, mounted on the shaker mill, and milled at 30 Hz for 200 minutes (B1). Upon completion of B1, the jar was opened, and sulfonyl chloride **1** (1.0 equiv., 0.2 mmol) was added to the resulting black mixture. The jar was then resealed and subjected to a second milling cycle (B2) for an additional 100 minutes at 30 Hz. After completion of B2, 3 mL of $CHCl_3$ was added to wash the black solid. The mixture was filtered through a small pad of celite®, and the reaction vessel was subsequently rinsed with an additional 2 × 3 mL of $CHCl_3$, followed by a second filtration. The combined filtrates were concentrated under reduced pressure and dried under vacuum to afford the pure target product without further purification.

Note: *Although there are no reported health risks to the mechano-degradation of PFASs it is always imperative to take careful precautions. The ball mill was always opened inside a fume hood and the resulting powder mixture was always handled with gloves inside a fume hood.*

3.2 Setup for fluorination

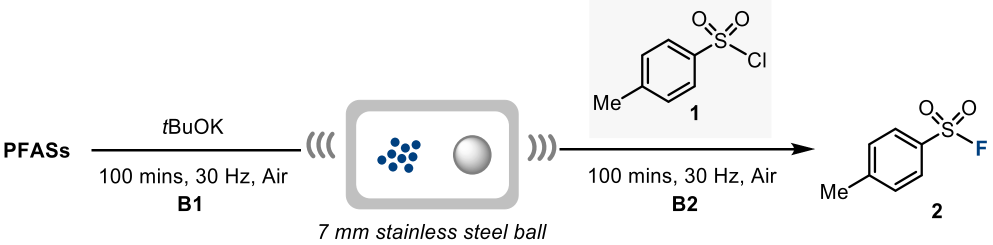

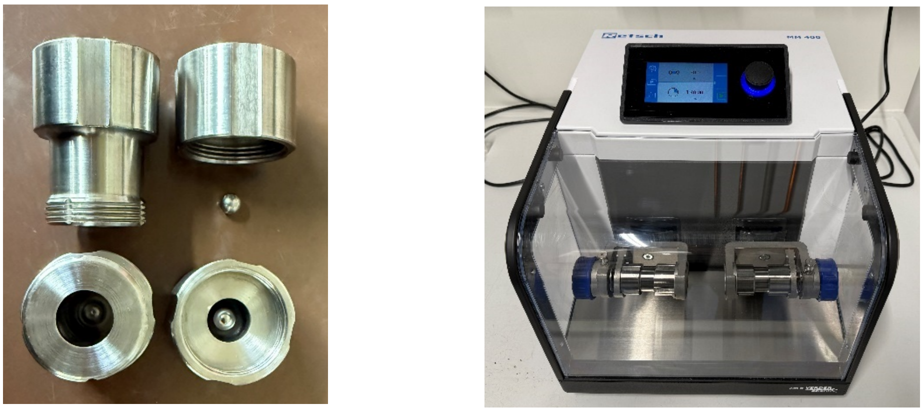

Supplementary Figure 1. Left: 5 mL stainless-steel milling jar and 7 mm stainless-steel ball. Right: Retsch Vibrating Mill MM 400.

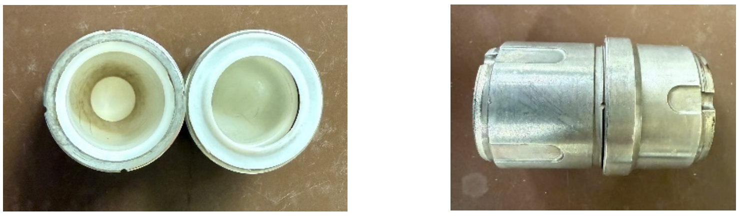

Supplementary Figure 2. Left: the inner surface of the ZrO_2 milling jar and 15 mm ZrO_2 ball. Right: 30 mL ZrO_2 milling jar.

3.3 Scale up of fluorination

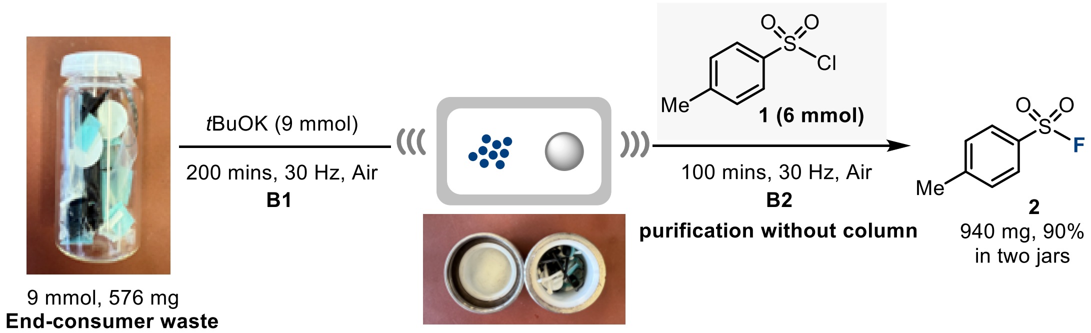

To two 30 mL ZrO_2 milling jars (each equipped with a PTFE sealing ring and ensuring no direct contact between the PTFE sealing ring and the inner surface of the jars), a 15 mm ZrO_2 ball was added separately. End-consumer waste (1.5 equiv., 9 mmol, 576 mg) and *t*BuOK (1.5 equiv., 9 mmol, 1.0 g) were evenly divided into two portions (4.5 mmol each) and added to the respective jars. The jars were tightly sealed, mounted on the shaker mill, and subjected to two

consecutive milling cycles of 100 minutes each at 30 Hz (B1, total 200 minutes). After completion of B1, the jars were opened and sulfonyl chloride **1** (1.0 equiv., 6 mmol) was added to the resulting black mixtures. The jars were resealed and subjected to a second milling cycle (B2) for an additional 100 minutes at 30 Hz. Upon completion of B2, 15 mL of $CHCl_3$ was added to each jar to wash the resulting black solid. The mixtures were filtered through a pad of celite®, and the reaction vessels were subsequently rinsed with an additional 2 × 15 mL of $CHCl_3$, followed by a second filtration. The combined filtrates were concentrated under reduced pressure and dried under vacuum to afford the pure target product. This gram-scale procedure delivered the desired product **2** as a yellow solid (940 mg, 90% yield) without the need for column chromatography, relying solely on filtration and solvent removal for isolation. The high yield and operational simplicity highlight the scalability and practicality of this mechanochemical protocol.

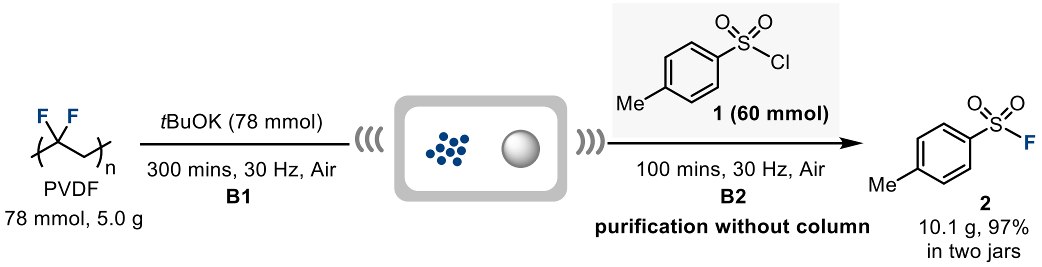

To two 30 mL ZrO_2 milling jars (each equipped with a PTFE sealing ring and ensuring no direct contact between the PTFE sealing ring and the inner surface of the jars), a 15 mm ZrO_2 ball was added separately. PVDF (1.3 equiv., 78 mmol) and *t*BuOK (1.3 equiv., 78 mmol) were evenly divided into two portions (39 mmol each) and added to the respective jars. The jars were tightly sealed, mounted on the shaker mill, and subjected to three consecutive milling cycles of 100 minutes each at 30 Hz (B1, total 300 minutes). After completion of B1, the jars were opened and sulfonyl chloride **1** (1.0 equiv., 60 mmol) was added to the resulting black mixtures. The jars were resealed and subjected to a second milling cycle (B2) for an additional 100 minutes at 30 Hz. Upon completion of B2, 30 mL of $CHCl_3$ was added to each jar to wash the resulting black solid. The mixtures were filtered through a pad of celite®, and the reaction vessels were subsequently rinsed with an additional 2 × 30 mL of $CHCl_3$, followed by a second filtration. The combined filtrates were concentrated under reduced pressure and dried under vacuum to afford the pure target product. This gram-scale procedure delivered the desired product **2** as a yellow solid (10.1 g, 97% yield) without the need for column chromatography, relying solely on filtration and solvent removal for isolation. The high yield and operational simplicity highlight the scalability and practicality of this mechanochemical protocol.

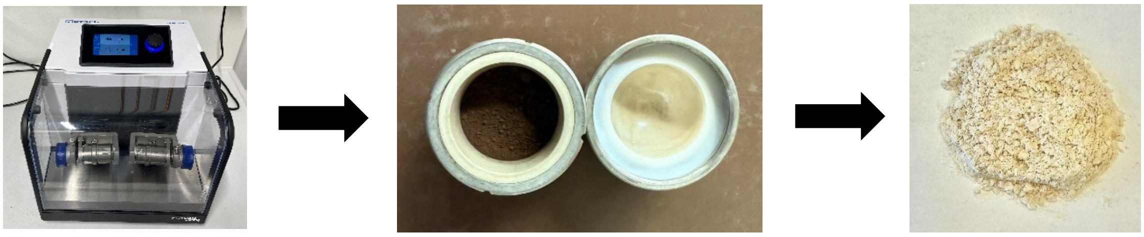

Supplementary Figure 3. Left: Scaled up with Retsch Vibrating Mill MM 400. Middle: black mixture inside the jars after ball milling. Right: 10.1 g pure target product without further purification.

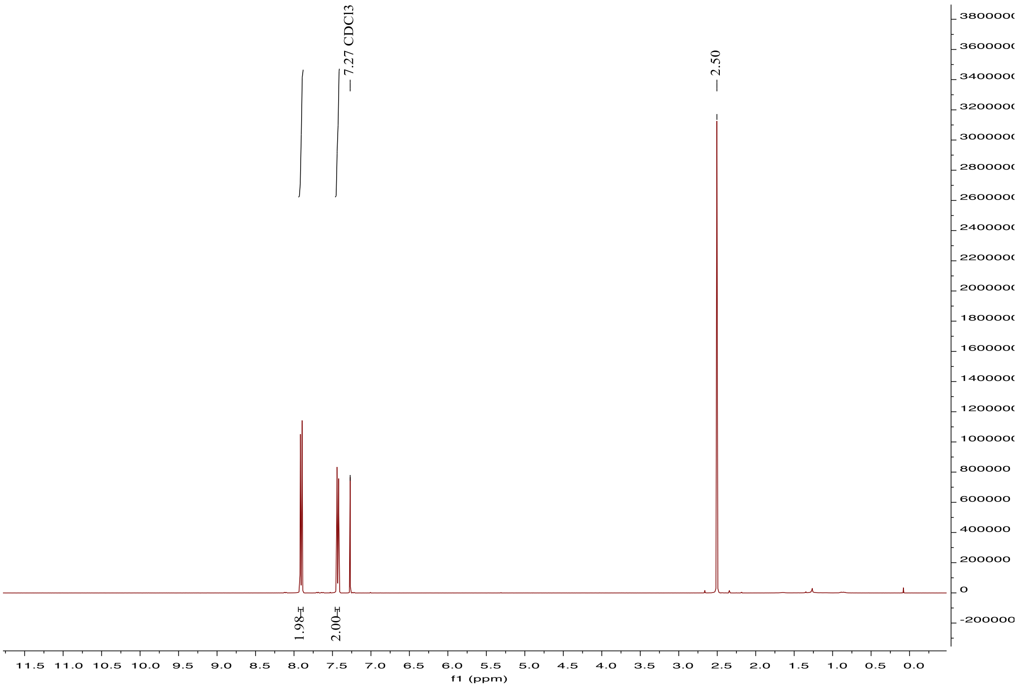

Supplementary Figure 4. ^{1}H-NMR (300 MHz, $CDCl_3$) spectrum of **2** in scale up reaction.

4 Investigations into the degradation

4.1 Effect of milling time on fluoride ion concentration

To a 5 mL stainless-steel milling jar (equipped with a PTFE sealing ring) was added a 7 mm stainless-steel ball, PVDF (0.3 mmol), and base (0.3 mmol). The jar was tightly sealed, mounted on the shaker mill, and milled at 30 Hz for **X** minutes. Upon completion, the jar was opened, and a mixture of HFIP and D_2O (0.6 mL) was added. The jar was then resealed and shaken for 20 seconds, after which the mixture was left to stand undisturbed for 2 hours to allow the undissolved polymer to settle. The aqueous layer (D_2O) was carefully pipetted off, filtered through cotton directly into an NMR tube, and analyzed by ^{19}F NMR spectroscopy. This procedure was used to quantify the release of fluoride ions from PVDF under basic mechanochemical conditions.

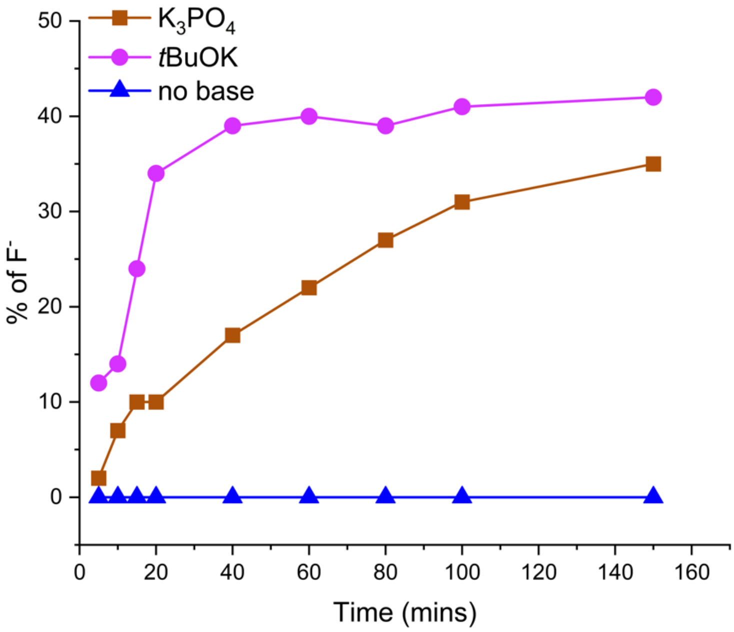

Supplementary Figure 5. Graph showing the percentage of fluoride produced from ball milling PVDF with K_3PO_4 and *t*BuOK. (Graph made in Origin from data using ^{19}F NMR)

Table S4. Generation of fluoride over time

	Ball milling time (minutes)								
	**5**	**10**	**15**	**20**	**40**	**60**	**80**	**100**	**150**
**K_3PO_4**	2 %	7 %	10 %	10 %	17 %	22 %	27 %	31 %	35 %
	0.01 mmol F^-	0.04 mmol F^-	0.06 mmol F^-	0.06 mmol F^-	0.10 mmol F^-	0.13 mmol F^-	0.16 mmol F^-	0.19 mmol F^-	0.21 mmol F^-
***t*BuOK**	12 %	14 %	24 %	34 %	39 %	40 %	39 %	41 %	42 %
	0.08 mmol F^-	0.09 mmol F-	0.14 mmol F^-	0.21 mmol F^-	0.23 mmol F^-	0.24 mmol F^-	0.23 mmol F^-	0.25 mmol F^-	0.25 mmol F^-
**No base**	0 %	0 %	0 %	0 %	0 %	0 %	0 %	0 %	0 %
	-	-	-	-	-	-	-	-	-

4.2 MCD of PVDF using *t*BuOK from different brands

To a 5 mL stainless-steel milling jar (equipped with a PTFE sealing ring) was added a 10 mm stainless-steel ball, PVDF (0.3 mmol), and *t*BuOK (0.3 mmol). The jar was tightly sealed, mounted on the shaker mill, and milled at 30 Hz for 100 minutes. Upon completion, the jar was opened, and a mixture of HFIP and D_2O (0.6 mL) was added. The jar was then resealed and shaken for 20 seconds, after which the mixture was left to stand undisturbed for 2 hours to allow the undissolved polymer to settle. The aqueous layer (D_2O) was carefully pipetted off, filtered through cotton directly into an NMR tube, and analyzed by ^{19}F NMR spectroscopy.

Table S5. Generation of fluoride using *t*BuOK from different brands

Entry	tBuOK from different manufacturers/batches	Fluoride yield(%)*
***t*BuOK-1**	97% pure, TCI, P1008	38%
***t*BuOK-2**	98% pure, Thermo Fisher, 168885000 (500g)	39%
***t*BuOK-3**	98% pure, Thermo Fisher, 168881000 (25g)	43%
***t*BuOK-4**	98% pure, Sigma-Aldrich, 156671	43%

Fluoride yield by ^{19}F NMR using HFIP as internal standard.

Table S6. ICP-MS data for the different brands

Sample	Mn (ppm)	Ni (ppm)	Cu (ppm)	Cr (ppm)	Fe (ppm)	Pd (ppm)	Ir (ppm)	Co (ppm)	Zn (ppm)
***t*BuOK -1**	0.21	0.34	4.82	2.07	13.6	0.006	0.004	0.06	2.26
***t*BuOK -2**	0.25	1.57	5.52	2.17	22.4	0.004	-	0.08	2.34
***t*BuOK -3**	0.22	1.82	4.09	1.97	17.8	0.007	-	0.06	2.39
***t*BuOK -4**	0.21	0.29	3.05	3.39	15.9	0.011	-	0.04	1.49
***t*BuOK -1** after Ball mill	4.58	0.33	16.6	20.3	129.3	0.489	-	0.58	48.2

- = intensity below calibration range

4.3 Procedure for IR analysis

To a 5 mL stainless-steel milling jar (equipped with a PTFE sealing ring) was added a 7 mm stainless-steel ball, PVDF (0.3 mmol), and *t*BuOK (0.3 mmol). The jar was tightly sealed, mounted on the shaker mill, and milled at 30 Hz for 100 minutes. Upon completion, methanol (3 × 5 mL) was added to the jar, and the resulting mixture was transferred to a centrifuge tube. The mixture was centrifuged for 10 minutes, after which the supernatant was carefully removed. The precipitated polymer was dried and subsequently analyzed by IR spectroscopy. The appearance of a new absorption band at 1650 cm^{-1} indicated the formation of C=C double bonds.

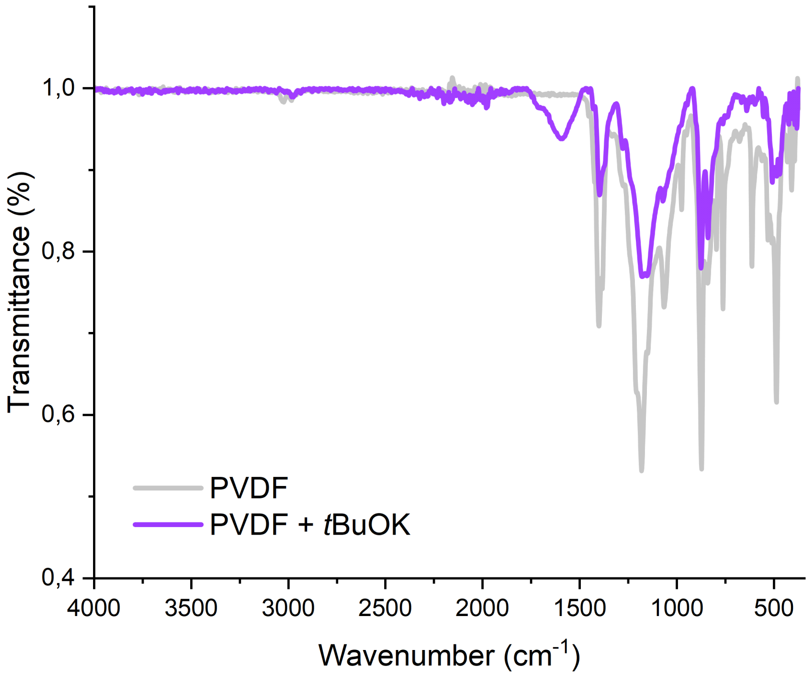

Supplementary Figure 6. Infrared spectrum of PVDF (grey) and PVDF after 100 minutes of ball milling with *t*BuOK.

4.4 Comparison of fluoride species from MCD PVDF/PTFE with standard KF

PVDF MCD procedure: To a 5 mL stainless-steel milling jar (equipped with a PTFE sealing ring) was added a 7 mm stainless-steel ball, PVDF (0.3 mmol), and *t*BuOK (0.3 mmol). The jar was tightly sealed, mounted on the shaker mill, and milled at 30 Hz for 100 minutes. Upon completion, the jar was opened, and a mixture of HFIP and D_2O (0.6 mL) was added. The jar was then resealed and shaken for 20 seconds, after which the mixture was left to stand undisturbed for 2 hours to allow the undissolved polymer to settle. The D_2O layer was carefully pipetted off, filtered through cotton into an NMR tube, and analyzed by ^{19}F NMR spectroscopy.

PTFE MCD procedure: To a 30 mL ZrO_2 milling jar (equipped with a PTFE sealing ring and ensuring no direct contact between the PTFE sealing ring and the inner surface of the jar) was added a 15 mm ZrO_2 ball, PTFE (0.3 mmol), and *t*BuOK (0.3 mmol). The jar was tightly sealed, mounted on the shaker mill, and milled at 30 Hz for 200 minutes. Upon completion, the jar was opened, and a mixture of HFIP and D_2O (0.6 mL) was added. The jar was then resealed and shaken for 20 seconds, after which the mixture was left to stand undisturbed for 2 hours to allow the undissolved polymer to settle. The D_2O layer was carefully pipetted off, filtered through cotton into an NMR tube, and analyzed by ^{19}F NMR spectroscopy.

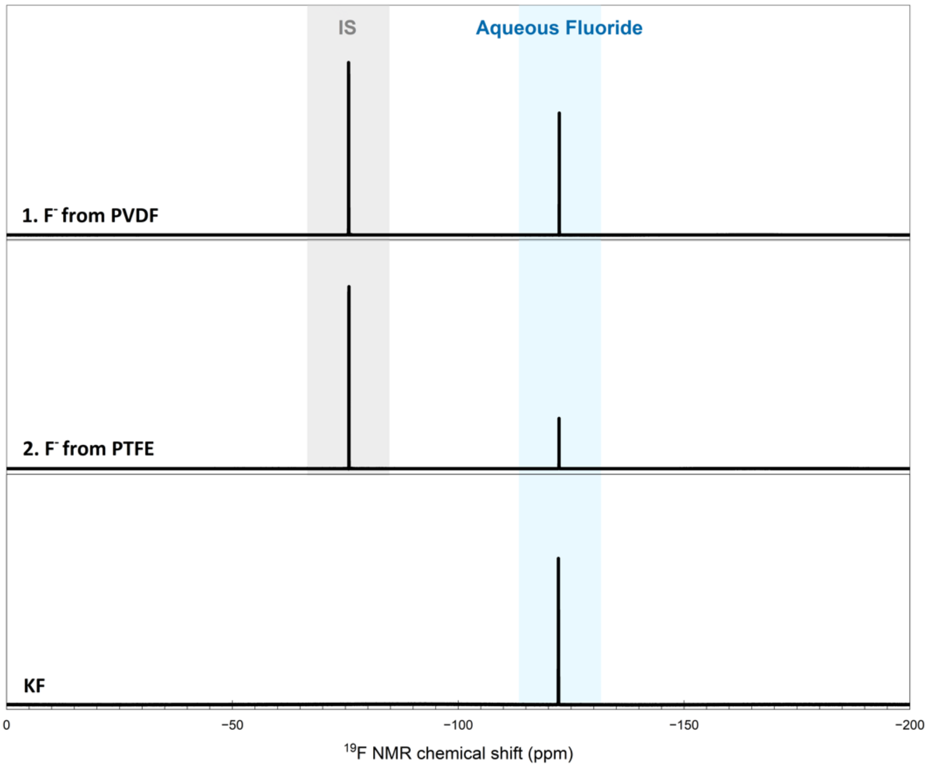

Supplementary Figure 7. ^{19}F NMR of the fluoride produced from ball milling PVDF and PTFE with *t*BuOK. ^{19}F NMR of KF as a comparison (bottom). Comparison graph generated using Origin.

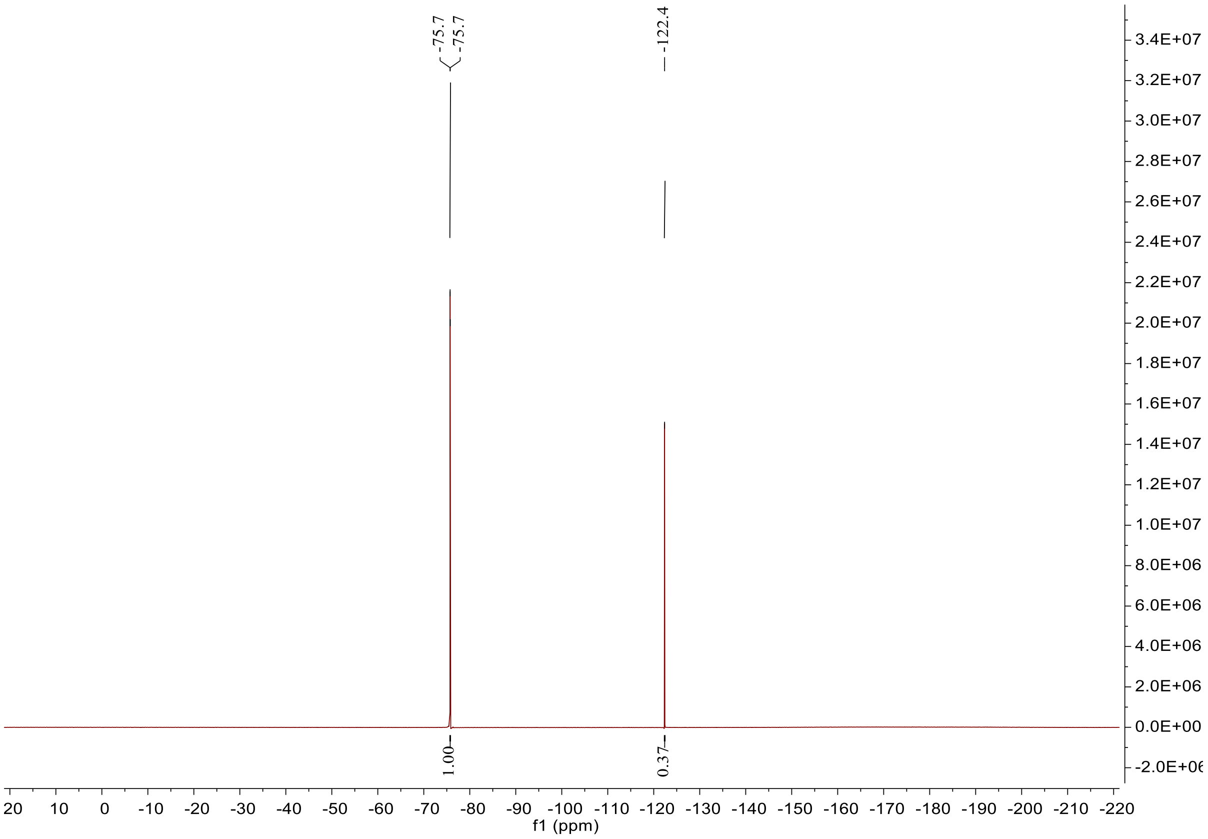

Supplementary Figure 8. ^{19}F-NMR of fluoride produced from ball milling with PVDF with *t*BuOK.

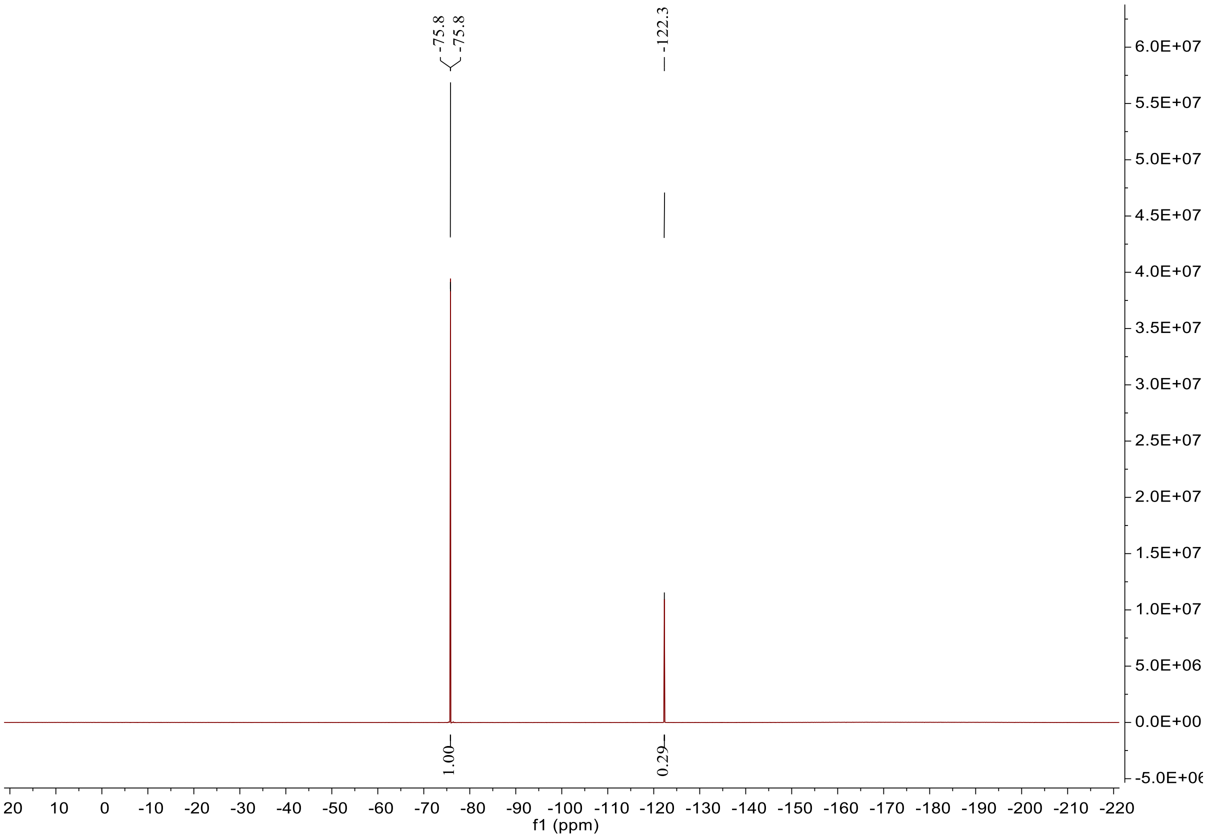

Supplementary Figure 9. ^{19}F-NMR of fluoride produced from ball milling with PTFE with *t*BuOK.

4.5 Complete MCD of PVDF

To a 5 mL stainless-steel milling jar (equipped with a PTFE sealing ring) was added a 10 mm stainless-steel ball, PVDF (0.3 mmol), and *t*BuOK (0.6 mmol). The jar was tightly sealed, mounted on the shaker mill, and milled at 30 Hz for 100 minutes. Upon completion, *t*BuOK (0.6 mmol) was added to the jar and the mixture was milled for another 100 minutes. Upon completion, the jar was opened and a mixture of HFIP and D_2O (0.6 mL) was added. The jar was then resealed and shaken for 20 seconds, after which the mixture was left to stand undisturbed for 2 hours to allow the undissolved polymer to settle. The D_2O layer was carefully pipetted off, filtered through cotton into an NMR tube, and analyzed by ^{19}F NMR spectroscopy.

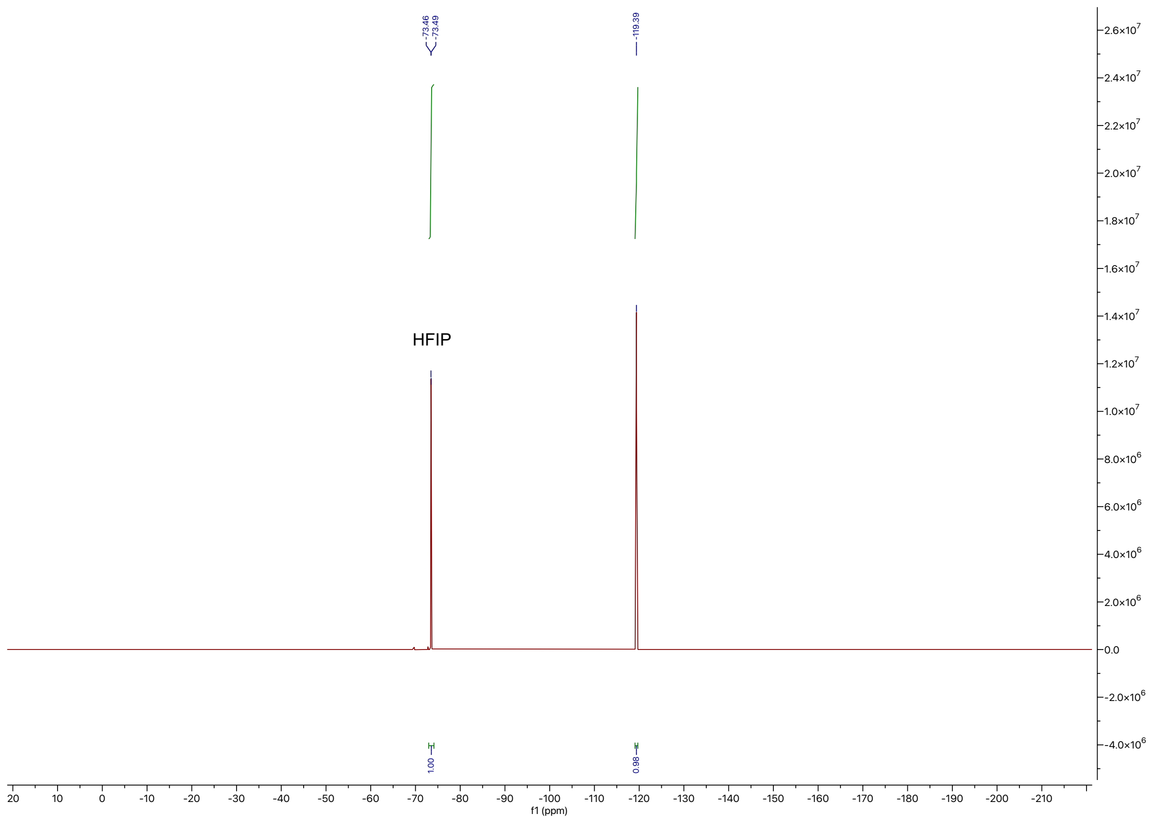

Supplementary Figure 10. ^{19}F-NMR of fluoride produced from ball milling with PVDF with *t*BuOK (complete defluorination).

4.6 ^{13}C NMR analysis of polymer fragments generated by ball milling

To a 30 mL ZrO_2 milling jar (equipped with a PTFE sealing ring and ensuring no direct contact between the PTFE sealing ring and the inner surface of the jar) was added a 15 mm ZrO_2 ball, perfluorooctane (0.3 mmol), and *t*BuOK (0.3 mmol). The jar was tightly sealed, mounted on the shaker mill, and milled at 30 Hz for 200 minutes. Upon completion, the jar was opened, and a mixture of HFIP and D_2O (0.6 mL) was added. The jar was then resealed and shaken for 20 seconds, after which the mixture was left to stand undisturbed for 2 hours to allow the undissolved polymer to settle. The D_2O layer was carefully pipetted off, filtered through cotton into an NMR tube, and analyzed by ^{13}C NMR spectroscopy.

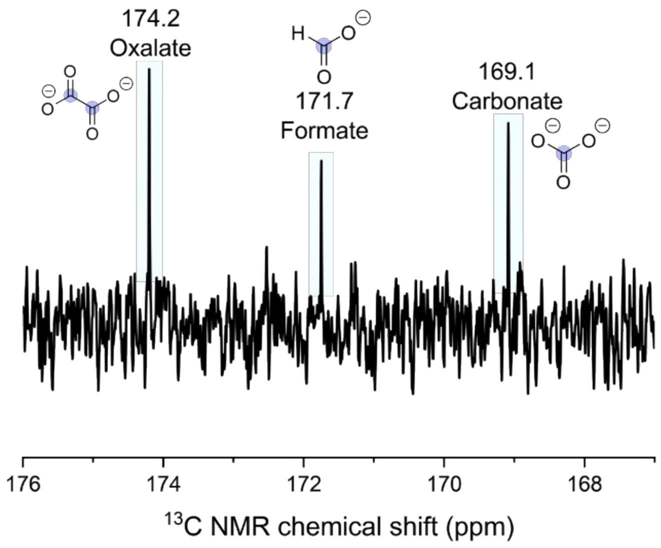

Supplementary Figure 11. ^{13}C-NMR (75 Hz, $CDCl_3$) analysis of polymer fragments generated by ball milling, Left: oxalate. Middle: formate. Right: carbonate.

5 Characterization Data

4-Methylbenzenesulfonyl fluoride (2)

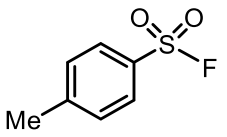

2

The **General Procedure A** was followed using tosyl chloride substrate (0.2 mmol, 38 mg) to afford **2** (33 mg, 95% yield) as a yellow solid. ^{1}H NMR (300 MHz, $CDCl_3$) δ 7.99 – 7.81 (m, 2H), 7.49 – 7.39 (m, 2H), 2.50 (s, 3H). ^{13}C NMR (75 MHz, $CDCl_3$) δ 147.3 (C_q), 130.4 (CH), 130.2 (d, $J_{C–F}$ = 24.3 Hz, C_q), 128.6 (CH), 22.0 (CH_3). ^{19}F NMR (282 MHz, $CDCl_3$) δ 66.2. **HR-MS** (ESI) m/z $C_7H_7O_3S^-$ $[M–F+O]^-$ calculated for 171.0121, found 171.0110. This compound was known.[1]

Benzenesulfonyl fluoride (3)

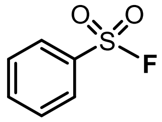

3

The **General Procedure A** was followed using benzene sulfonyl chloride substrate (0.2 mmol, 35 mg) to afford **3** (30 mg, 95% yield) as a yellow oil. Product is volatile. ^{1}H NMR (400 MHz, $CDCl_3$) δ 8.08 – 7.99 (m, 2H), 7.84 – 7.74 (m, 1H), 7.70 – 7.58 (m, 2H). ^{13}C NMR (126 MHz, $CDCl_3$) δ 135.7 (CH), 133.3 (d, $J_{C–F}$ = 24.3 Hz, C_q), 129.8 (CH), 128.6 (CH). ^{19}F NMR (471 MHz, $CDCl_3$) δ 65.9. **HR-MS** (ESI) m/z $C_6H_5O_3S^-$ $[M–F+O]^-$ calculated for 156.9965, found 156.9964. This compound was known.[1]

4-(*tert*-butyl)Benzenesulfonyl fluoride (4)

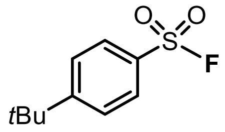

4

The **General Procedure A** was followed using 4-*tert*-butylbenzene sulfonyl chloride substrate (0.2 mmol, 43 mg) to afford **4** (42 mg, 98% yield) as a yellow solid. ^{1}H NMR (300 MHz, $CDCl_3$) δ 8.02 – 7.87 (m, 2H), 7.73 – 7.57 (m, 2H), 1.38 (s, 9H). ^{13}C NMR (75 MHz, $CDCl_3$) δ 160.1 (C_q), 130.2 (d, $J_{C–F}$ = 24.2 Hz, C_q), 128.5 (CH), 126.9 (CH), 35.7 (C_q), 31.1 (CH_3). ^{19}F NMR (282 MHz, $CDCl_3$) δ 66.1. **HR-MS** (ESI) m/z $C_{10}H_{13}O_3S^-$ $[M–F+O]^-$ calculated for 213.0591, found 213.0583. This compound was known.[1]

4-Methoxybenzenesulfonyl fluoride (5)

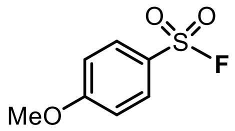

5

The **General Procedure A** was followed using 4-methoxybenzene sulfonyl chloride substrate (0.2 mmol, 41 mg) to afford **5** (34 mg, 90% yield) as a yellow solid. ^{1}H NMR (300 MHz, $CDCl_3$) δ 8.01 – 7.90 (m, 2H), 7.13 – 7.01 (m, 2H), 3.93 (s, 3H). ^{13}C NMR (75 MHz, $CDCl_3$) δ 165.4 (C_q), 131.0 (CH), 124.3 (d, $J_{C–F}$ = 24.7 Hz, C_q), 115.0 (CH), 56.1 (CH_3). ^{19}F NMR (282

MHz, $CDCl_3$) δ 67.2. **HR-MS** (ESI) m/z: $C_7H_7O_4S^-$ $[M-F+O]^-$ calculated for 187.0071, found 187.0076. This compound was known.[1]

[1,1′-Biphenyl]-4-sulfonyl fluoride (6)

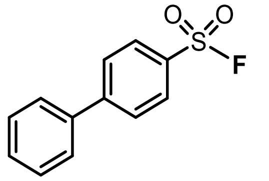

6

The **General Procedure A** was followed using [1,1′-biphenyl]-4-sulfonyl chloride substrate (0.2 mmol, 50 mg) to afford **6** (45 mg, 96% yield) as a yellow solid. ^{1}H NMR (300 MHz, $CDCl_3$) δ 8.14 – 8.03 (m, 2H), 7.88 – 7.77 (m, 2H), 7.69 – 7.60 (m, 2H), 7.58 – 7.43 (m, 3H). ^{13}C NMR (75 MHz, $CDCl_3$) δ 148.8 (C_q), 138.7 (C_q), 131.6 (d, J_{C-F} = 24.5 Hz, C_q), 129.4 (CH), 129.3 (CH), 129.1 (CH), 128.4 (CH), 127.6 (CH). ^{19}F NMR (282 MHz, $CDCl_3$) δ 66.5. **HR-MS** (ESI) m/z: $C_{12}H_9O_3S^-$ $[M-F+O]^-$ calculated for 233.0278, found 233.0275. This compound was known.[2]

3-Bromobenzenesulfonyl fluoride (7)

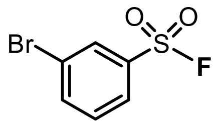

7

The **General Procedure A** was followed using 3-bromobenzenesulfonyl chloride substrate (0.2 mmol, 51 mg) to afford **7** (44 mg, 93% yield) as a yellow solid. ^{1}H NMR (300 MHz, $CDCl_3$) δ 8.21 – 8.13 (m, 1H), 8.01 – 7.87 (m, 2H), 7.63 – 7.47 (m, 1H). ^{13}C NMR (75 MHz, $CDCl_3$) δ 138.9 (CH), 134.9 (d, J_{C-F} = 25.5 Hz, C_q), 131.4 (CH), 131.3 (CH), 127.1 (CH), 123.7 (C_q). ^{19}F NMR (377 MHz, $CDCl_3$) δ 66.2. **HR-MS** (ESI) m/z: $C_6H_5BrFO_2S^+$ $[M+H]^+$ calculated for 238.9133, found 238.9172. This compound was known.[2]

2-Iodobenzenesulfonyl fluoride (8)

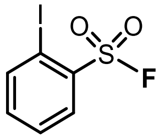

8

The **General Procedure A** was followed using 2-iodobenzenesulfonyl chloride substrate (0.2 mmol, 61 mg) to afford **8** (52 mg, 90% yield) as a yellow solid. ^{1}H NMR (400 MHz, $CDCl_3$) δ 8.24 – 8.11 (m, 2H), 7.65 – 7.56 (m, 1H), 7.43 – 7.35 (m, 1H). ^{13}C NMR (101 MHz, $CDCl_3$) δ 143.3 (CH), 137.8 (d, $J_{C–F}$ = 23.9 Hz, C_q), 135.9 (CH), 132.1 (CH), 128.8 (CH), 92.3 (C_q). ^{19}F NMR (282 MHz, $CDCl_3$) δ 56.7. **HR-MS** (ESI) m/z: $C_6H_5FIO_2S^+$ $[M+H]^+$ calculated for 286.9033, found 286.9035.

4-Chlorobenzenesulfonyl fluoride (9)

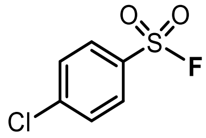

9

The **General Procedure A** was followed using 4-chlorobenzenesulfonyl chloride substrate (0.2 mmol, 42 mg) to afford **9** (37 mg, 95% yield) as a yellow solid. ^{1}H NMR (300 MHz, $CDCl_3$) δ 8.02 – 7.92 (m, 2H), 7.68 – 7.57 (m, 2H). ^{13}C NMR (75 MHz, $CDCl_3$) δ 142.8 (C_q), 131.59 (d, $J_{C–F}$ = 25.7 Hz, C_q), 130.3 (CH), 130.0 (CH). ^{19}F NMR (377 MHz, $CDCl_3$) δ 66.4. **HR-MS** (ESI) m/z: $C_6H_5ClFO_2S^+$ $[M+H]^+$ calculated for 194.9677, found 194.9677. This compound was known.[1]

2-Nitrobenzenesulfonyl fluoride (10)

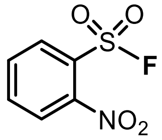

10

The **General Procedure A** was followed using 2-nitrobenzenesulfonyl chloride substrate (0.2 mmol, 44 mg) to afford **10** (37 mg, 90% yield) as a yellow solid. ^{1}H NMR (400 MHz, $CDCl_3$)

δ 8.29 – 8.22 (m, 1H), 8.08 – 8.03 (m, 1H), 8.00 – 7.94 (m, 1H), 7.93 – 7.86 (m, 1H). ^{13}C NMR (101 MHz, $CDCl_3$) δ 148.4 (C_q), 136.8 (CH), 133.4 (CH), 132.0 (CH), 127.3 (d, $J_{C\text{–}F}$ = 29.0 Hz, C_q), 126.1 (CH). ^{19}F NMR (471 MHz, $CDCl_3$) δ 65.0. **HR-MS** (ESI) m/z: $C_6H_5FNO_4S^+$ $[M+H]^+$ calculated for 205.9923, found 205.9918. This compound was known.[3]

2,4,6-Triisopropylbenzenesulfonyl fluoride (11)

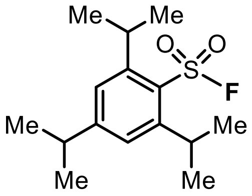

11

The **General Procedure A** was followed using 2,4,6-triisopropylbenzenesulfonyl chloride substrate (0.2 mmol, 60 mg) to afford **11** (53 mg, 92% yield) as a yellow solid. ^{1}H NMR (300 MHz, $CDCl_3$) δ 7.25 (s, 2H), 4.10 – 3.88 (m, 2H), 3.08 – 2.88 (m, 1H), 1.30 (t, J = 6.8 Hz, 18H). ^{13}C NMR (75 MHz, $CDCl_3$) δ 155.5 (C_q), 150.9 (C_q), 128.2 (d, $J_{C\text{–}F}$ = 18.7 Hz, C_q), 124.2 (CH), 34.6 (CH), 30.3 (2C, CH), 24.7 (CH_3), 23.7 (CH_3). ^{19}F NMR (282 MHz, $CDCl_3$) δ 73.4. **HR-MS** (ESI) m/z: $C_{15}H_{23}O_3S^-$ $[M\text{–}F+O]^-$ calculated for 283.1373, found 283.1368. This compound was known.[1]

2,4,6-Trichlorobenzenesulfonyl fluoride (12)

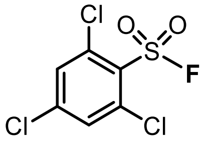

12

The **General Procedure A** was followed using 2,4,6-trichlorobenzenesulfonyl chloride substrate (0.2 mmol, 56 mg) to afford **12** (50 mg, 95% yield) as a yellow solid. ^{1}H NMR (300 MHz, $CDCl_3$) δ 7.59 (d, J = 0.8 Hz, 2H). ^{13}C NMR (75 MHz, $CDCl_3$) δ 141.4 (C_q), 136.9 (C_q), 131.5 (CH), 129.6 (d, $J_{C\text{–}F}$ = 25.5 Hz, C_q). ^{19}F NMR (282 MHz, $CDCl_3$) δ 68.6. **HR-MS** (ESI) m/z: $C_6H_2Cl_3O_3S^-$ $[M\text{–}F+O]^-$ calculated for 258.8796, found 258.8795.

Naphthalene-1-sulfonyl fluoride (13)

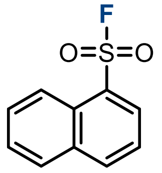

13

The **General Procedure A** was followed using naphthalene-1-sulfonylchloride substrate (0.2 mmol, 45 mg) to afford **13** (40 mg, 96% yield) as a yellow solid. ^{1}H NMR (300 MHz, $CDCl_3$) δ 8.62 – 8.50 (m, 1H), 8.43 – 8.34 (m, 1H), 8.28 – 8.20 (m, 1H), 8.06 – 7.97 (m, 1H), 7.84 – 7.74 (m, 1H), 7.74 – 7.58 (m, 2H). ^{13}C NMR (75 MHz, $CDCl_3$) δ 137.1 (C_q), 134.2 (C_q), 131.3 (CH), 129.7 (CH), 129.3 (d, $J_{C\text{–}F}$ = 23.5 Hz, C_q), 129.3 (CH), 128.5 (CH), 127.9 (CH), 124.4 (CH), 124.2 (CH). ^{19}F NMR (471 MHz, $CDCl_3$) δ 62.6. **HR-MS** (ESI) m/z: $C_{10}H_8FO_2S^+$ $[M+H]^+$ calculated for 211.0224, found 211.0224. This compound was known.[4]

(*E*)-2-Phenylethene-1-sulfonyl fluoride (14)

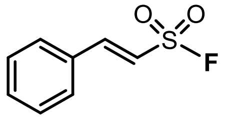

14

The **General Procedure A** was followed using (*E*)-2-phenylethene-1-sulfonyl chloride substrate (0.2 mmol, 40 mg) to afford **14** (34 mg, 90% yield) as a yellow solid. ^{1}H NMR (400 MHz, $CDCl_3$) δ 7.83 (dd, J = 15.6, 1.2 Hz, 1H), 7.60 – 7.46 (m, 5H), 6.88 (dd, J = 15.5, 2.5 Hz, 1H). ^{13}C NMR (101 MHz, $CDCl_3$) δ 149.0 (d, $J_{C\text{–}F}$ = 2.7 Hz, CH), 132.8 (CH), 131.2 (C_q), 129.6 (CH), 129.2 (CH), 118.1 (d, $J_{C\text{–}F}$ = 28.1 Hz, CH). ^{19}F NMR (377 MHz, $CDCl_3$) δ 62.3. **HR-MS** (ESI) m/z: $C_8H_8FO_2S^+$ $[M+H]^+$ calculated for 187.0224, found 187.0224. This compound was known.[3]

Pydirine-3-sulfonyl fluoride (15)

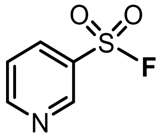

15

The **General Procedure A** was followed using pydirine-3-sulfonyl chloride substrate (0.2 mmol, 36 mg) to afford **15** (28 mg, 87% yield) as a yellow oil. ^{1}H NMR (300 MHz, $CDCl_3$) δ 9.28 – 9.19 (m, 1H), 9.01 (dd, J = 4.9, 1.6 Hz, 1H), 8.37 – 8.27 (m, 1H), 7.66 – 7.58 (m, 1H). ^{13}C NMR (75 MHz, $CDCl_3$) δ 156.1 (CH), 149.2 (CH), 136.2 (CH), 130.4 (d, J_{C-F} = 25.6 Hz, C_q), 124.3 (CH). ^{19}F NMR (282 MHz, $CDCl_3$) δ 67.9. **HR-MS** (ESI) m/z: $C_5H_4NO_3S^-$ $[M-F+O]^-$ calculated for 157.9917, found 157.9913. This compound was known.[5]

Thiophene-2-sulfonyl fluoride (16)

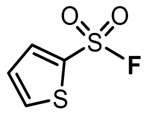

16

The **General Procedure A** was followed using thiophene-2-sulfonyl chloride substrate (0.2 mmol, 36 mg) to afford **16** (29 mg, 86% yield) as a yellow oil. ^{1}H NMR (300 MHz, $CDCl_3$) δ 7.97 – 7.91 (m, 1H), 7.91 – 7.84 (m, 1H), 7.27 – 7.21 (m, 1H). ^{13}C NMR (75 MHz, $CDCl_3$) δ 137.0 (CH), 136.6 (CH), 128.3 (CH). ^{19}F NMR (282 MHz, $CDCl_3$) δ 71.7. **HR-MS** (ESI) m/z: $C_4H_3O_3S_2^-$ $[M-F+O]^-$ calculated for 162.9529, found 162.9527. This compound was known.[1]

Quinoline-8-sulfonyl fluoride (17)

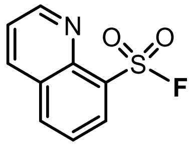

17

This compound was known.[1]The **General Procedure A** was followed using quinoline-8-sulfonyl chloride substrate (0.2 mmol, 45 mg) to afford **17** (38 mg, 91% yield) as a yellow solid. ^{1}H NMR (300 MHz, $CDCl_3$) δ 9.18 (dd, J = 4.3, 1.7 Hz, 1H), 8.52 (dd, J = 7.4, 1.4 Hz,

1H), 8.32 (dd, J = 8.4, 1.8 Hz, 1H), 8.24 (dd, J = 8.3, 1.4 Hz, 1H), 7.75 – 7.68 (m, 1H), 7.63 (dd, J = 8.4, 4.3 Hz, 1H). ^{13}C NMR (75 MHz, $CDCl_3$) δ 152.8 (CH), 143.9 (C_q), 136.7 (CH), 136.3 (CH), 133.2 (CH), 131.5 (d, $J_{C–F}$ = 21.0 Hz, C_q), 129.2 (C_q), 125.4 (CH), 123.2 (CH). ^{19}F NMR (282 MHz, $CDCl_3$) δ 60.2. **HR-MS** (ESI) m/z: $C_9H_6NO_3S^-$ $[M–F+O]^-$ calculated for 208.0074, found 208.0079.

2-Oxo-2*H*-chromene-6-sulfonyl fluoride (18)

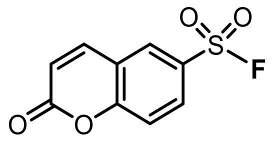

18

The **General Procedure A** was followed using 2-oxo-2*H*-chromene-6-sulfonyl chloride substrate (0.2 mmol, 49 mg) to afford **18** (33 mg, 73% yield) as a yellow solid. ^{1}H NMR (300 MHz, $CDCl_3$ δ 8.22 (d, J = 2.3 Hz, 1H), 8.14 (dd, J = 8.8, 2.3 Hz, 1H), 7.81 (d, J = 9.7 Hz, 1H), 7.56 (d, J = 8.8 Hz, 1H), 6.62 (d, J = 9.7 Hz, 1H). ^{13}C NMR (75 MHz, $CDCl_3$) δ 158.6 (C_q), 158.4 (C_q), 141.9 (CH), 131.3 (CH), 129.4 (CH), 129.1 (d, $J_{C–F}$ = 26.5 Hz, C_q), 119.6 (C_q), 119.4 (CH), 118.9 (CH). ^{19}F NMR (282 MHz, $CDCl_3$) δ 67.1. **HR-MS** (ESI) m/z: $C_9H_5O_5S^-$ $[M–F+O]^-$ calculated for 224.9863, found 224.9864.

Dodecane-1-sulfonyl fluoride (19)

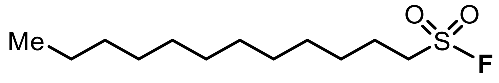

19

The **General Procedure A** was followed using dodecane-1-sulfonyl chloride substrate (0.2 mmol, 54 mg) to afford **19** (47 mg, 93% yield) as a yellow solid. ^{1}H NMR (300 MHz, $CDCl_3$) δ 3.45 – 3.29 (m, 2H), 2.07 – 1.90 (m, 2H), 1.54 – 1.44 (m, 2H), 1.40 – 1.18 (m, 16H), 0.93 – 0.83 (m, 3H). ^{13}C NMR (75 MHz, $CDCl_3$) δ 51.1 (d, $J_{C–F}$ = 16.1 Hz, CH_2), 32.1 (CH_2), 29.8 (CH_2), 29.7 (CH_2), 29.6 (CH_2), 29.5 (CH_2), 29.3 (CH_2), 29.0 (CH_2), 28.0 (CH_2), 23.6 (CH_2), 22.9 (CH_2), 14.3 (CH_3). ^{19}F NMR (282 MHz, $CDCl_3$) δ 53.2. **HR-MS** (ESI) m/z: $C_{12}H_{25}O_3S^-$ $[M–F+O]^-$ calculated for 249.1530, found 249.1527.

Methanesulfonyl fluoride (20)

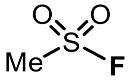

20

The **General Procedure A** was followed using methanesulfonyl chloride substrate (0.2 mmol, 23 mg) to afford **20**. Product is volatile so ^{1}H NMR yield is reported. ^{1}H NMR (300 MHz, $CDCl_3$) δ 3.28 (d, *J* = 5.1 Hz, 3H). ^{19}F NMR (282 MHz, $CDCl_3$) δ 61.6. This compound was known.[6]

((1*S*,4*R*)-7,7-dimethyl-2-oxobicyclo[2.2.1]heptan-1-yl)methanesulfonyl fluoride (21)

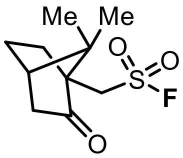

21

The **General Procedure A** was followed using *L*-(-)-10-camphorsulfonyl chloride substrate (0.2 mmol, 47 mg) to afford **21** (34 mg, 78% yield) as a yellow solid. ^{1}H NMR (300 MHz, $CDCl_3$) δ 3.87 (dd, *J* = 15.2, 2.5 Hz, 1H), 3.30 (dd, *J* = 15.2, 2.9 Hz, 1H), 2.50 – 2.32 (m, 2H), 2.21 – 2.07 (m, 2H), 2.05 – 1.97 (m, 1H), 1.80 – 1.71 (m, 1H), 1.52 – 1.44 (m, 1H), 1.14 (s, 3H), 0.93 (s, 3H). ^{13}C NMR (75 MHz, $CDCl_3$) δ 213.2 (C_q), 58.0 (C_q), 48.5 (d, $J_{C–F}$ = 18.3 Hz, CH_2), 48.2 (C_q), 43.1 (CH_2), 42.5 (CH), 27.0 (CH_2), 25.4 (CH_2), 19.9 (CH_3), 19.8 (CH_3). ^{19}F NMR (282 MHz, $CDCl_3$) δ 64.2. **HR-MS** (ESI) m/z: $C_{10}H_{15}O_4S^–$ $[M–F–H+2O]^–$ calculated for 231.0697, found 231.0703.

Cyclopropanesulfonyl fluoride (22)

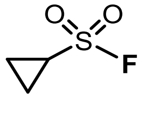

22

The **General Procedure A** was followed using cyclopropanesulfonyl chloride substrate (0.2 mmol, 28 mg) to afford **22**. Product is volatile so ^{1}H NMR yield is reported. ^{1}H NMR (300 MHz, $CDCl_3$) δ 2.79 – 2.63 (m, 1H), 1.52 – 1.41 (m, 2H), 1.35 – 1.29 (m, 2H). ^{19}F NMR (282 MHz, $CDCl_3$) δ 57.4.

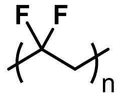

23, PVDF100
M_w = 100 kg/mol

The **General Procedure A** was followed using PVDF (0.3 mmol, 1.5 equiv., 19 mg, M_w = 100 kg/mol), *t*BuOK (0.3 mmol, 1.5 equiv., 34 mg) and tosyl chloride substrate (0.2 mmol, 38 mg) to afford **2** (34 mg, 98% yield) as a yellow solid. ^{1}H NMR (300 MHz, $CDCl_3$) δ 7.99 – 7.81 (m, 2H), 7.49 – 7.39 (m, 2H), 2.50 (s, 3H). ^{19}F NMR (282 MHz, $CDCl_3$) δ 66.2.

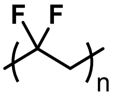

24, PVDF600
M_w = 600 kg/mol

The **General Procedure A** was followed using PVDF (0.3 mmol, 1.5 equiv., 19 mg, M_w = 600 kg/mol), *t*BuOK (0.3 mmol, 1.5 equiv., 34 mg) and tosyl chloride substrate (0.2 mmol, 38 mg) to afford **2** (33 mg, 96% yield) as a yellow solid. ^{1}H NMR (300 MHz, $CDCl_3$) δ 7.99 – 7.81 (m, 2H), 7.49 – 7.39 (m, 2H), 2.50 (s, 3H). ^{19}F NMR (282 MHz, $CDCl_3$) δ 66.2.

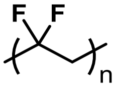

25, PVDF1000
M_w = 1000 kg/mol

The **General Procedure A** was followed using PVDF (0.3 mmol, 1.5 equiv., 19 mg, M_w = 1000 kg/mol), *t*BuOK (0.3 mmol, 1.5 equiv., 34 mg) and tosyl chloride substrate (0.2 mmol, 38 mg) to afford **2** (33 mg, 96% yield) as a yellow solid. ^{1}H NMR (300 MHz, $CDCl_3$) δ 7.99 – 7.81 (m, 2H), 7.49 – 7.39 (m, 2H), 2.50 (s, 3H). ^{19}F NMR (282 MHz, $CDCl_3$) δ 66.2.

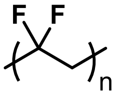

26, PVDF1800
M_w = 1800 kg/mol

The **General Procedure A** was followed using PVDF (0.3 mmol, 1.5 equiv., 19 mg, M_w = 1800 kg/mol), *t*BuOK (0.3 mmol, 1.5 equiv., 34 mg) and tosyl chloride substrate (0.2 mmol, 38 mg) to afford **2** (32 mg, 91% yield) as a yellow solid. ^{1}H NMR (300 MHz, $CDCl_3$) δ 7.99 – 7.81 (m, 2H), 7.49 – 7.39 (m, 2H), 2.50 (s, 3H). ^{19}F NMR (282 MHz, $CDCl_3$) δ 66.2.

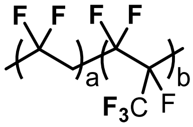

27, P(VDF-HFP)

The **General Procedure A** was followed using P(VDF-HFP) (100 mg), *t*BuOK (0.3 mmol, 1.5 equiv., 34 mg) and tosyl chloride substrate (0.2 mmol, 38 mg) to afford **2** (33 mg, 96% yield) as a yellow solid. ^{1}H NMR (300 MHz, $CDCl_3$) δ 7.99 – 7.81 (m, 2H), 7.49 – 7.39 (m, 2H), 2.50 (s, 3H). ^{19}F NMR (282 MHz, $CDCl_3$) δ 66.2.

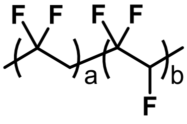

28, P(VDF-TrFE)
(piezoelectric materials)

The **General Procedure A** was followed using P(VDF-TrFE) (100 mg), *t*BuOK (0.3 mmol, 1.5 equiv., 34 mg) and tosyl chloride substrate (0.2 mmol, 38 mg) to afford **2** (33 mg, 96% yield) as a yellow solid. ^{1}H NMR (300 MHz, $CDCl_3$) δ 7.99 – 7.81 (m, 2H), 7.49 – 7.39 (m, 2H), 2.50 (s, 3H). ^{19}F NMR (282 MHz, $CDCl_3$) δ 66.2.

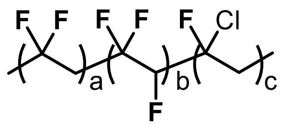

29, P(VDF-TrFE-CFE)
(ferroelectric materials)

The **General Procedure A** was followed using P(VDF-TrFE-CFE) (100 mg), *t*BuOK (0.3 mmol, 1.5 equiv., 34 mg) and tosyl chloride substrate (0.2 mmol, 38 mg) to afford **2** (32 mg, 93% yield) as a yellow solid. ^{1}H NMR (300 MHz, $CDCl_3$) δ 7.99 – 7.81 (m, 2H), 7.49 – 7.39 (m, 2H), 2.50 (s, 3H). ^{19}F NMR (282 MHz, $CDCl_3$) δ 66.2.

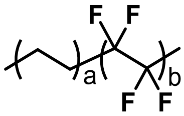

30, ETFE (Dyneon)

The **General Procedure A** was followed using ETFE (100 mg), *t*BuOK (0.3 mmol, 1.5 equiv., 34 mg) and tosyl chloride substrate (0.2 mmol, 38 mg) to afford **2** (31 mg, 90% yield) as a yellow solid. ^{1}H NMR (300 MHz, $CDCl_3$) δ 7.99 – 7.81 (m, 2H), 7.49 – 7.39 (m, 2H), 2.50 (s, 3H). ^{19}F NMR (282 MHz, $CDCl_3$) δ 66.2.

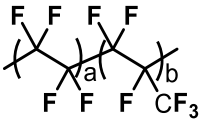

31, FEP (F46)

The **General Procedure B** was followed using FEP (40 mg), *t*BuOK (0.3 mmol, 1.5 equiv., 34 mg) and tosyl chloride substrate (0.2 mmol, 38 mg) to afford **2** (27 mg, 78% yield) as a yellow solid. ^{1}H NMR (300 MHz, $CDCl_3$) δ 7.99 – 7.81 (m, 2H), 7.49 – 7.39 (m, 2H), 2.50 (s, 3H). ^{19}F NMR (282 MHz, $CDCl_3$) δ 66.2.

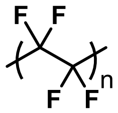

32, PTFE (Teflon)

The **General Procedure B** was followed using PTFE (0.4 mmol, 2 equiv., 40 mg), *t*BuOK (0.3 mmol, 1.5 equiv., 34 mg) and tosyl chloride substrate (0.2 mmol, 38 mg) to afford **2** (30 mg, 88% yield) as a yellow solid. ^{1}H NMR (300 MHz, $CDCl_3$) δ 7.99 – 7.81 (m, 2H), 7.49 – 7.39 (m, 2H), 2.50 (s, 3H). ^{19}F NMR (282 MHz, $CDCl_3$) δ 66.2.

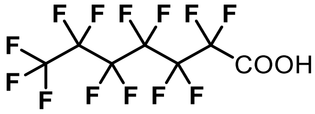

33, PFHA

The **General Procedure B** was followed using PFHA (0.3 mmol, 1.5 equiv., 109 mg), *t*BuOK (0.3 mmol, 1.5 equiv., 34 mg) and tosyl chloride substrate (0.2 mmol, 38 mg) to afford **2** (24 mg, 70% yield) as a yellow solid. ^{1}H NMR (300 MHz, $CDCl_3$) δ 7.99 – 7.81 (m, 2H), 7.49 – 7.39 (m, 2H), 2.50 (s, 3H). ^{19}F NMR (282 MHz, $CDCl_3$) δ 66.2.

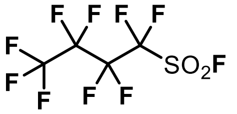

34, NFBS

The **General Procedure B** was followed using NFBS (0.3 mmol, 1.5 equiv., 60 mg), *t*BuOK (0.3 mmol, 1.5 equiv., 34 mg) and tosyl chloride substrate (0.2 mmol, 38 mg) to afford **2** (20 mg, 58% yield) as a yellow solid. ^{1}H NMR (300 MHz, $CDCl_3$) δ 7.99 – 7.81 (m, 2H), 7.49 – 7.39 (m, 2H), 2.50 (s, 3H). ^{19}F NMR (282 MHz, $CDCl_3$) δ 66.2.

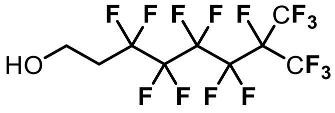

35, PFMO

The **General Procedure B** was followed using PFMO (0.3 mmol, 1.5 equiv., 83 mg), *t*BuOK (0.3 mmol, 1.5 equiv., 34 mg) and tosyl chloride substrate (0.2 mmol, 38 mg) to afford **2** (18

mg, 52% yield) as a yellow solid. ^{1}H NMR (300 MHz, $CDCl_3$) δ 7.99 – 7.81 (m, 2H), 7.49 – 7.39 (m, 2H), 2.50 (s, 3H). ^{19}F NMR (282 MHz, $CDCl_3$) δ 66.2.

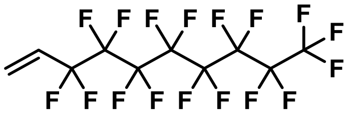

36, PFDE

The **General Procedure B** was followed using PFDE (0.3 mmol, 1.5 equiv., 134 mg), *t*BuOK (0.3 mmol, 1.5 equiv., 34 mg) and tosyl chloride substrate (0.2 mmol, 38 mg) to afford **2** (27 mg, 77% yield) as a yellow solid. ^{1}H NMR (300 MHz, $CDCl_3$) δ 7.99 – 7.81 (m, 2H), 7.49 – 7.39 (m, 2H), 2.50 (s, 3H). ^{19}F NMR (282 MHz, $CDCl_3$) δ 66.2.

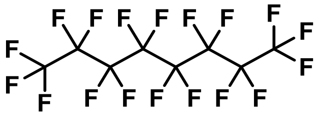

37, PFO

The **General Procedure B** was followed using PFO (0.3 mmol, 1.5 equiv., 88 mg), *t*BuOK (0.3 mmol, 1.5 equiv., 34 mg) and tosyl chloride substrate (0.2 mmol, 38 mg) to afford **2** (18 mg, 51% yield) as a yellow solid. ^{1}H NMR (300 MHz, $CDCl_3$) δ 7.99 – 7.81 (m, 2H), 7.49 – 7.39 (m, 2H), 2.50 (s, 3H). ^{19}F NMR (282 MHz, $CDCl_3$) δ 66.2.

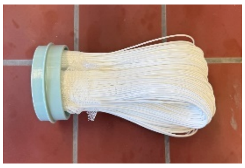

The **General Procedure A** was followed using sewage filter (25 mg), *t*BuOK (0.3 mmol, 1.5 equiv., 34 mg) and tosyl chloride substrate (0.2 mmol, 38 mg) to afford **2** (32 mg, 93% yield) as a yellow solid. ^{1}H NMR (300 MHz, $CDCl_3$) δ 7.99 – 7.81 (m, 2H), 7.49 – 7.39 (m, 2H), 2.50 (s, 3H). ^{19}F NMR (282 MHz, $CDCl_3$) δ 66.2.

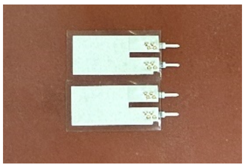

The **General Procedure A** was followed using sensor (25 mg), *t*BuOK (0.3 mmol, 1.5 equiv., 34 mg) and tosyl chloride substrate (0.2 mmol, 38 mg) to afford **2** (33 mg, 94% yield) as a yellow solid. ^{1}H NMR (300 MHz, $CDCl_3$) δ 7.99 – 7.81 (m, 2H), 7.49 – 7.39 (m, 2H), 2.50 (s, 3H). ^{19}F NMR (282 MHz, $CDCl_3$) δ 66.2.

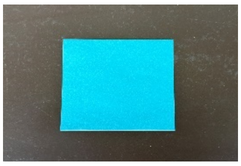

The **General Procedure A** was followed using transfer membrane (25 mg), *t*BuOK (0.3 mmol, 1.5 equiv., 34 mg) and tosyl chloride substrate (0.2 mmol, 38 mg) to afford **2** (34 mg, 98% yield) as a yellow solid. ^{1}H NMR (300 MHz, $CDCl_3$) δ 7.99 – 7.81 (m, 2H), 7.49 – 7.39 (m, 2H), 2.50 (s, 3H). ^{19}F NMR (282 MHz, $CDCl_3$) δ 66.2.

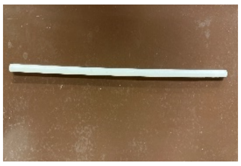

The **General Procedure A** was followed using PVDF rod (25 mg), *t*BuOK (0.3 mmol, 1.5 equiv., 34 mg) and tosyl chloride substrate (0.2 mmol, 38 mg) to afford **2** (32 mg, 92% yield) as a yellow solid. ^{1}H NMR (300 MHz, $CDCl_3$) δ 7.99 – 7.81 (m, 2H), 7.49 – 7.39 (m, 2H), 2.50 (s, 3H). ^{19}F NMR (282 MHz, $CDCl_3$) δ 66.2.

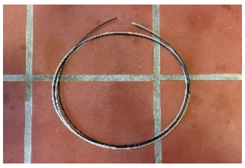

The **General Procedure A** was followed using infusion tube (25 mg), *t*BuOK (0.3 mmol, 1.5 equiv., 34 mg) and tosyl chloride substrate (0.2 mmol, 38 mg) to afford **2** (34 mg, 97% yield)

as a yellow solid. ^{1}H NMR (300 MHz, $CDCl_3$) δ 7.99 – 7.81 (m, 2H), 7.49 – 7.39 (m, 2H), 2.50 (s, 3H). ^{19}F NMR (282 MHz, $CDCl_3$) δ 66.2.

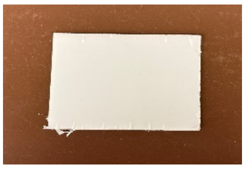

The **General Procedure A** was followed using PVDF plate (25 mg), *t*BuOK (0.3 mmol, 1.5 equiv., 34 mg) and tosyl chloride substrate (0.2 mmol, 38 mg) to afford **2** (30 mg, 85% yield) as a yellow solid. ^{1}H NMR (300 MHz, $CDCl_3$) δ 7.99 – 7.81 (m, 2H), 7.49 – 7.39 (m, 2H), 2.50 (s, 3H). ^{19}F NMR (282 MHz, $CDCl_3$) δ 66.2.

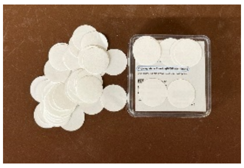

The **General Procedure A** was followed using filter membrane (25 mg), *t*BuOK (0.3 mmol, 1.5 equiv., 34 mg) and tosyl chloride substrate (0.2 mmol, 38 mg) to afford **2** (33 mg, 96% yield) as a yellow solid. ^{1}H NMR (300 MHz, $CDCl_3$) δ 7.99 – 7.81 (m, 2H), 7.49 – 7.39 (m, 2H), 2.50 (s, 3H). ^{19}F NMR (282 MHz, $CDCl_3$) δ 66.2.

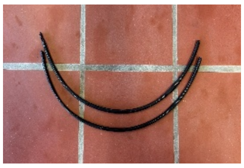

The **General Procedure A** was followed using plastic pipe (25 mg), *t*BuOK (0.3 mmol, 1.5 equiv., 34 mg) and tosyl chloride substrate (0.2 mmol, 38 mg) to afford **2** (33 mg, 95% yield) as a yellow solid. ^{1}H NMR (300 MHz, $CDCl_3$) δ 7.99 – 7.81 (m, 2H), 7.49 – 7.39 (m, 2H), 2.50 (s, 3H). ^{19}F NMR (282 MHz, $CDCl_3$) δ 66.2.

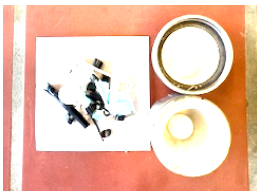

The **General Procedure A** was followed using an equal mixture of 8 types of PVDF (1 g), *t*BuOK (5 mmol, 1.5 equiv., 561 mg) and tosyl chloride substrate (3.33 mmol, 634 mg) to afford **2** (940 mg, 90% yield) as a yellow solid. ^{1}H NMR (300 MHz, $CDCl_3$) δ 7.99 – 7.81 (m, 2H), 7.49 – 7.39 (m, 2H), 2.50 (s, 3H). ^{19}F NMR (282 MHz, $CDCl_3$) δ 66.2.

1-Tosylpyrene (46)

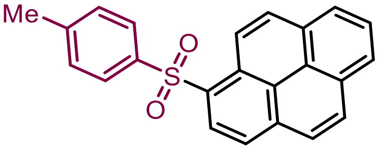

46

To a solution of 1-bromopyrene (4.5 mmol) in anhydrous THF (20 mL) at −78 ºC, was added *n*-BuLi (2.5 M in hexanes, 4.5 mmol, 1.8 mL) dropwise. The mixture was stirred at −78 ºC for 1 h and then transferred to a pre-cooled solution of TsF (3.0 mmol) in anhydrous THF (10 mL). The solution was allowed to warm to RT over 1 h hand stirred for a further 3 h. The reaction was then quenched with saturated aqueous NH_4Cl and the organic phase was extracted with ethyl acetate (3 x 20 mL). The combined organic phases were dried over $MgSO_4$, concentrated under reduced pressure. The crude mixture was purified using flash column chromatography (ethyl acetate/petroleum ether = 1:5) to afford the desired product **46** as a yellow solid in an 84 % yield (900 mg). ^{1}H NMR (300 MHz, $CDCl_3$) δ 9.01 (d, *J* = 9.4 Hz, 1H), 8.94 (d, *J* = 8.3 Hz, 1H), 8.28 – 8.15 (m, 5H), 8.08 – 8.03 (m, 2H), 7.96 – 7.89 (m, 2H), 7.25 (d, *J* = 8.0 Hz, 2H), 2.37 – 2.29 (m, 3H). ^{13}C NMR (75 MHz, $CDCl_3$) δ 143.9 (C_q), 139.8 (C_q), 135.4 (C_q), 132.5 (C_q), 130.9 (C_q), 130.6 (CH), 130.2 (CH), 130.1 (C_q), 129.8 (CH), 128.7 (C_q), 127.5 (CH), 127.3 (CH), 127.1 (CH), 127.1 (CH), 127.0 (CH), 126.9 (CH), 125.2 (C_q), 124.3 (CH), 124.0 (C_q), 123.0 (CH), 21.6 (CH_3). **HR-MS** (ESI) m/z: $C23H17O2S^+$ $[M+H]^+$ calculated for 357.0944, found 357.0942.

N-(2-(5-Methoxy-1-tosyl-1*H*-indol-3-yl)ethyl)acetamide (47)

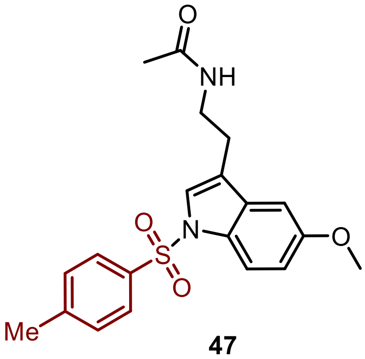

47

To a reaction flask was added TsF (0.5 mmol, 1.0 equiv.), melatonin (0.5 mmol, 1.0 equiv.) and BTMG (0.6 mmol, 1.2 equiv.) in MeCN (2 mL). The reaction was left to stir at room temperature for 24 h. The reaction mixture was then concentrated under reduced pressure, and purified by flash column chromatograph (DCM/MeOH = 20:1) to afford the tosylated product **47** as a colorless oil in 85 % yield (164 mg). ^{1}H NMR (300 MHz, $CDCl_3$) δ 7.89 – 7.80 (m, 1H), 7.74 – 7.60 (m, 2H), 7.30 (s, 1H), 7.14 (d, *J* = 8.1 Hz, 2H), 6.97 – 6.86 (m, 2H), 6.23 (t, *J* = 5.9 Hz, 1H), 3.76 (s, 3H), 3.47 (q, *J* = 6.7 Hz, 2H), 2.87 – 2.73 (m, 2H), 2.27 (s, 3H), 1.89 (s, 3H). ^{13}C NMR (75 MHz, $CDCl_3$) δ 170.4 (CO), 156.5 (C_q), 144.9 (C_q), 135.0 (C_q), 131.9 (C_q), 129.9 (C_q), 129.8 (CH), 126.6 (CH), 124.0 (CH), 120.3 (C_q), 114.6 (CH), 113.8 (CH), 102.0 (CH), 55.7 (CH_3), 38.8 (CH_2), 25.1 (CH_2), 23.2 (CH_3), 21.5 (CH_3). **HR-MS** (ESI) m/z: C20H23N2O4S^{+} [M+H]$^{+}$ calculated for 387.1373, found 387.1371.

(3a*R*,5*R*,6*S*,6a*R*)-5-((*R*)-2,2-Dimethyl-1,3-dioxolan-4-yl)-2,2-dimethyltetrahydrofuro[2,3-*d*][1,3]dioxol-6-yl 4-methylbenzenesulfonate (48)

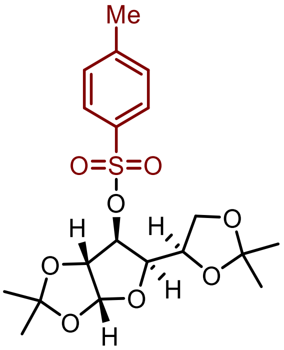

48

To a reaction flask was added TsF (0.2 mmol), diacetone-*d*-glucose (0.2 mmol) HMDS (0.2 mmol) and BTMG (0.2 mmol) in MeCN/DCM ((1/2 mL). The reaction was left to stir at room temperature for 12 h. The reaction mixture was then concentrated under reduced pressure, and

purified by flash column chromatograph to afford the tosylated product **48** as a colorless oil in a 72 % yield (60 mg). ^{1}H NMR (300 MHz, $CDCl_3$) δ 7.87 – 7.73 (m, 2H), 7.33 (d, *J* = 8.1 Hz, 2H), 5.91 (d, *J* = 3.7 Hz, 1H), 4.88 – 4.74 (m, 2H), 4.10 – 3.94 (m, 3H), 3.93 – 3.84 (m, 1H), 2.44 (s, 3H), 1.47 (s, 3H), 1.30 (s, 3H), 1.18 (s, 3H), 1.14 (s, 3H). ^{13}C NMR (75 MHz, $CDCl_3$) δ 145.2 (C_q), 132.8 (C_q), 129.8 (CH), 128.6 (CH), 112.6 (C_q), 109.2 (C_q), 105.2 (CH), 83.4 (CH), 82.2 (CH), 80.0 (CH), 71.9 (CH), 67.2 (CH_2), 26.7 (CH_3), 26.7 (CH_3), 26.3 (CH_3), 25.0 (CH_3), 21.8 (CH_3). **HR-MS** (ESI) m/z: C19H27O8S$^+$ [M+H]$^+$ calculated for 415.1421, found 415.1422.

(*S*)-(6-Methoxyquinolin-4-yl)((1*S*,2*R*,4*S*,5*R*)-5-vinylquinuclidin-2-yl)methyl 4-methylbenzenesulfonate

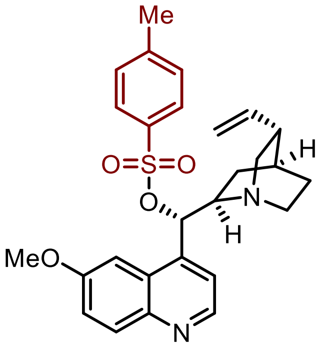

49

To a reaction flask was added TsF (0.2 mmol), quinidine (0.2 mmol) HMDS (0.2 mmol) and BTMG (0.2 mmol) in MeCN/DCM ((1/2 mL). The reaction was left to stir at room temperature for 24 h. The reaction mixture was then concentrated under reduced pressure, and purified by flash column chromatograph to afford the tosylated product **49** as a colorless oil in a 67 % yield (64 mg).^{1}H NMR (300 MHz, $CDCl_3$) δ 8.54 (d, *J* = 4.5 Hz, 1H), 7.89 (d, *J* = 9.2 Hz, 1H), 7.51 – 7.28 (m, 3H), 7.25 – 6.96 (m, 2H), 6.93 – 6.71 (m, 2H), 6.36 – 5.74 (m, 2H), 5.16 – 5.00 (m, 2H), 3.94 (s, 3H), 3.25 (s, 1H), 2.95 – 2.77 (m, 2H), 2.75 – 2.52 (m, 2H), 2.18 (s, 4H), 2.00 – 1.88 (m, 1H), 1.87 – 1.80 (m, 1H), 1.74 – 1.61 (m, 1H), 1.60 – 1.46 (m, 2H). ^{13}C NMR (75 MHz, $CDCl_3$) δ 157.9 (C_q), 147.1 (CH), 144.8 (C_q), 144.5 (C_q), 140.1 (CH), 133.3 (C_q), 131.8 (CH), 129.1 (CH), 127.7 (CH), 126.7 (CH), 121.8 (CH), 118.9 (C_q), 115.2 (CH_2), 100.6 (C_q), 80.1 (CH), 60.5 (CH), 55.8 (CH_3), 49.9 (CH_2), 49.0 (CH_2), 39.9 (CH), 27.8 (CH), 26.4 (CH_2), 24.1 (CH_2), 21.5 (CH_3). **HR-MS** (ESI) m/z: C27H31N2O4S$^+$ [M+H]$^+$ calculated for 479.1999, found 479.1998.

(*R*)-2,8-Dimethyl-2-((4*R*,8*R*)-4,8,12-trimethyltridecyl)chroman-6-yl 4-methylbenzenesulfonate

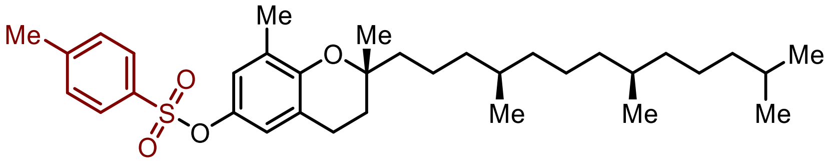

50

To a reaction flask was added TsF (0.2 mmol), δ-tocopherol (0.2 mmol) HMDS (0.2 mmol) and BTMG (0.2 mmol) in MeCN (1 mL). The reaction was left to stir at room temperature for 12 h. The reaction mixture was then concentrated under reduced pressure, and purified by flash column chromatograph to afford the tosylated product **50** as a colorless oil in a 91 % yield (101 mg). ^{1}H NMR (300 MHz, $CDCl_3$) δ 7.77 – 7.69 (m, 2H), 7.32 (d, J = 8.1 Hz, 2H), 6.61 – 6.50 (m, 2H), 2.71 – 2.61 (m, 2H), 2.46 (s, 3H), 2.06 (s, 3H), 1.85 – 1.65 (m, 2H), 1.65 – 1.00 (m, 24H), 0.92 – 0.84 (m, 12H). ^{13}C NMR (75 MHz, $CDCl_3$) δ 150.9 (C_q), 145.1 (C_q), 141.5 (C_q), 133.0 (C_q), 129.7 (CH), 128.7 (CH), 127.6 (C_q), 122.0 (CH), 121.3 (C_q), 120.3 (CH), 76.6 (C_q), 40.3 (CH_2), 39.5 (CH_2), 37.6 (CH_2), 37.6 (CH_2), 37.4 (CH_2), 33.0 (CH), 32.8 (CH), 31.0 (CH_2), 28.1(CH), 25.0 (CH_2), 24.6 (CH_2), 24.3 (CH_3), 22.9 (CH_3), 22.8 (CH_3), 22.5 (CH_2), 21.9 (CH_3), 21.1 (CH_2), 19.9 (CH_3), 19.8 (CH_3), 16.2 (CH_3). **HR-MS** (ESI) m/z: $C34H52NaO4S^{+}$ $[M+Na]^{+}$ calculated for 579.3479, found 579.3480.

(4*S*,5'*R*,6a*R*,6b*S*,8a*S*,8b*R*,9*S*,10*R*,11a*S*,12a*S*,12b*S*)-5',6a,8a,9-Tetramethyl-1,3,3',4,4',5,5',6,6a,6b,6',7,8,8a,8b,9,11a,12,12a,12b-icosahydrospiro[naphtho[2',1':4,5]indeno[2,1-*b*]furan-10,2'-pyran]-4-yl 4-methylbenzenesulfonate (51)

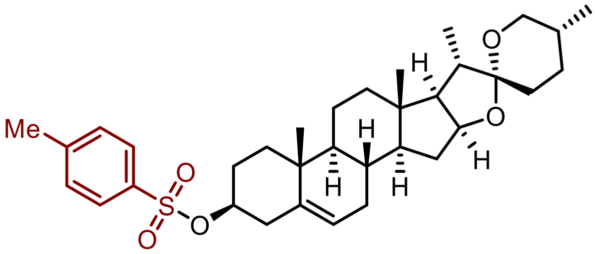

51

To a reaction flask was added TsF (0.3 mmol), Diosgenin (0.2 mmol) HMDS (0.4 mmol) and BTMG (0.4 mmol) in MeCN/DCM ((1/2 mL). The reaction was left to stir at 60 °C for 18 h. The reaction mixture was then concentrated under reduced pressure, and purified by flash column chromatograph to afford the tosylated product **51** as a colorless oil in a 62 % yield (70

mg). ^{1}H NMR (300 MHz, $CDCl_3$) δ 7.79 (d, J = 8.2 Hz, 2H), 7.32 (d, J = 8.1 Hz, 2H), 5.36 – 5.22 (m, 1H), 4.49 – 4.22 (m, 2H), 3.55 – 3.29 (m, 2H), 2.50 – 2.36 (m, 4H), 2.33 – 2.19 (m, 1H), 2.05 – 1.93 (m, 2H), 1.85 – 1.55 (m, 11H), 1.50 – 1.40 (m, 3H), 1.31 – 0.90 (m, 12H), 0.82 – 0.73 (m, 6H).

^{13}C NMR (75 MHz, $CDCl_3$) δ 144.5 (C_q), 139.0 (C_q), 134.8 (C_q), 129.9 (CH), 127.8 (CH), 123.4 (CH), 109.44 (C_q), 82.4 (CH), 80.9 (CH), 67.0 (CH_2), 62.2 (CH), 56.5 (CH), 50.0 (CH), 41.7 (CH), 40.4 (Cq), 39.8 (CH_2), 39.0 (CH_2), 37.0 (Cq), 36.6 (CH_2), 32.1 (CH_2), 31.9 (CH_2), 31.5 (CH_2), 31.4 (CH), 30.4 (CH), 28.9 (CH_2), 28.7 (CH_2), 21.8 (CH_3), 20.9 (CH_2), 19.3 (CH_3), 17.3 (CH_3), 16.4 (CH_3), 14.7 (CH_3). **HR-MS** (ESI) m/z: $C34H49O5S^{+}$ $[M+H]^{+}$ calculated for 569.3295, found 569.3299.

6 NMR Spectra

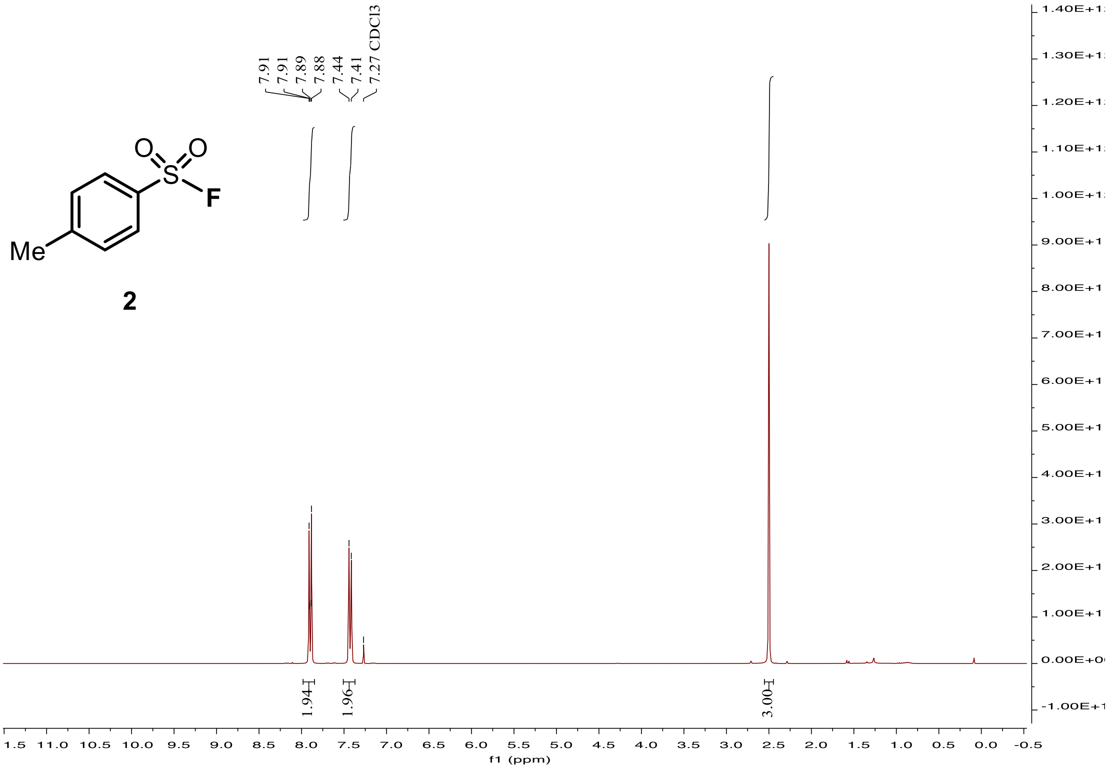

Supplementary Figure 12. ^{1}H-NMR (300 MHz, $CDCl_3$) spectrum of **2**

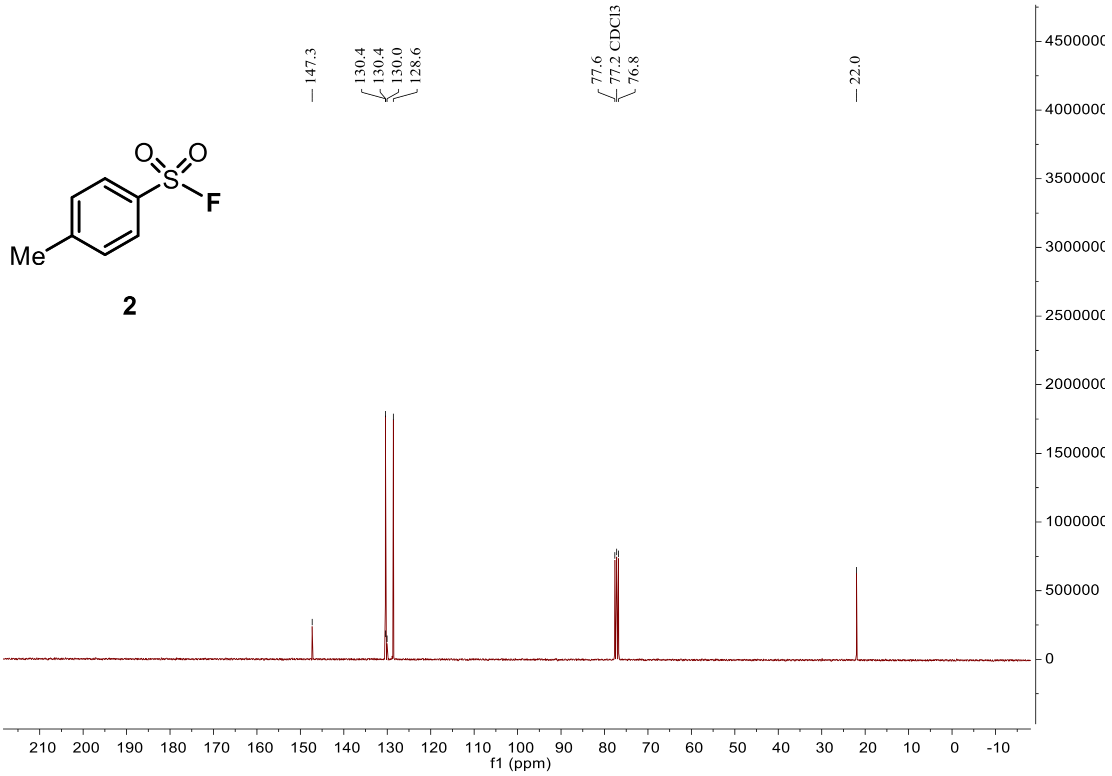

Supplementary Figure 13. ^{13}C-NMR (75 MHz, $CDCl_3$) spectrum of **2**

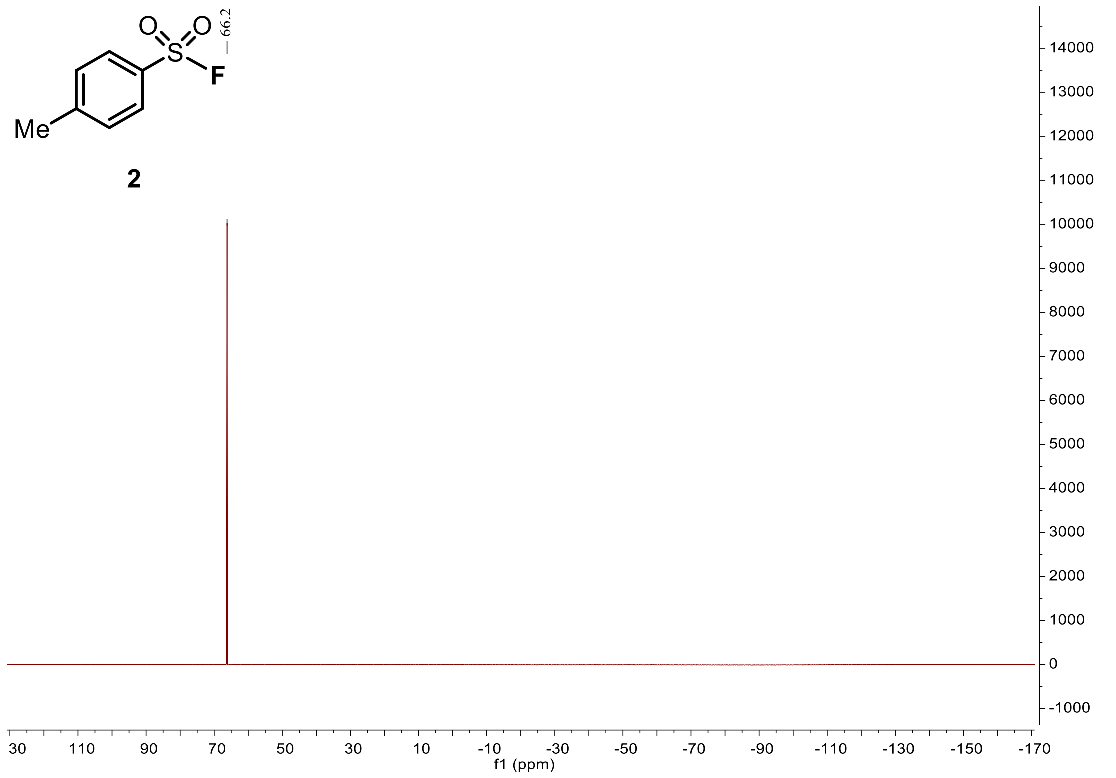

Supplementary Figure 14. ^{19}F-NMR (282 MHz, $CDCl_3$) spectrum of **2**

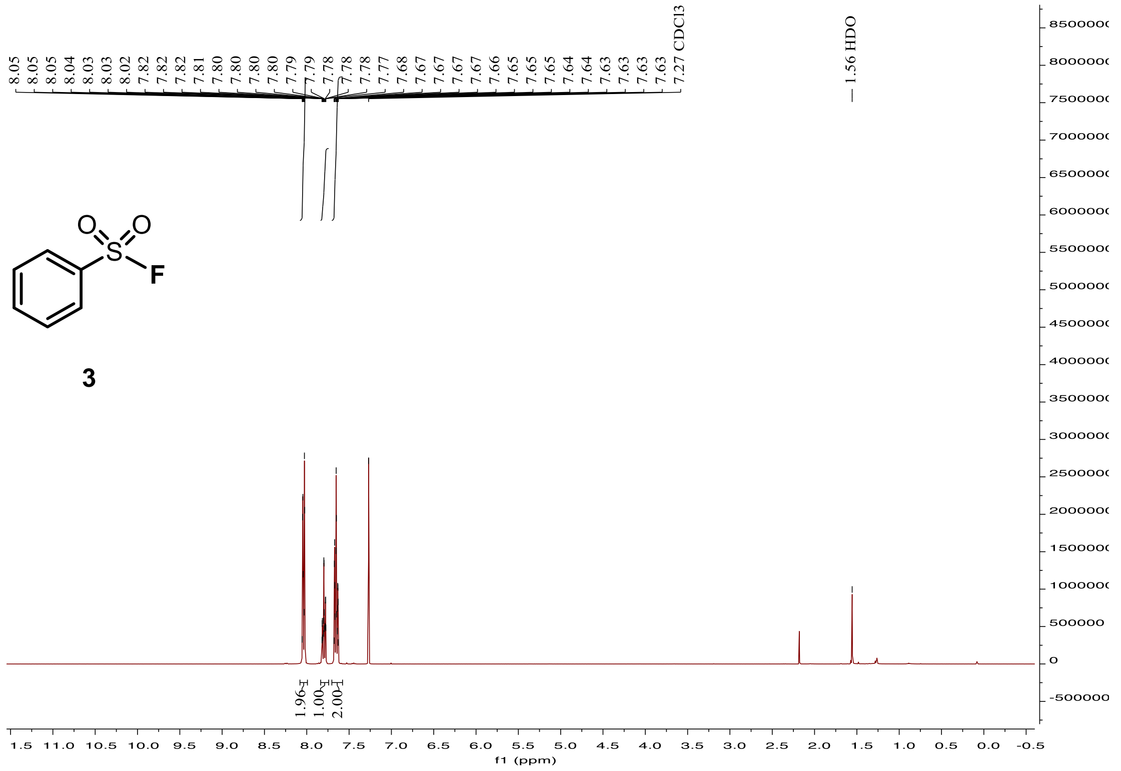

Supplementary Figure 15. ^{1}H-NMR (300 MHz, $CDCl_3$) spectrum of **3**

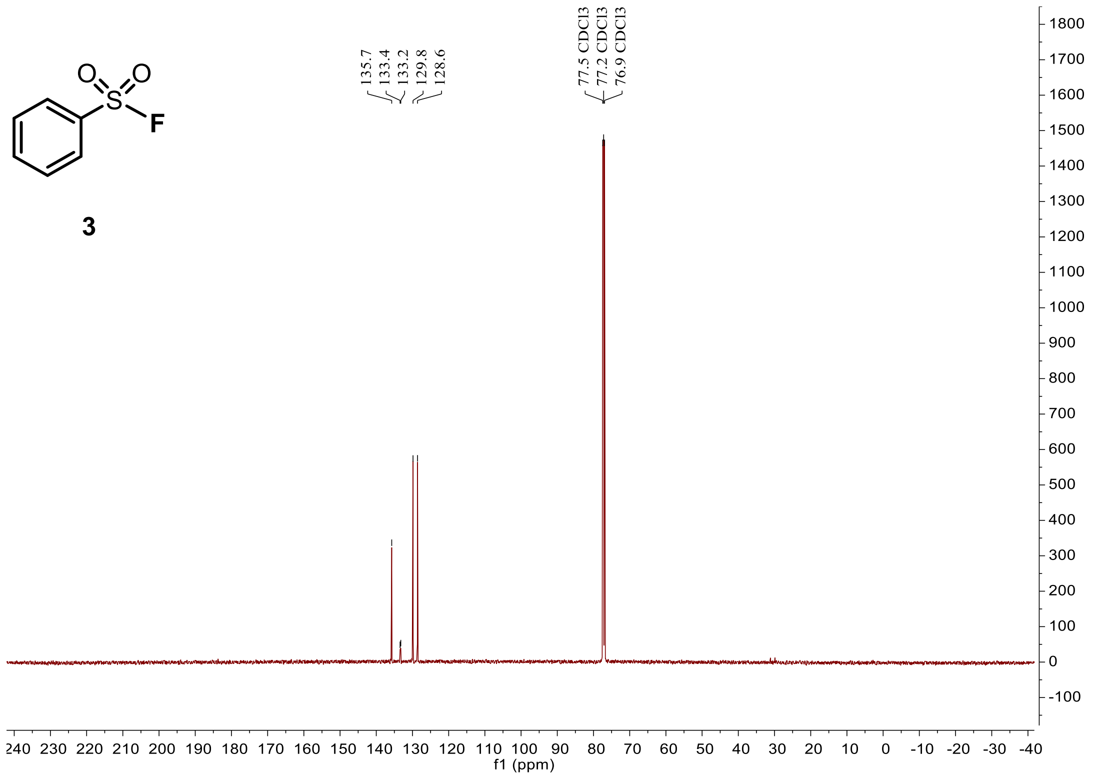

Supplementary Figure 16. ^{13}C-NMR (75 MHz, $CDCl_3$) spectrum of **3**

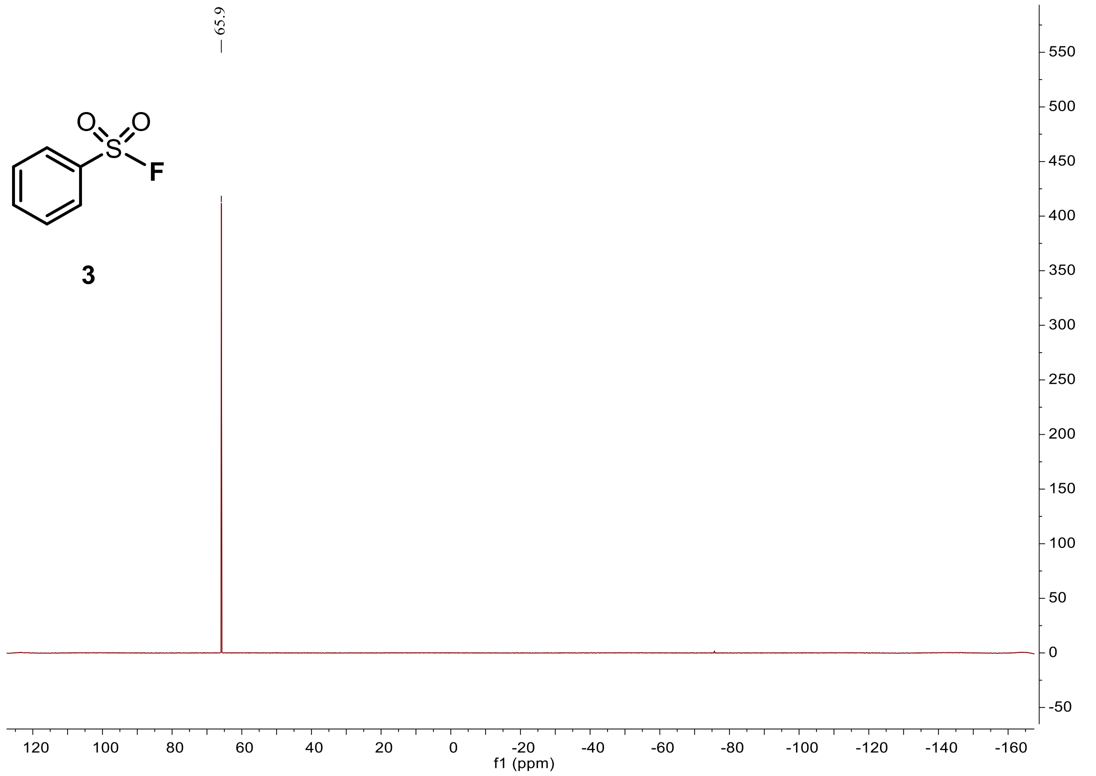

Supplementary Figure 17. ^{19}F-NMR (282 MHz, $CDCl_3$) spectrum of **3**

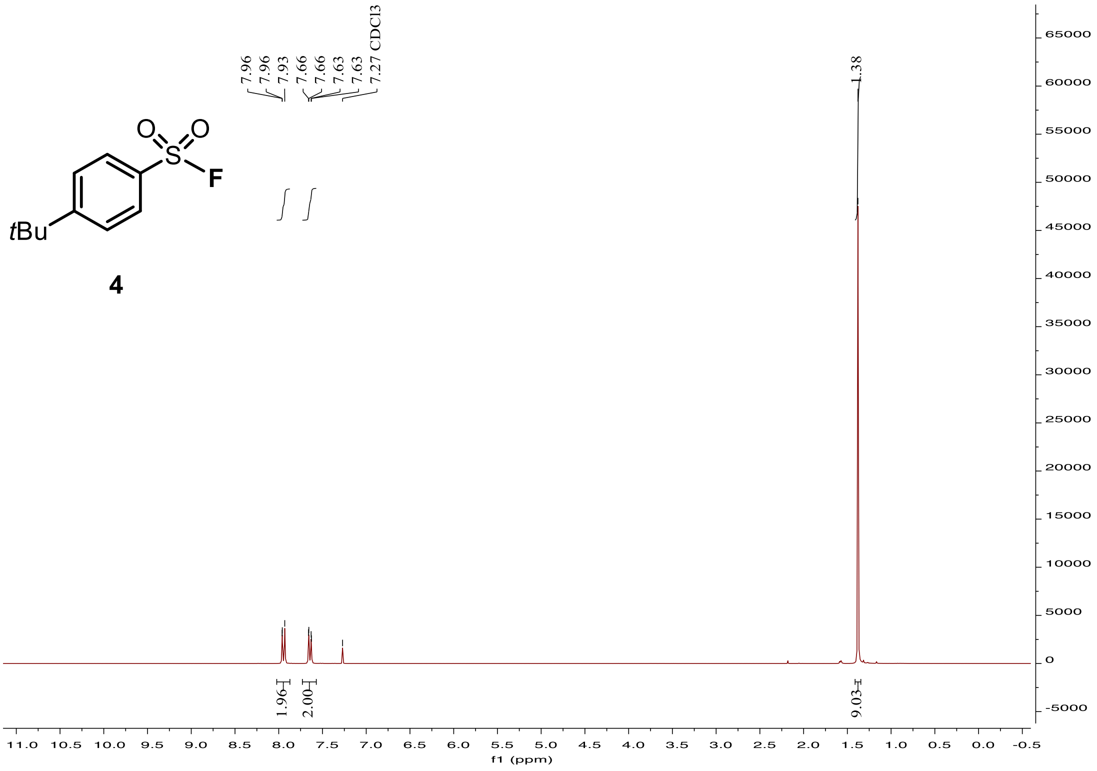

Supplementary Figure 18. ^{1}H-NMR (300 MHz, $CDCl_3$) spectrum of **4**

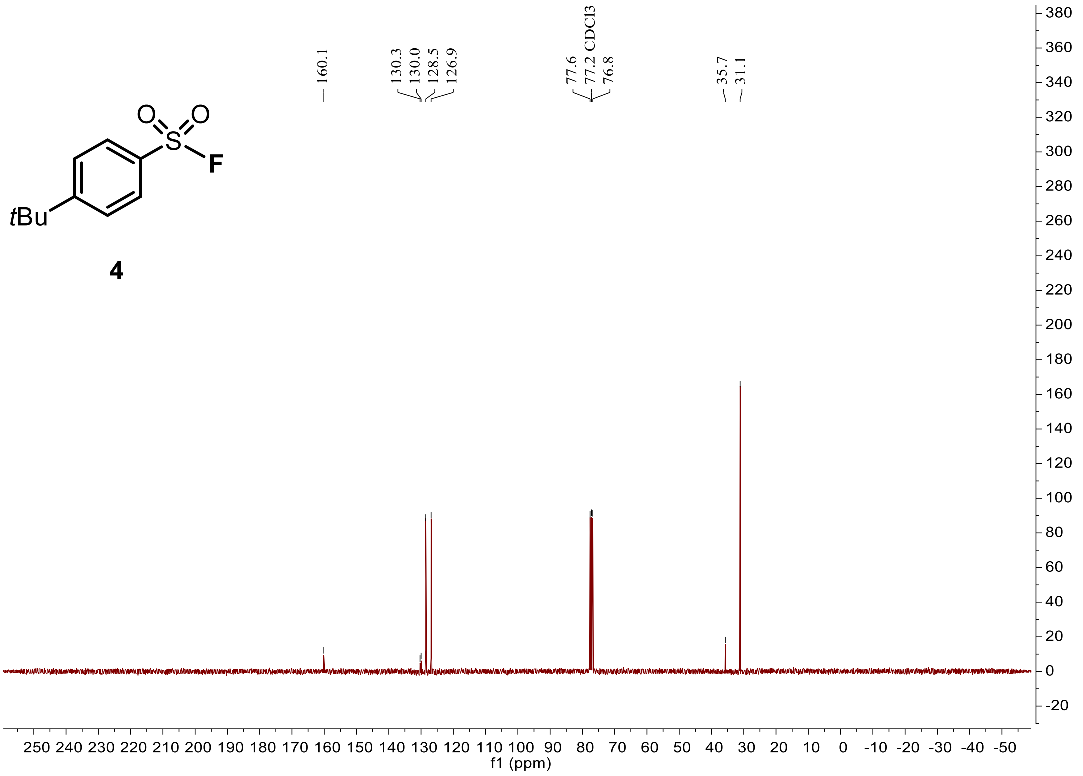

Supplementary Figure 19. ^{13}C-NMR (75 MHz, $CDCl_3$) spectrum of **4**

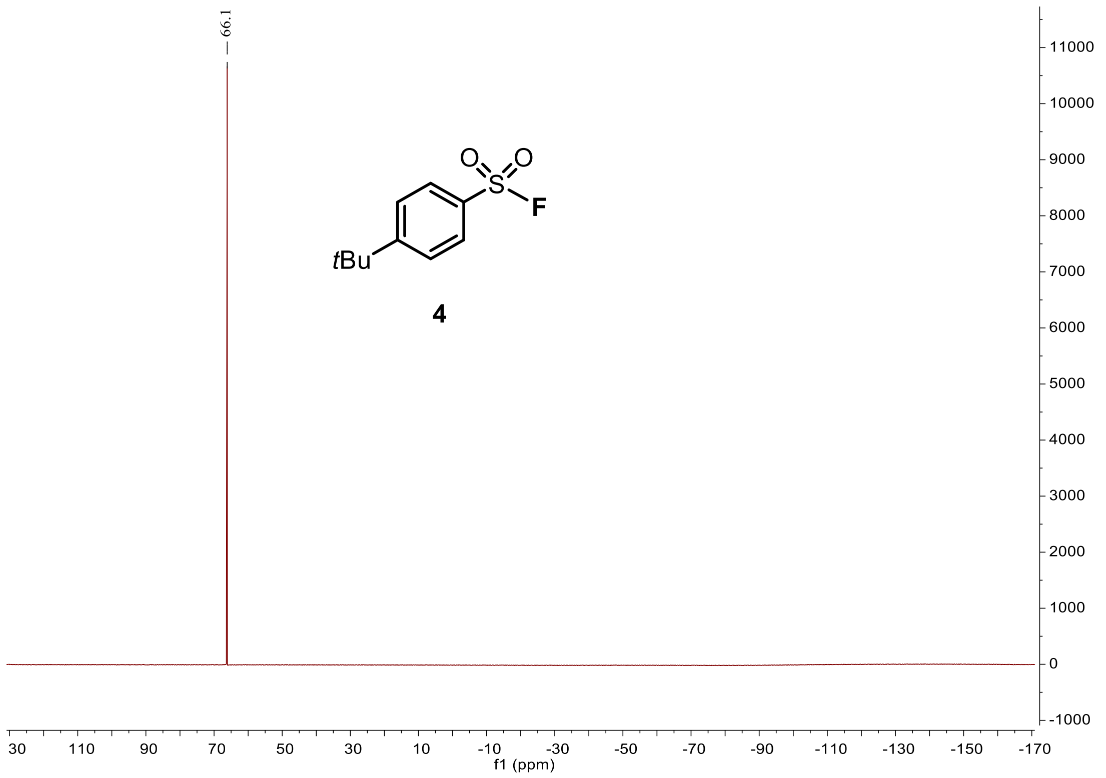

Supplementary Figure 20. ^{19}F-NMR (282 MHz, $CDCl_3$) spectrum of **4**

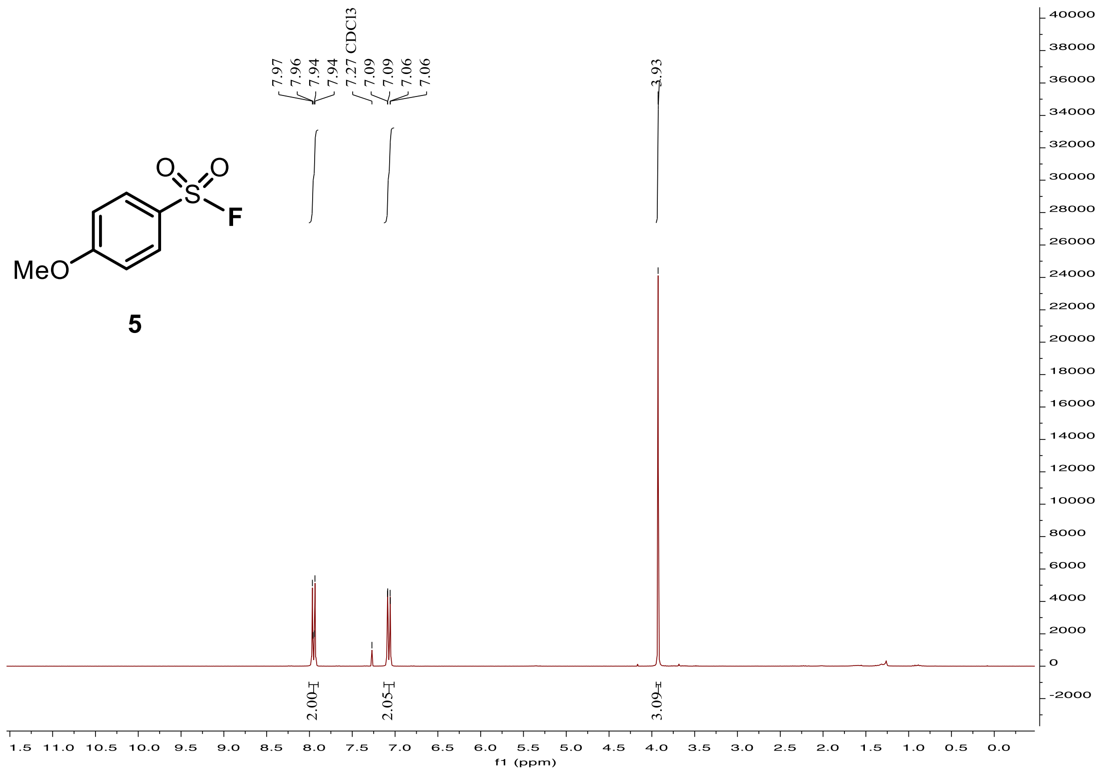

Supplementary Figure 21. ^{1}H-NMR (300 MHz, $CDCl_3$) spectrum of **5**

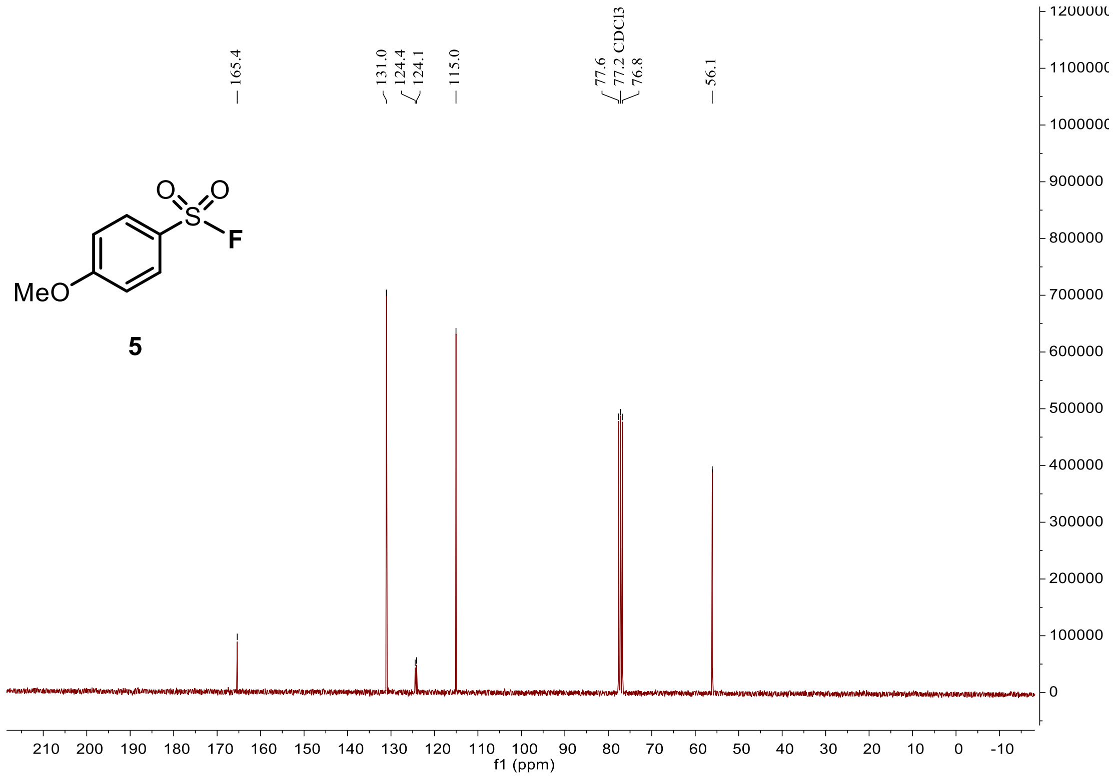

Supplementary Figure 22. ^{13}C-NMR (75 MHz, $CDCl_3$) spectrum of **5**

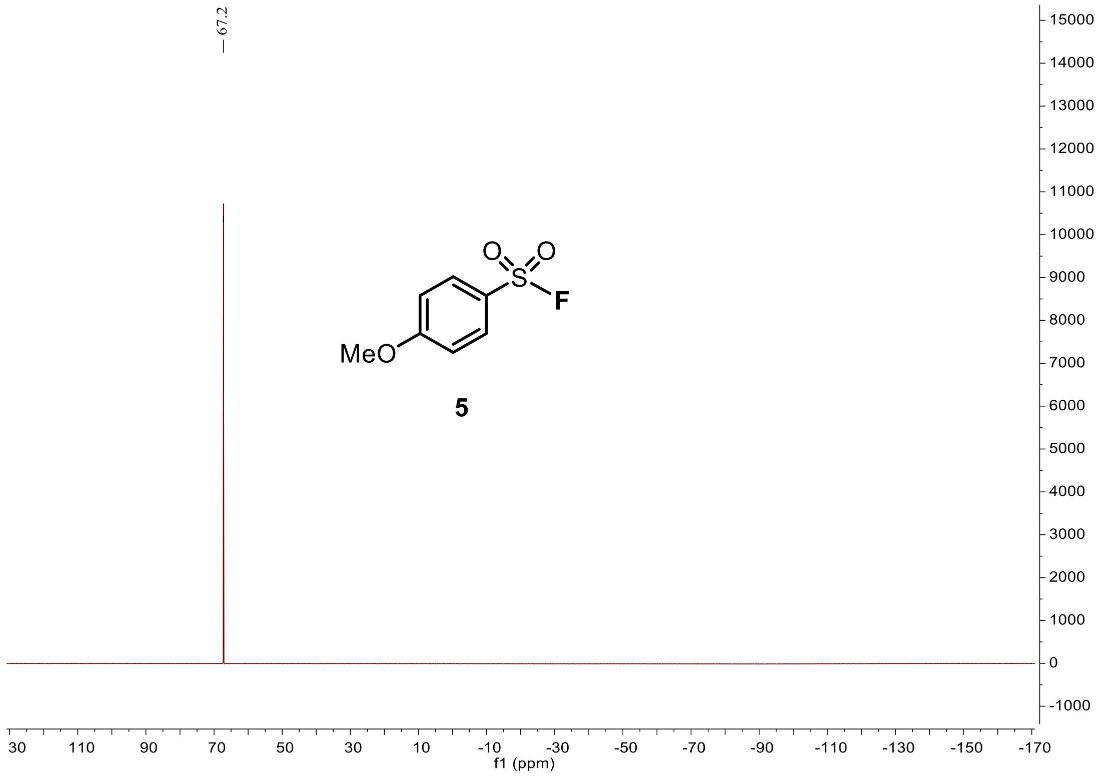

Supplementary Figure 23. ^{19}F-NMR (282 MHz, $CDCl_3$) spectrum of **5**

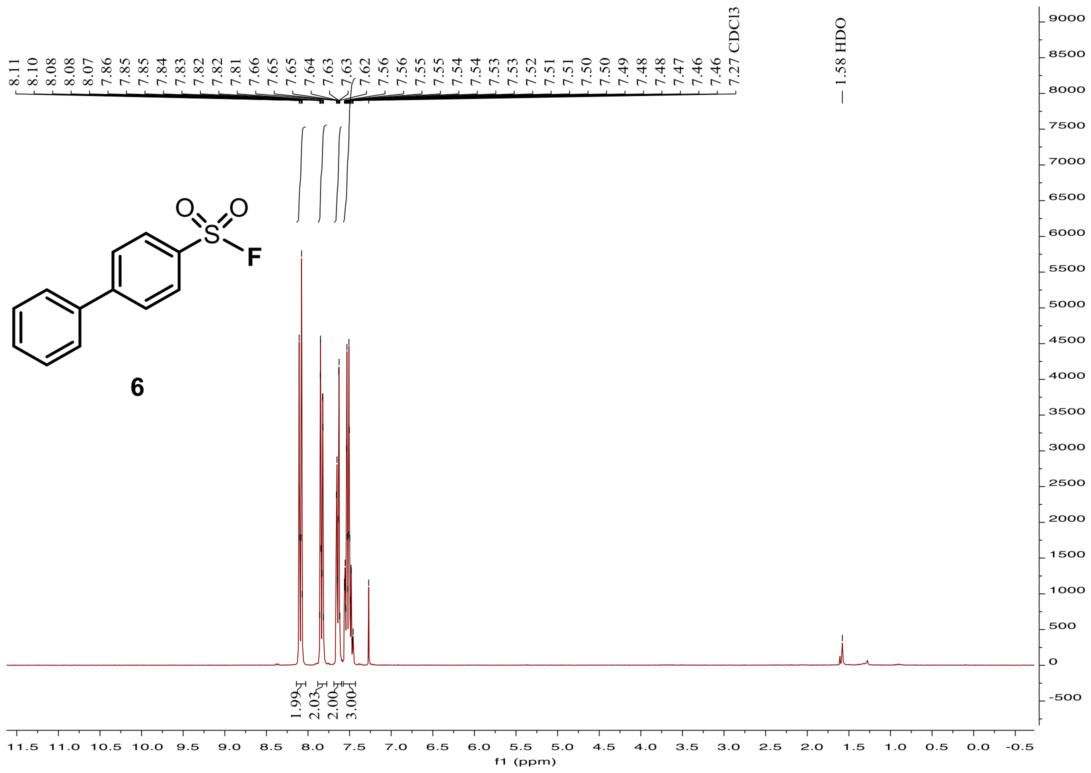

Supplementary Figure 24. ^{1}H-NMR (300 MHz, $CDCl_3$) spectrum of **6**

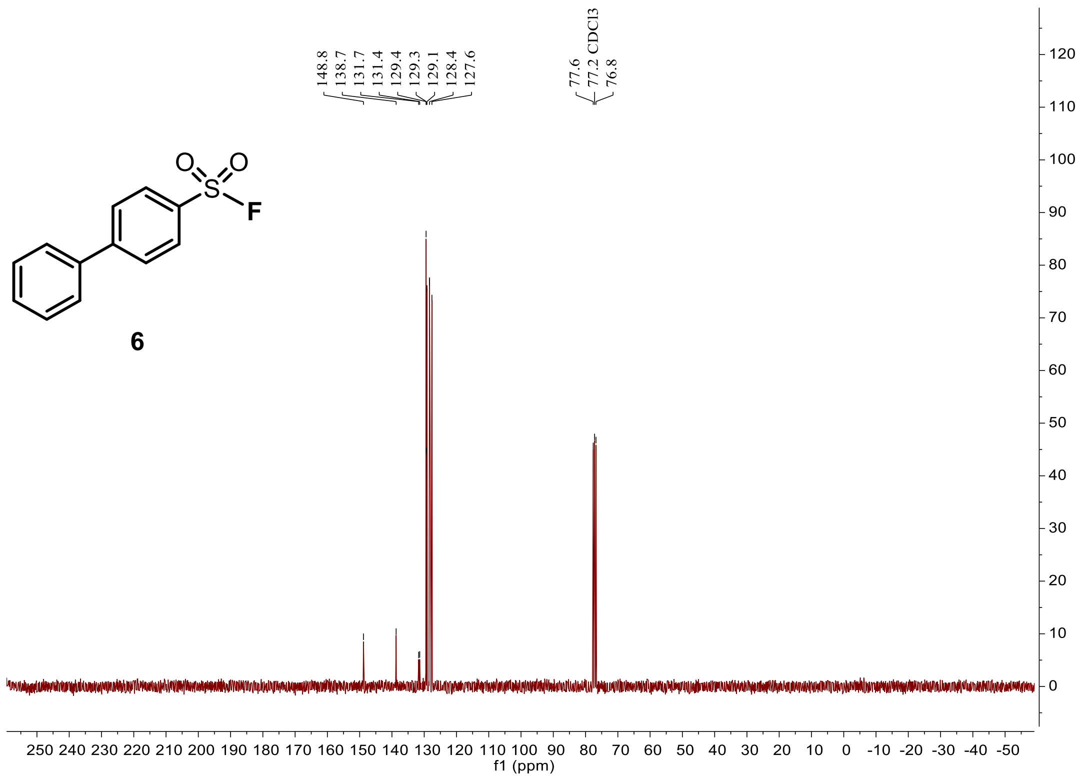

Supplementary Figure 25. ^{13}C-NMR (75 MHz, $CDCl_3$) spectrum of **6**

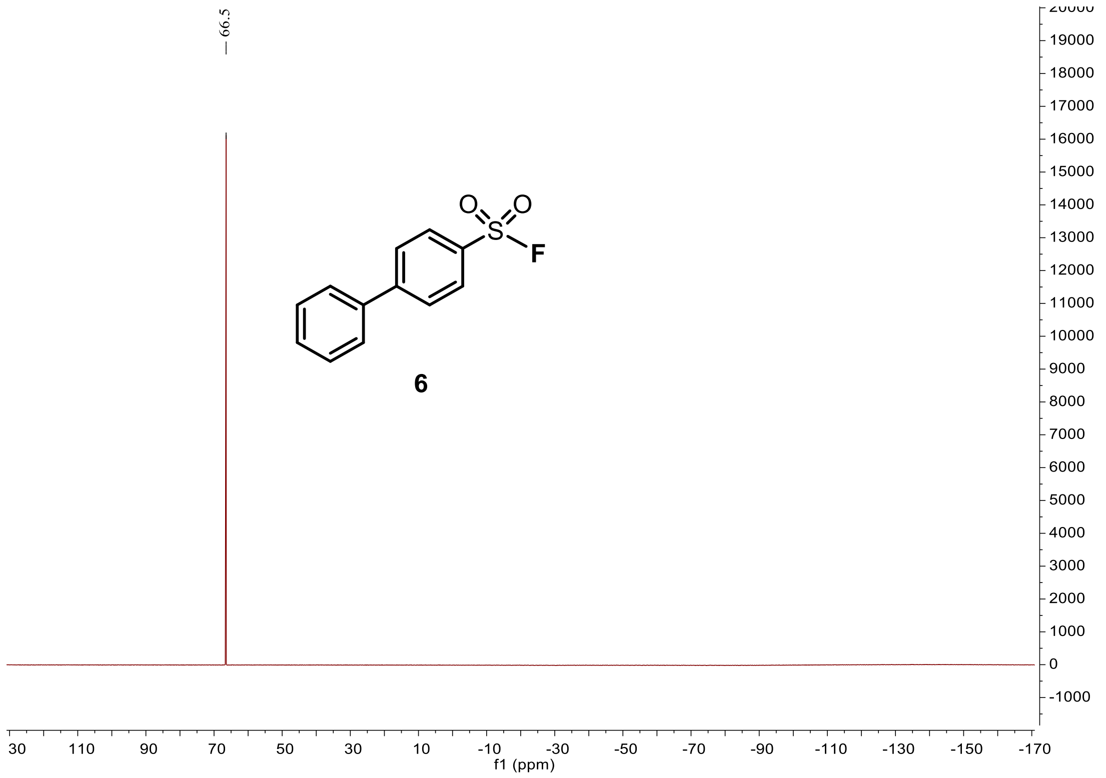

Supplementary Figure 26. ^{19}F-NMR (282 MHz, $CDCl_3$) spectrum of **6**

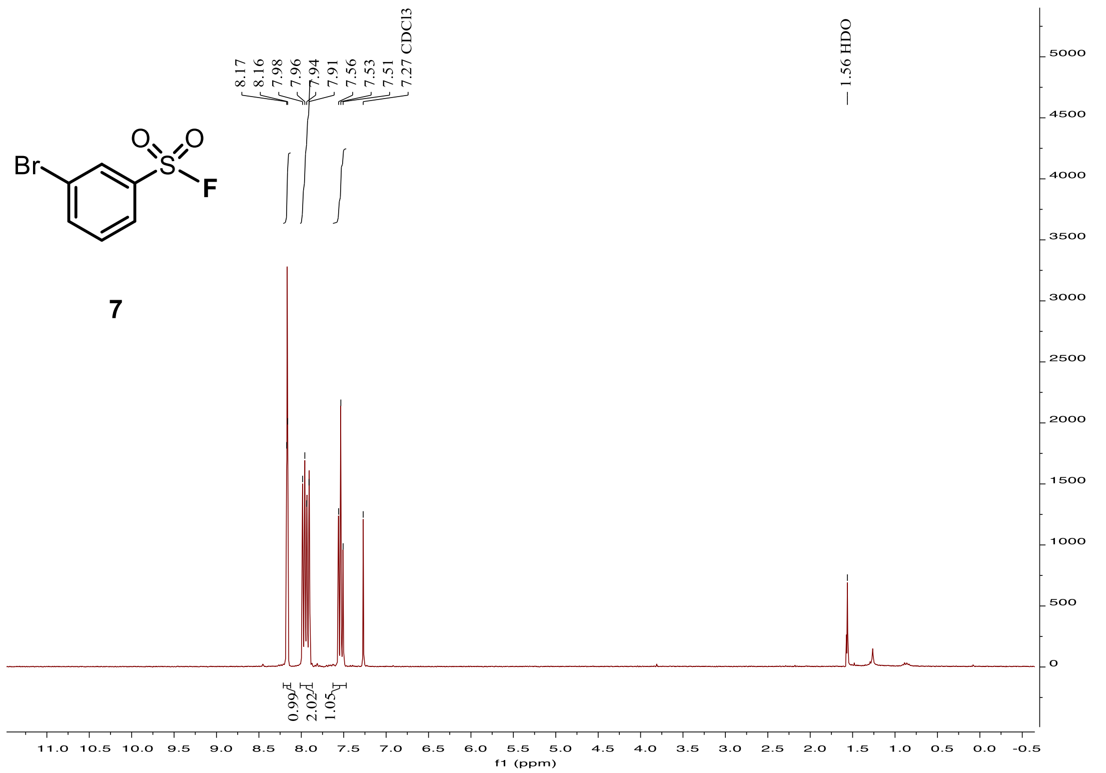

Supplementary Figure 27. ^{1}H-NMR (300 MHz, $CDCl_3$) spectrum of **7**

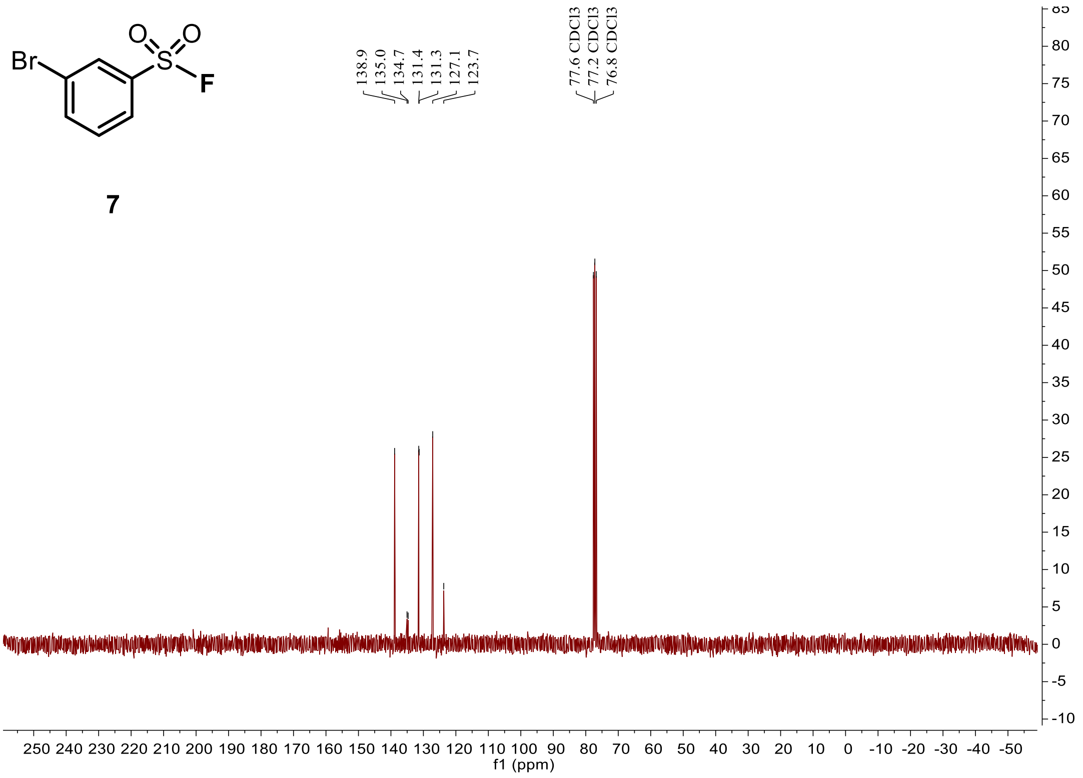

Supplementary Figure 28. ^{13}C-NMR (75 MHz, $CDCl_3$) spectrum of **7**

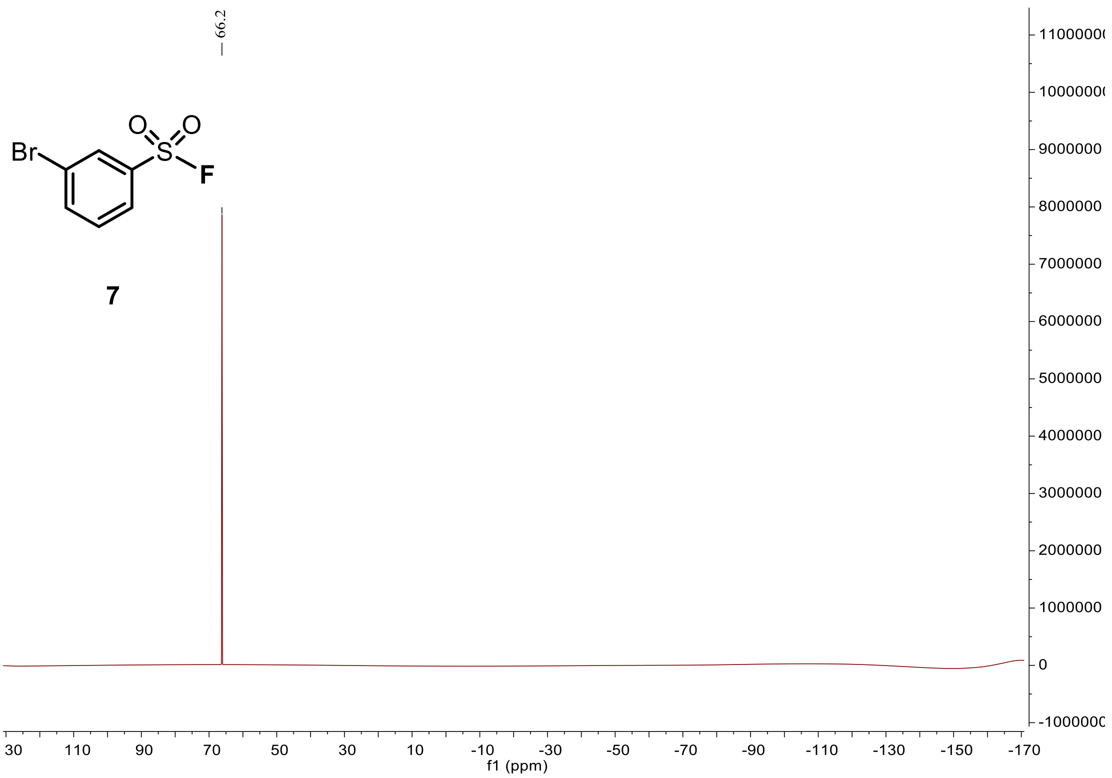

Supplementary Figure 29. ^{19}F-NMR (282 MHz, $CDCl_3$) spectrum of **7**

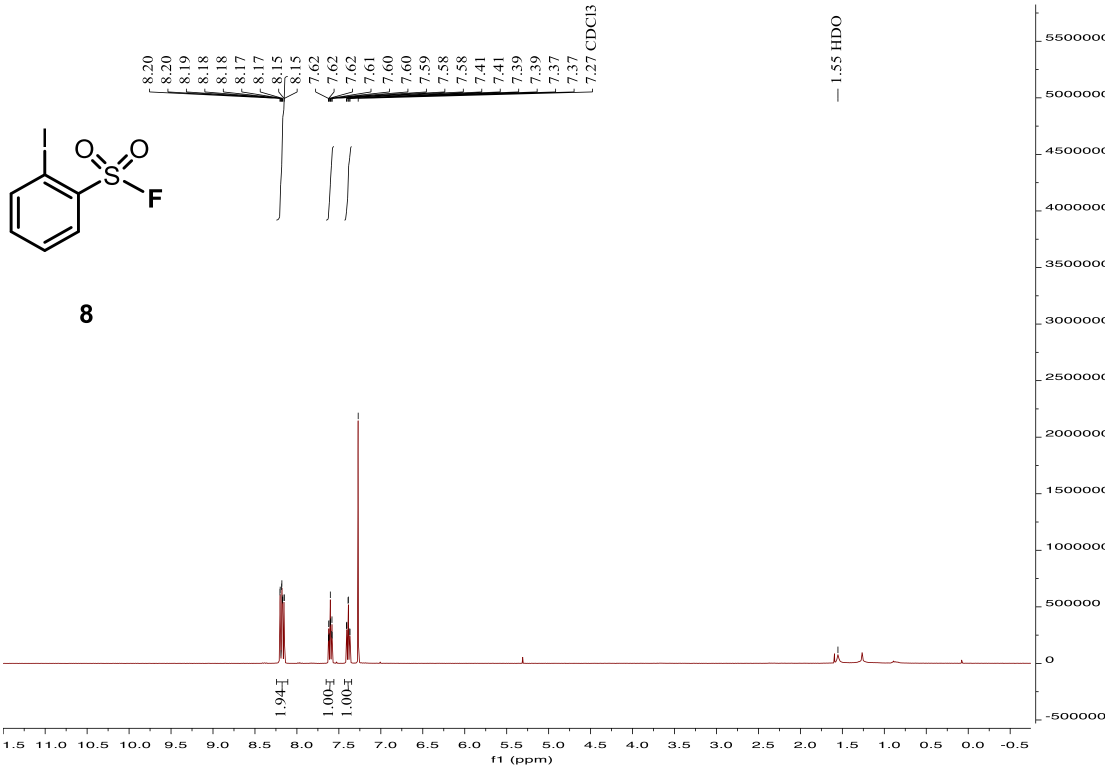

Supplementary Figure 30. ^{1}H-NMR (300 MHz, $CDCl_3$) spectrum of **8**

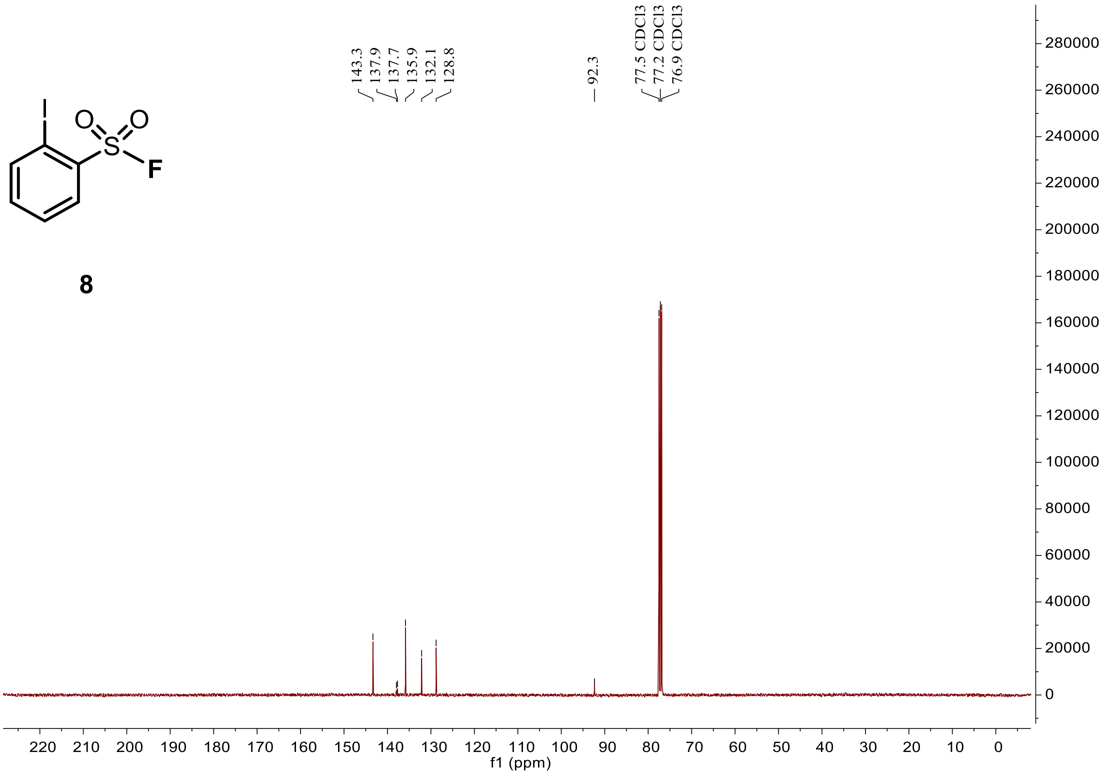

Supplementary Figure 31. ^{13}C-NMR (75 MHz, $CDCl_3$) spectrum of **8**

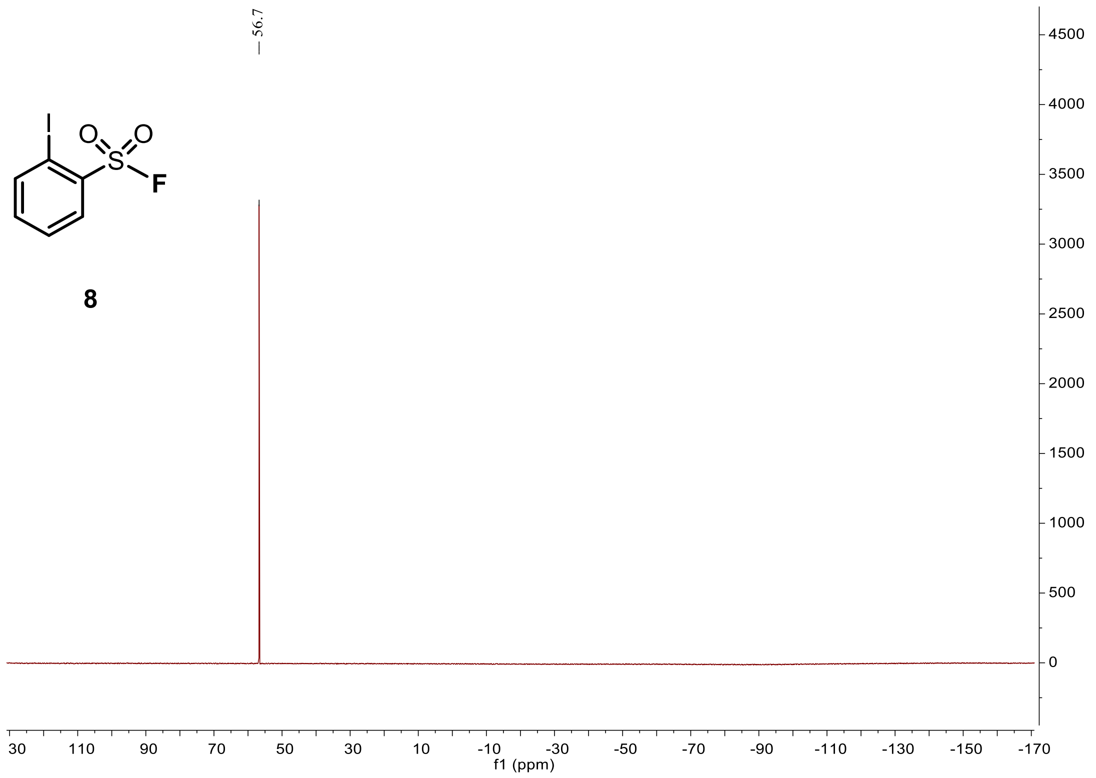

Supplementary Figure 32. ^{19}F-NMR (282 MHz, $CDCl_3$) spectrum of **8**

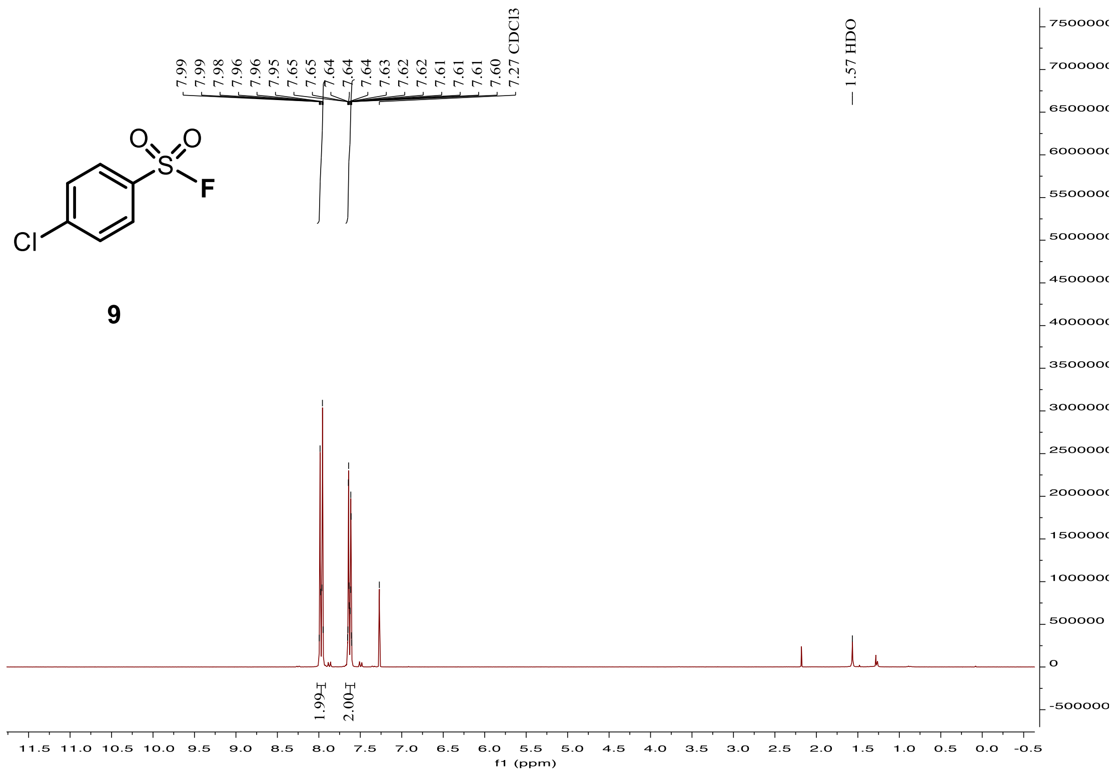

Supplementary Figure 33. ^{1}H-NMR (300 MHz, $CDCl_3$) spectrum of **9**

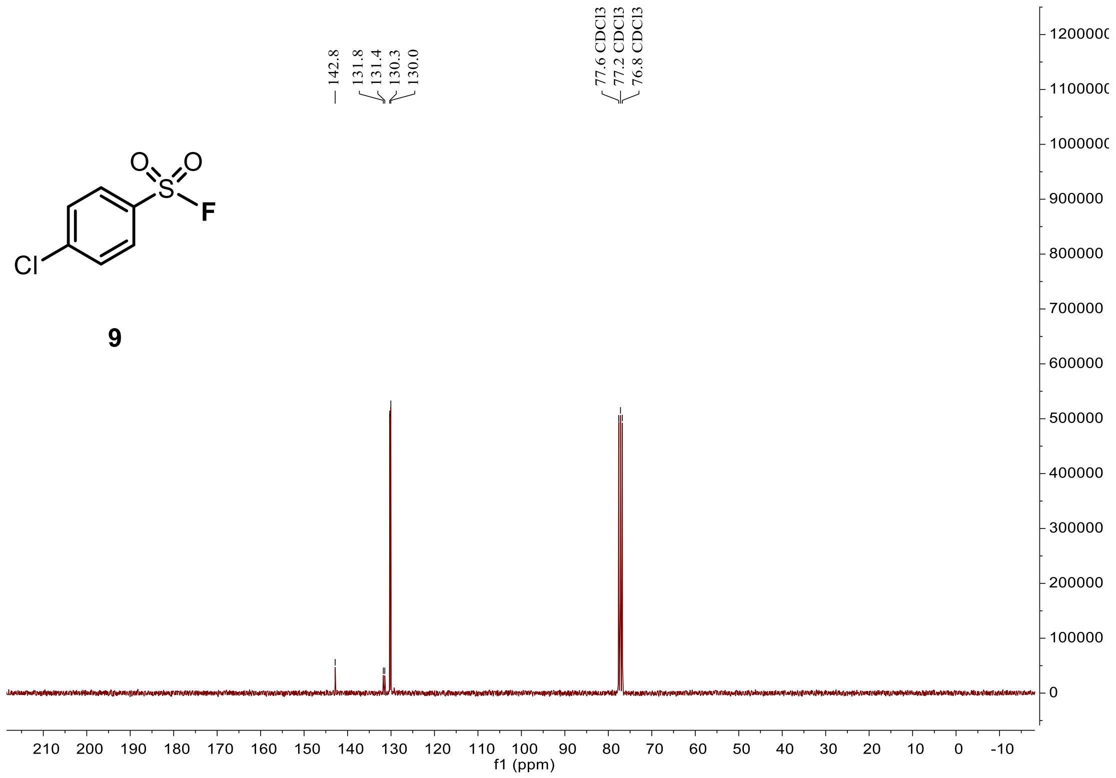

Supplementary Figure 34. ^{13}C-NMR (75 MHz, $CDCl_3$) spectrum of **9**

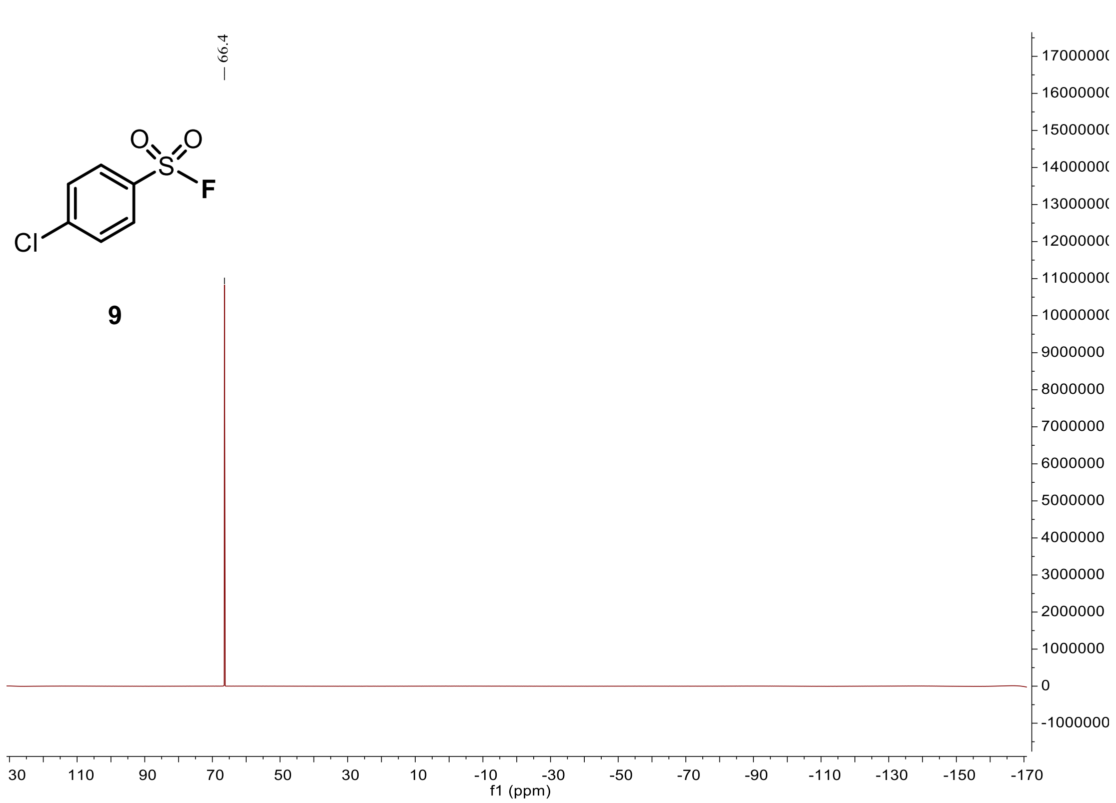

Supplementary Figure 35. ^{19}F-NMR (282 MHz, $CDCl_3$) spectrum of **9**

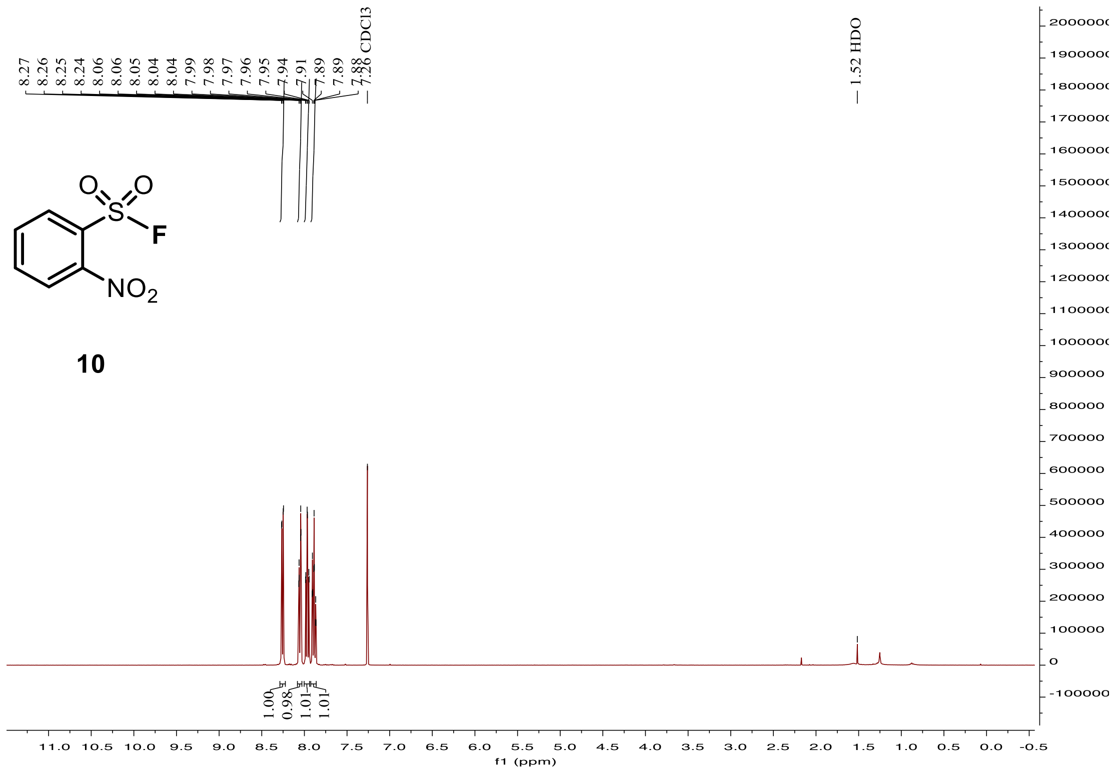

Supplementary Figure 36. ^{1}H-NMR (300 MHz, $CDCl_3$) spectrum of **10**

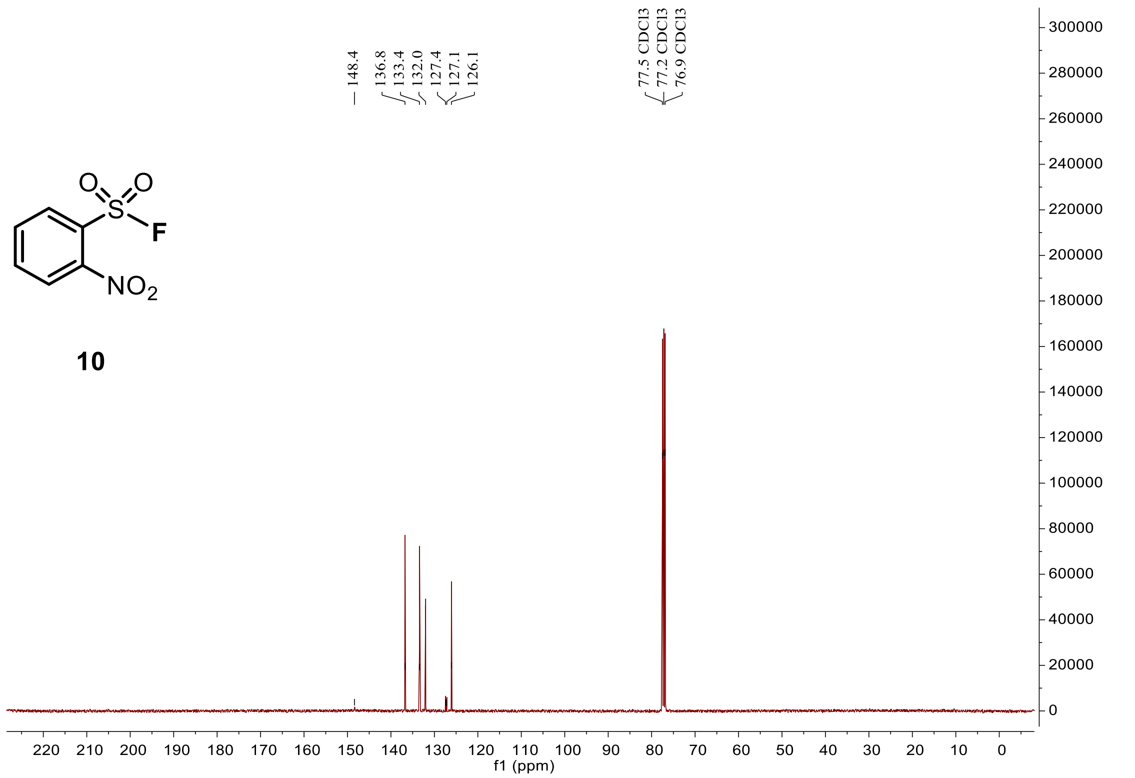

Supplementary Figure 37. ^{13}C-NMR (75 MHz, $CDCl_3$) spectrum of **10**

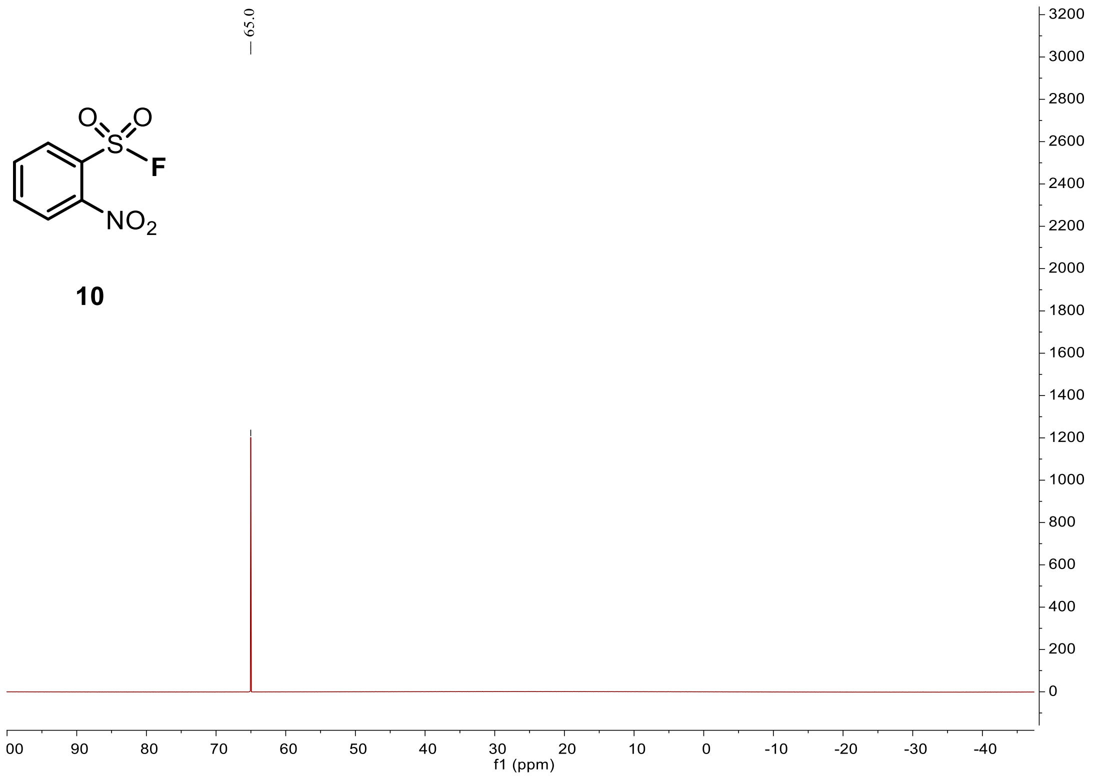

Supplementary Figure 38. ^{19}F-NMR (282 MHz, $CDCl_3$) spectrum of **10**

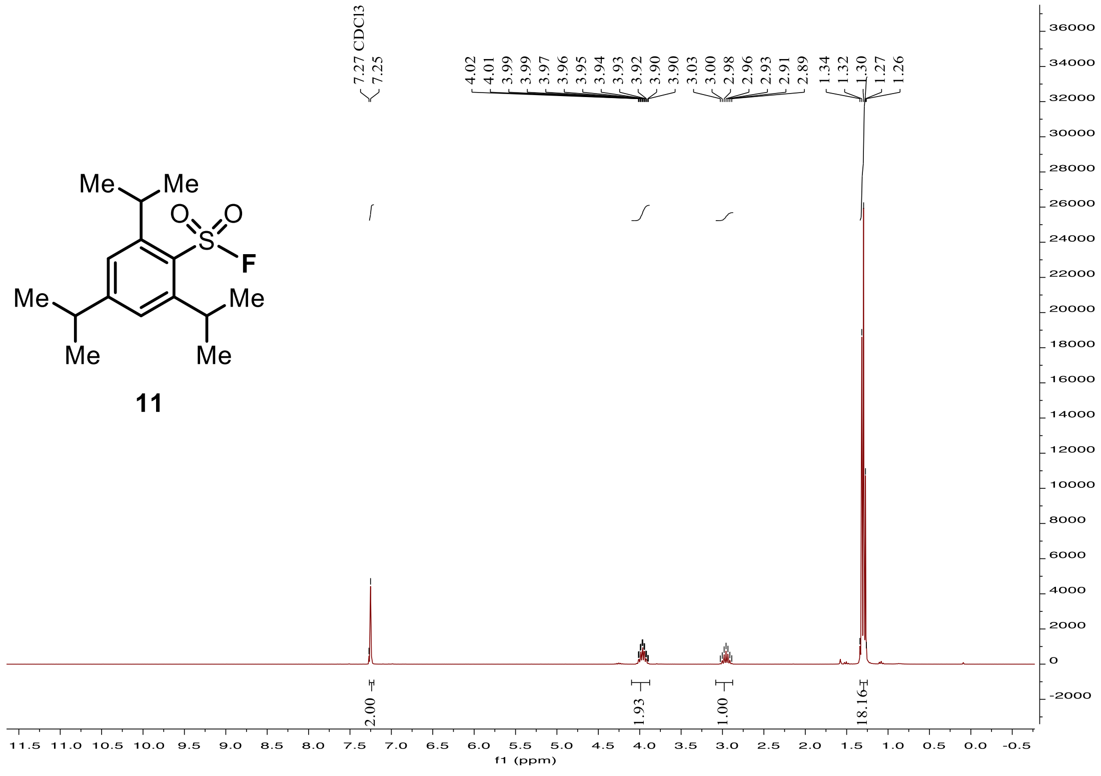

Supplementary Figure 39. ^{1}H-NMR (300 MHz, $CDCl_3$) spectrum of **11**

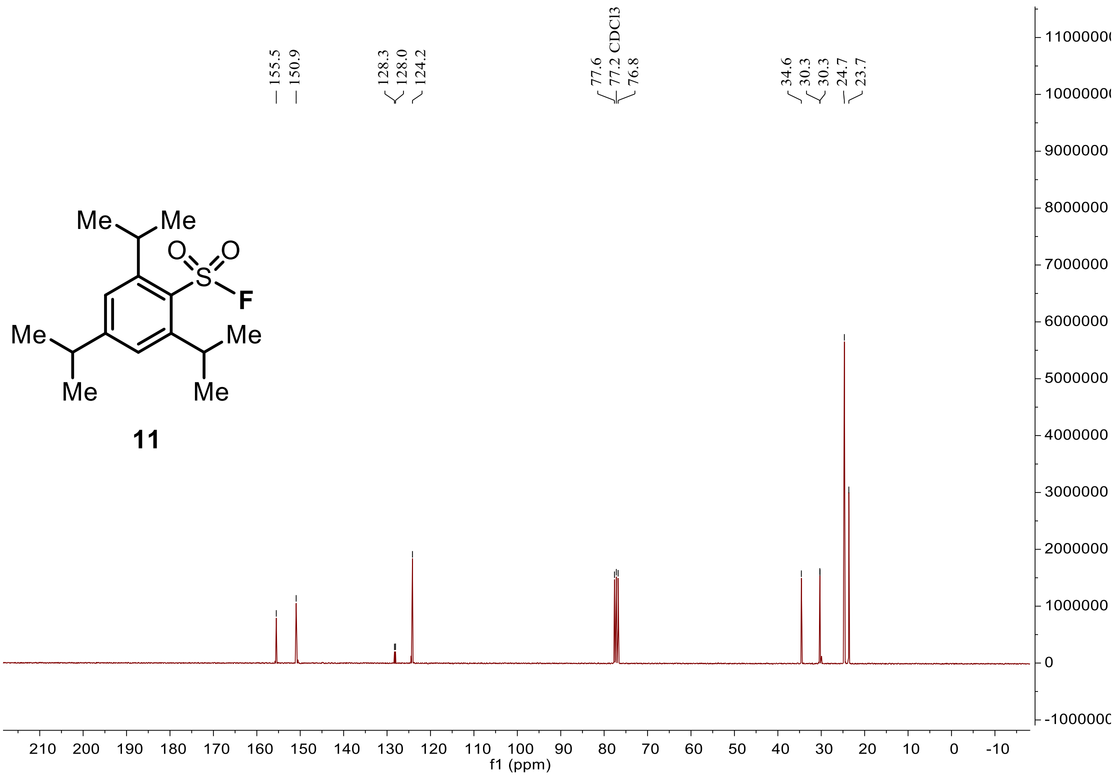

Supplementary Figure 40. ^{13}C-NMR (75 MHz, $CDCl_3$) spectrum of **11**

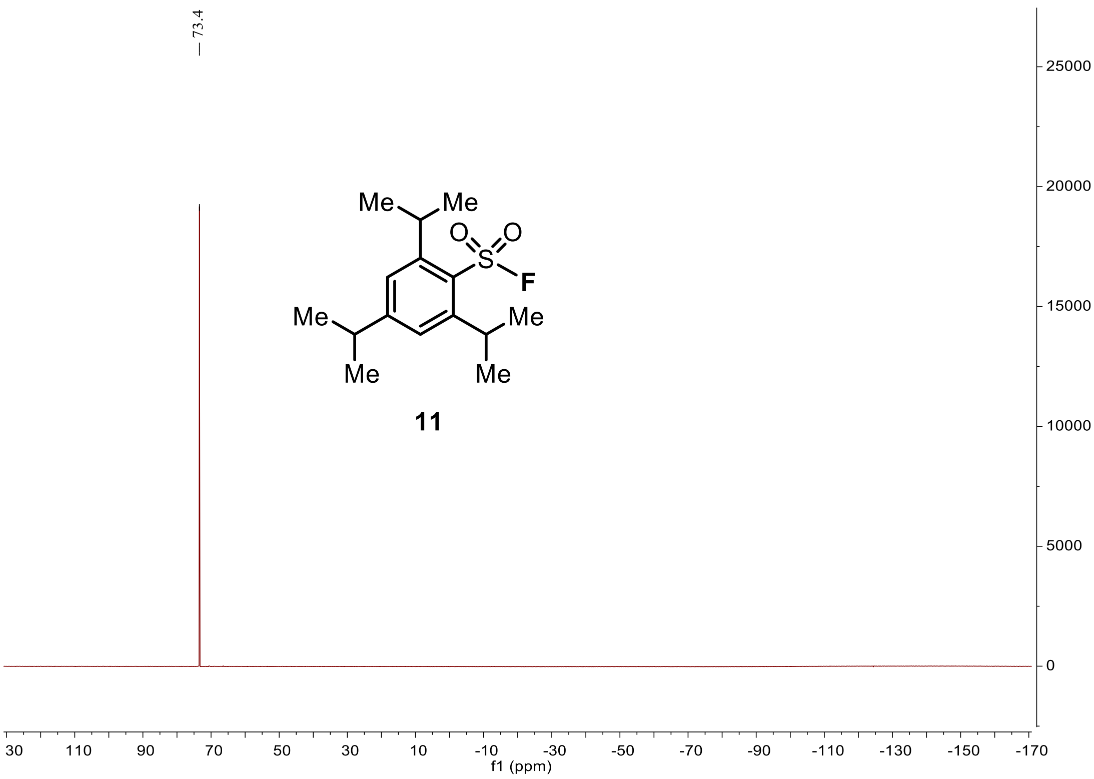

Supplementary Figure 41. ^{19}F-NMR (282 MHz, $CDCl_3$) spectrum of **11**

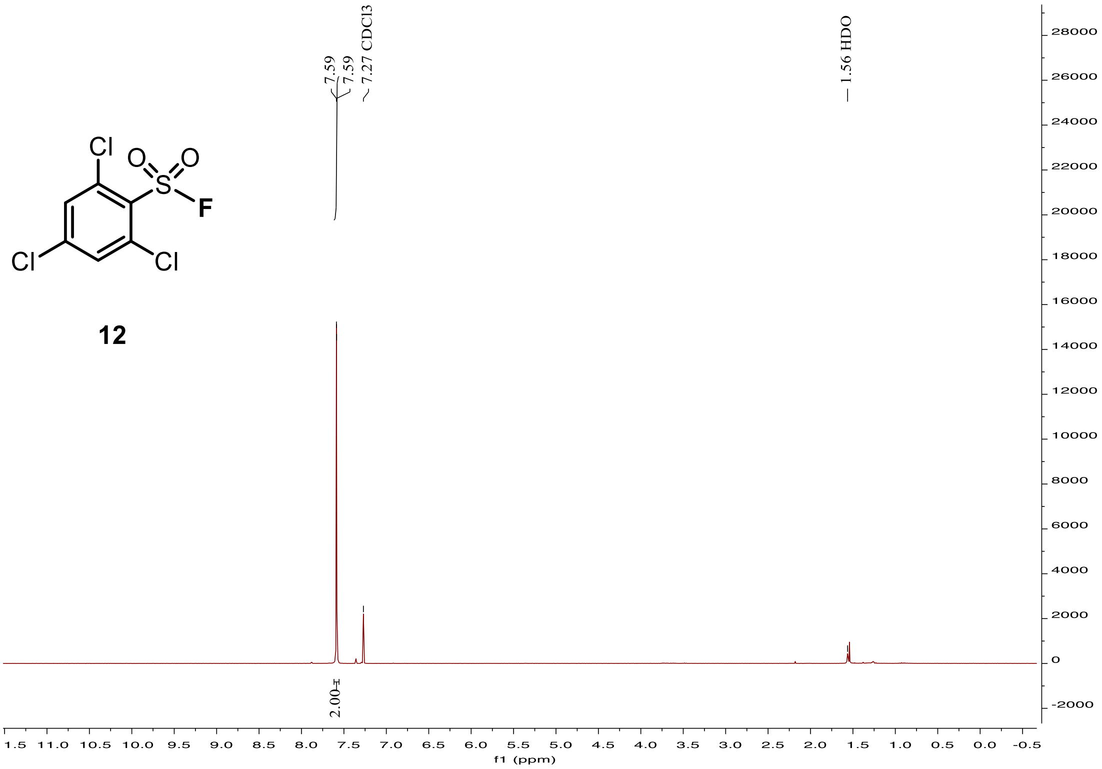

Supplementary Figure 42. ^{1}H-NMR (300 MHz, $CDCl_3$) spectrum of **12**

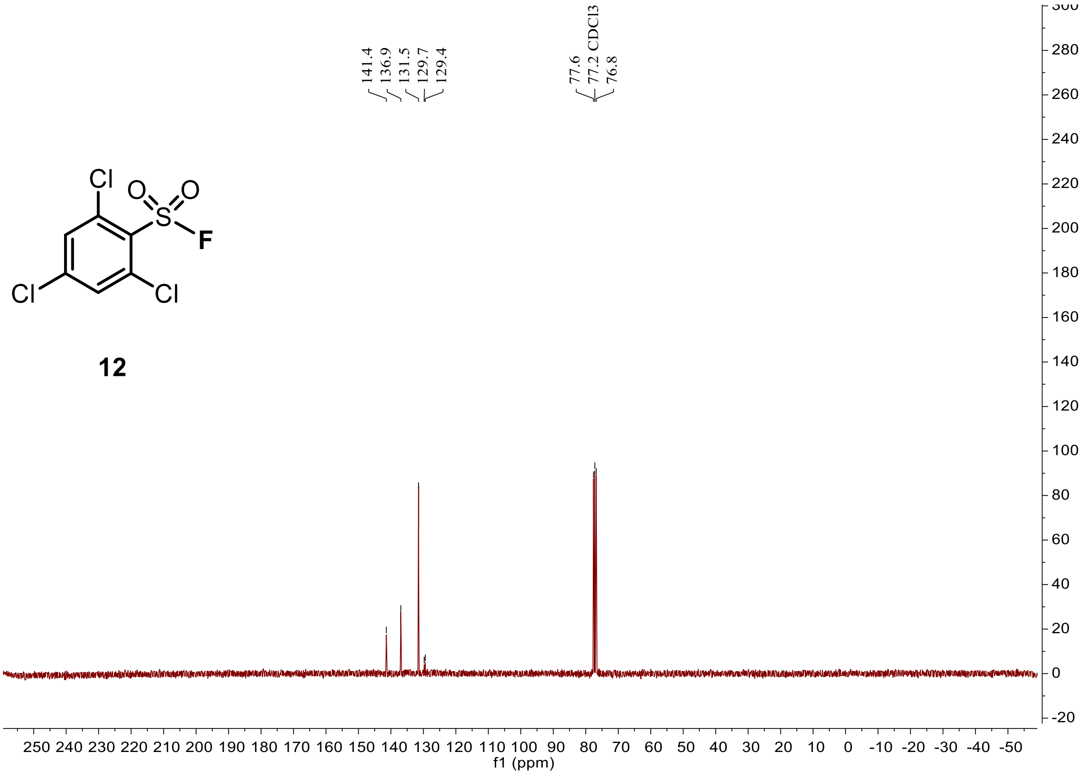

Supplementary Figure 43. ^{13}C-NMR (75 MHz, $CDCl_3$) spectrum of **12**

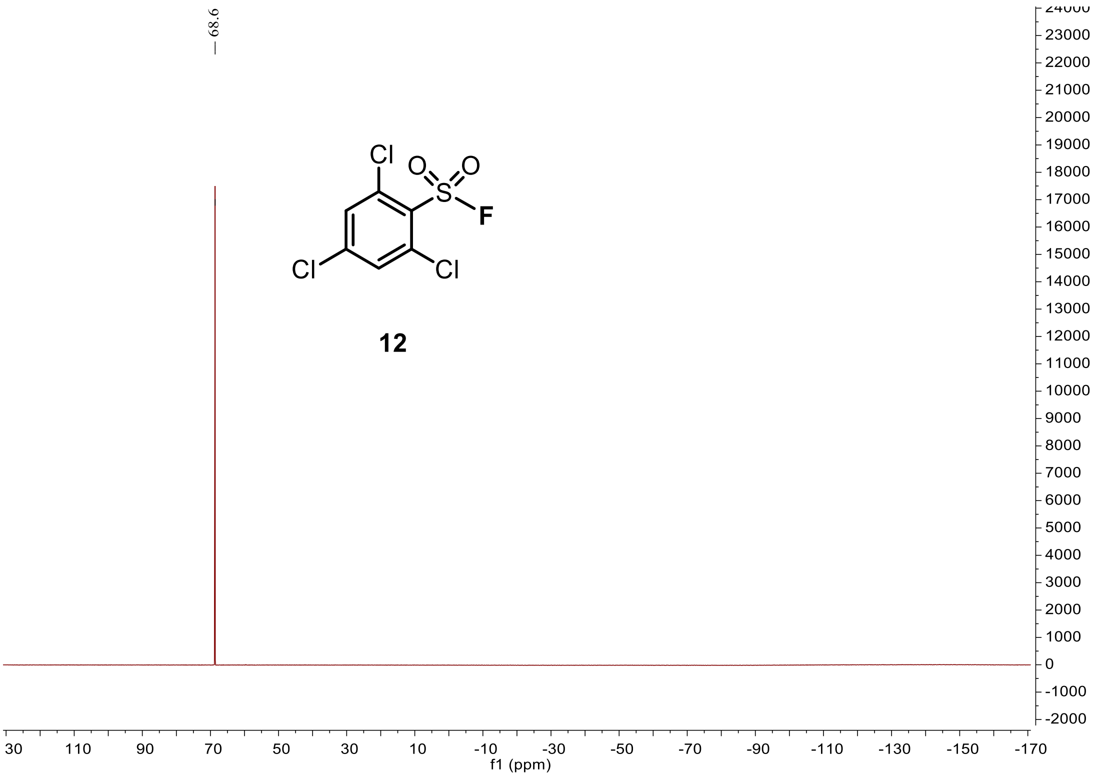

Supplementary Figure 44. ^{19}F-NMR (282 MHz, $CDCl_3$) spectrum of **12**

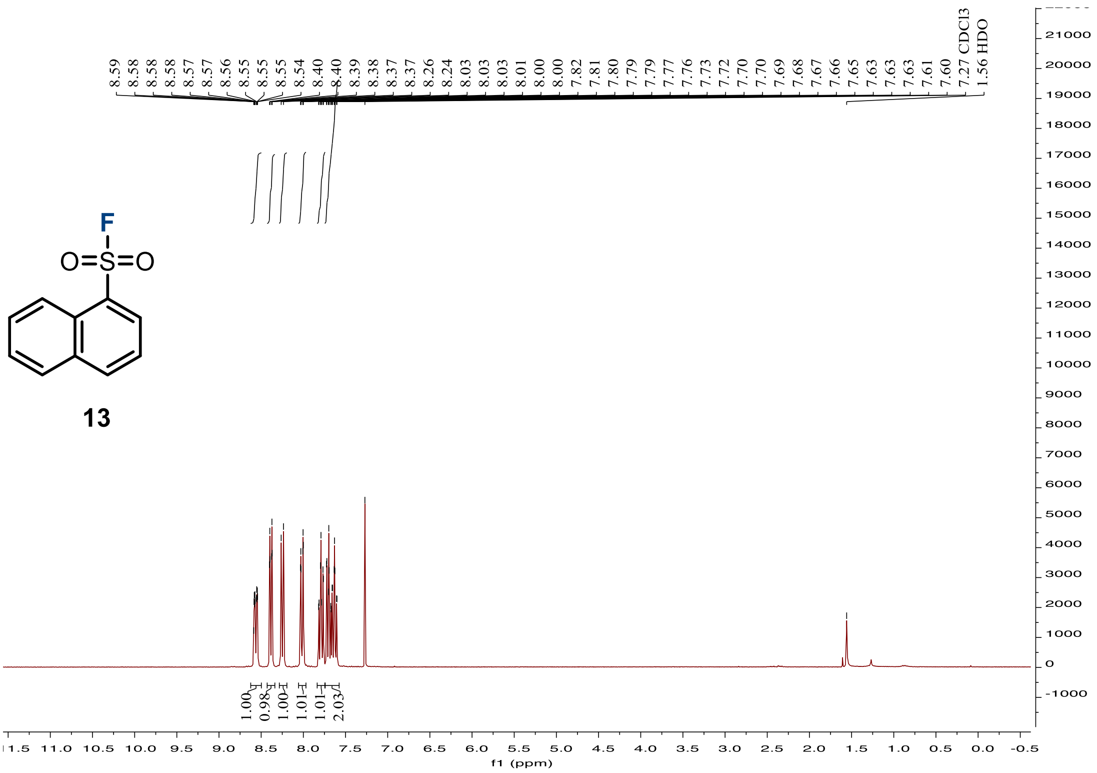

Supplementary Figure 45. ^{1}H-NMR (300 MHz, $CDCl_3$) spectrum of **13**

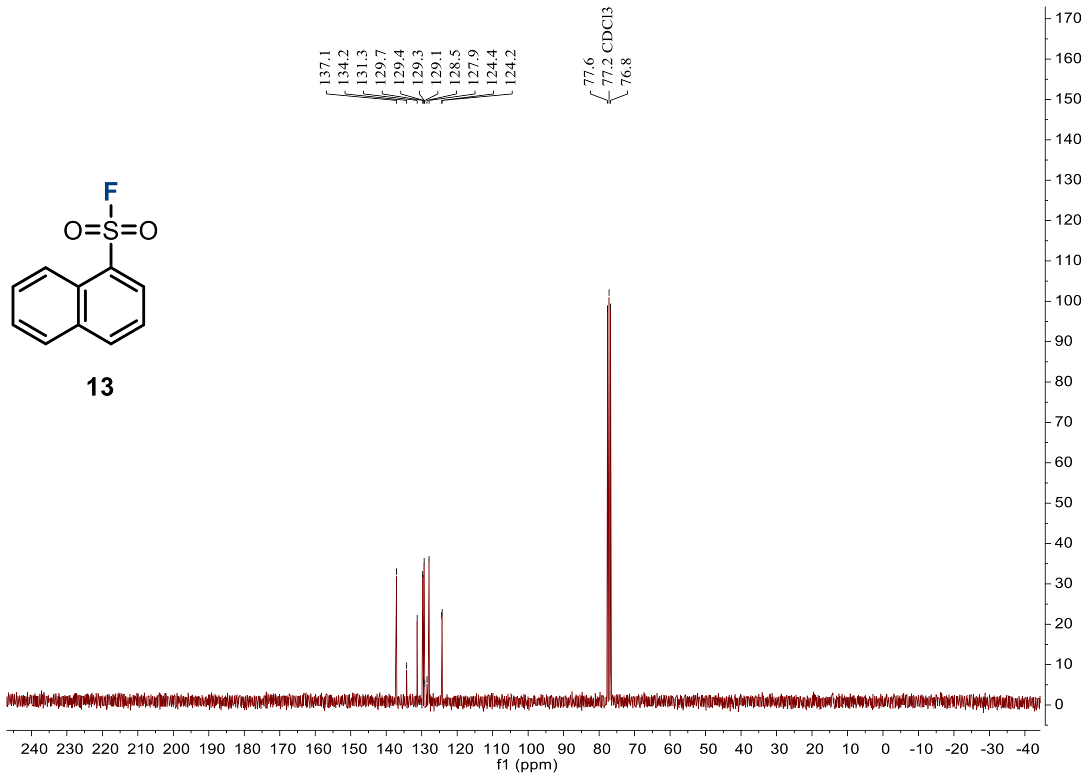

Supplementary Figure 46. ^{13}C-NMR (75 MHz, $CDCl_3$) spectrum of **13**

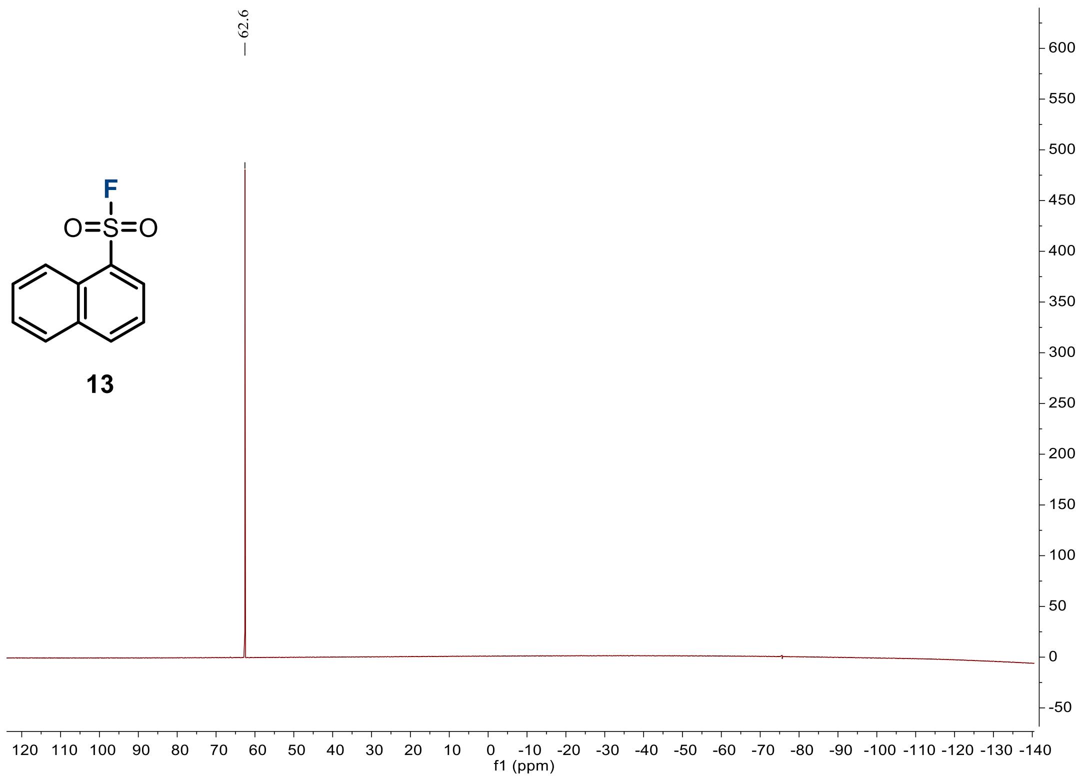

Supplementary Figure 47. ^{19}F-NMR (282 MHz, $CDCl_3$) spectrum of **13**

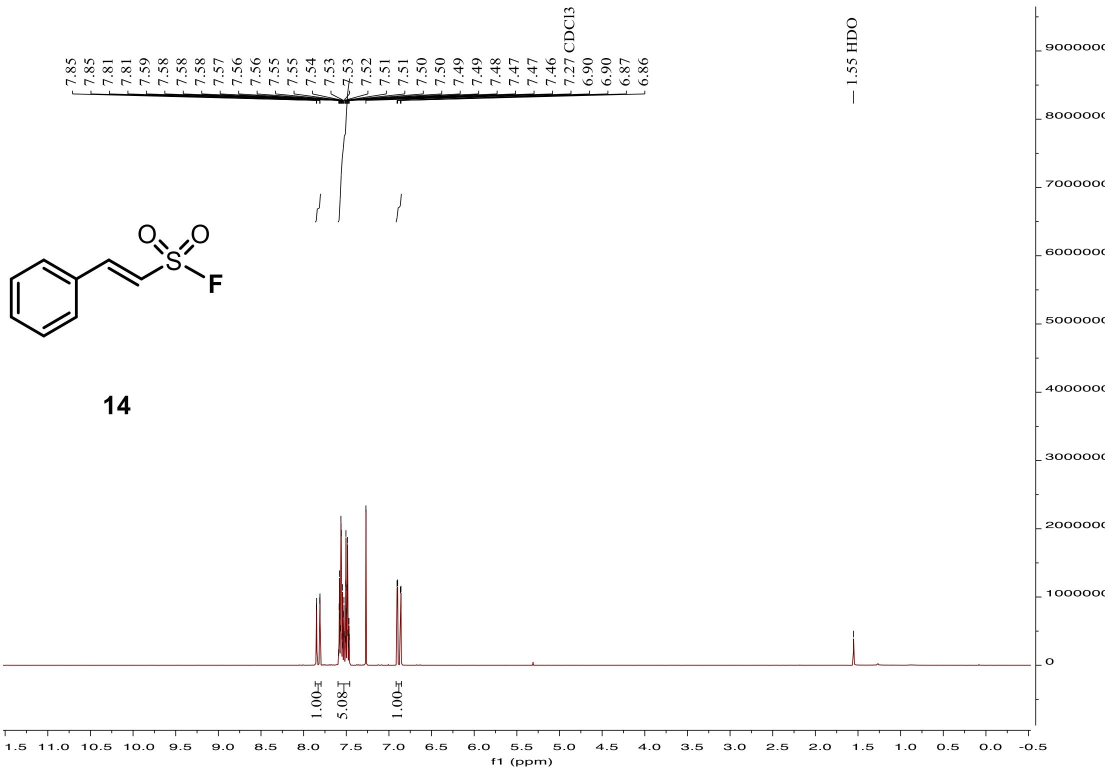

Supplementary Figure 48. ^{1}H-NMR (300 MHz, $CDCl_3$) spectrum of **14**

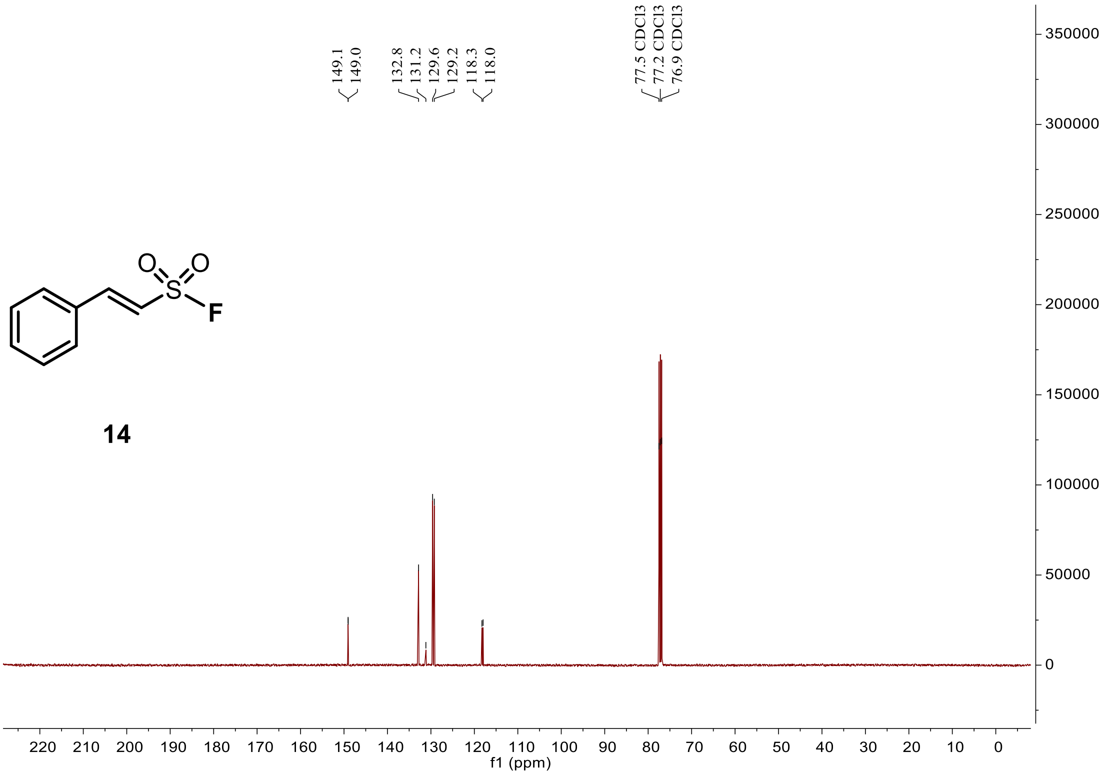

Supplementary Figure 49. ^{13}C-NMR (75 MHz, $CDCl_3$) spectrum of **14**

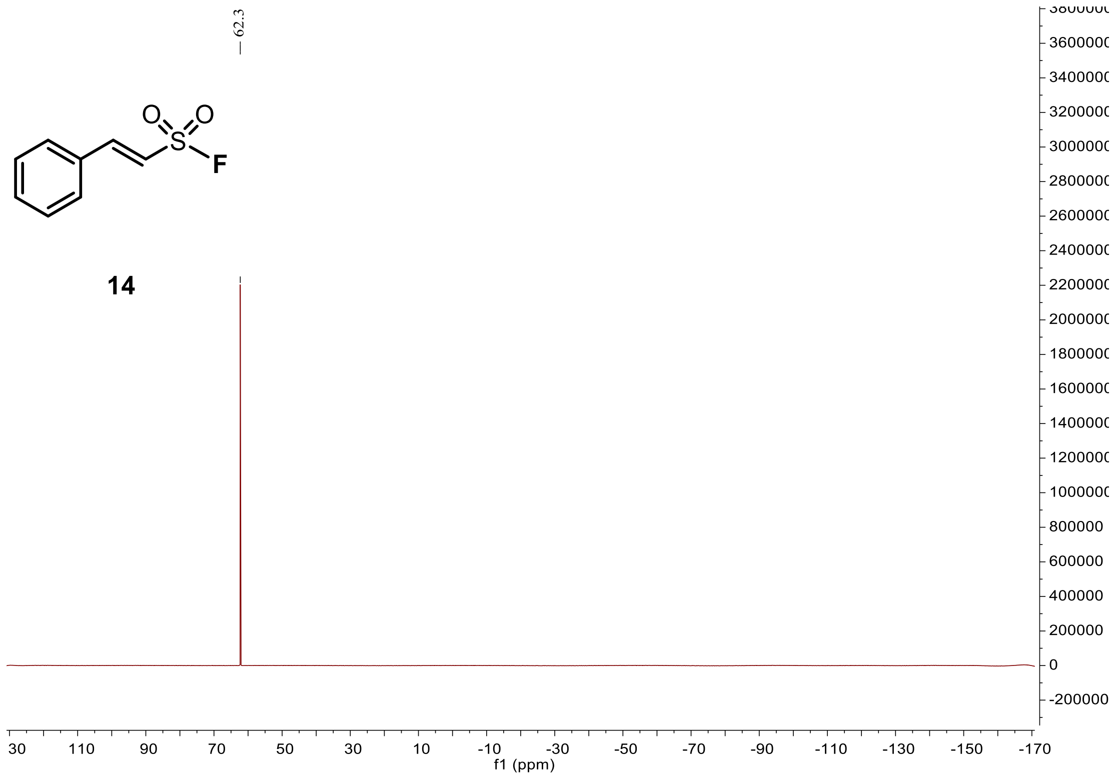

Supplementary Figure 50. ^{19}F-NMR (282 MHz, $CDCl_3$) spectrum of **14**

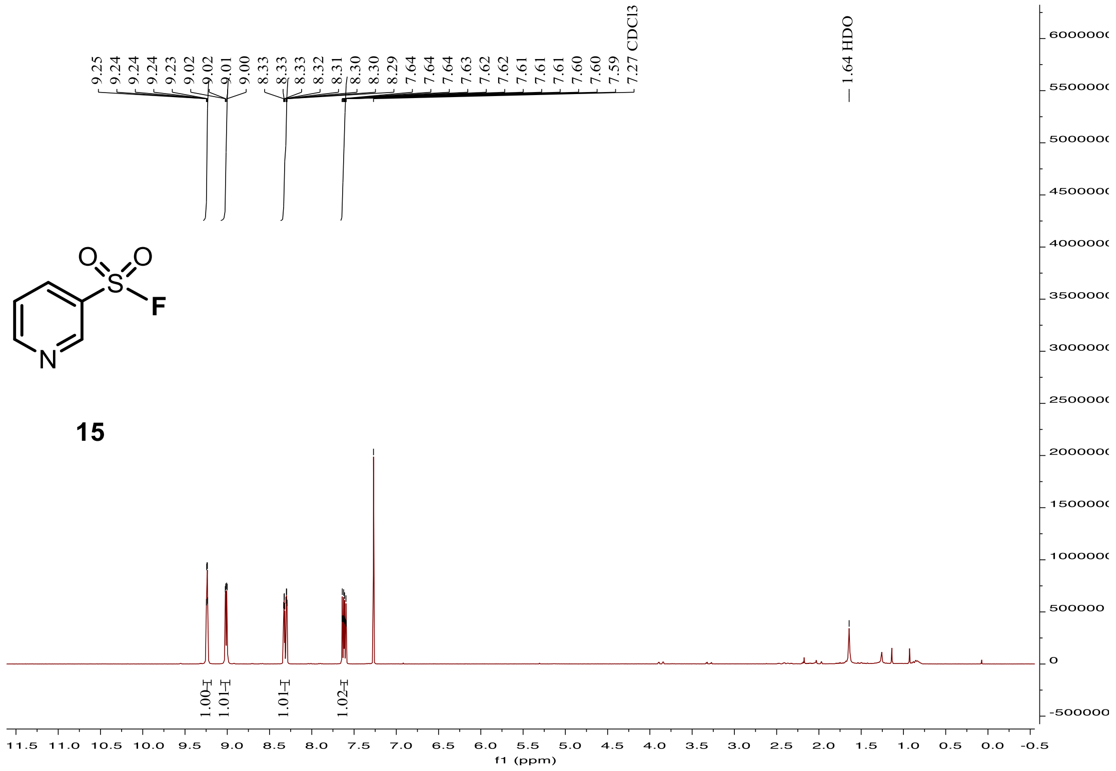

Supplementary Figure 51. ^{1}H-NMR (300 MHz, $CDCl_3$) spectrum of **15**

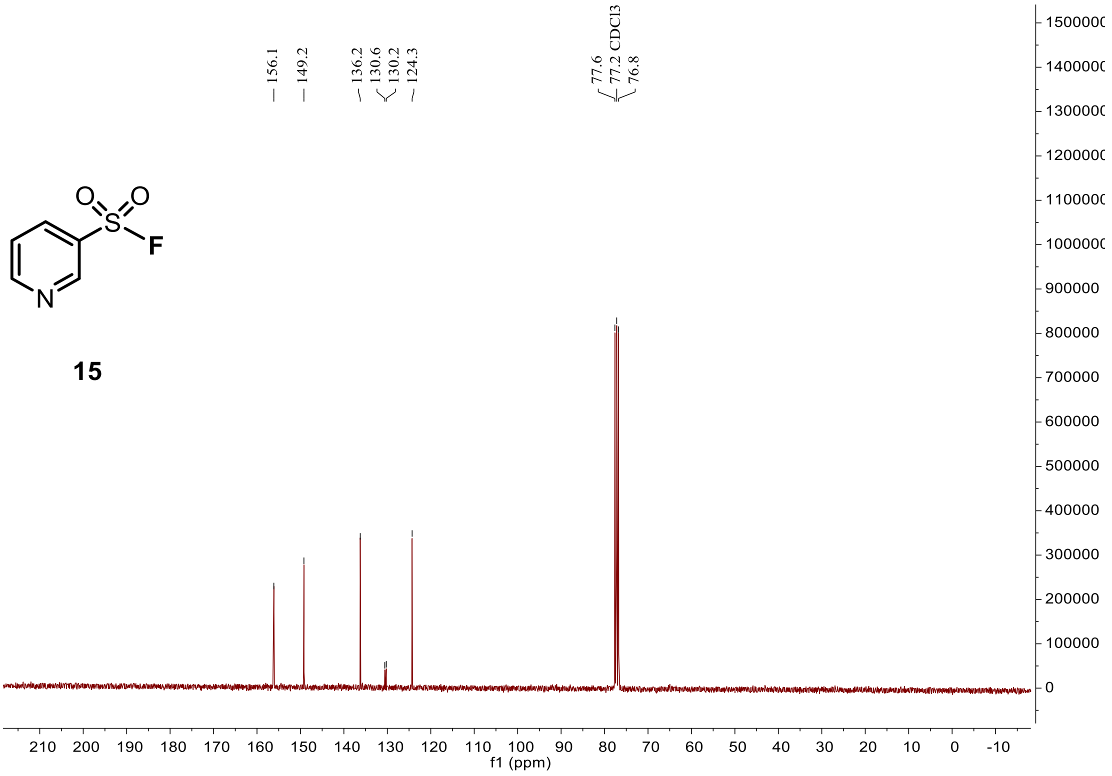

Supplementary Figure 52. ^{13}C-NMR (75 MHz, $CDCl_3$) spectrum of **15**

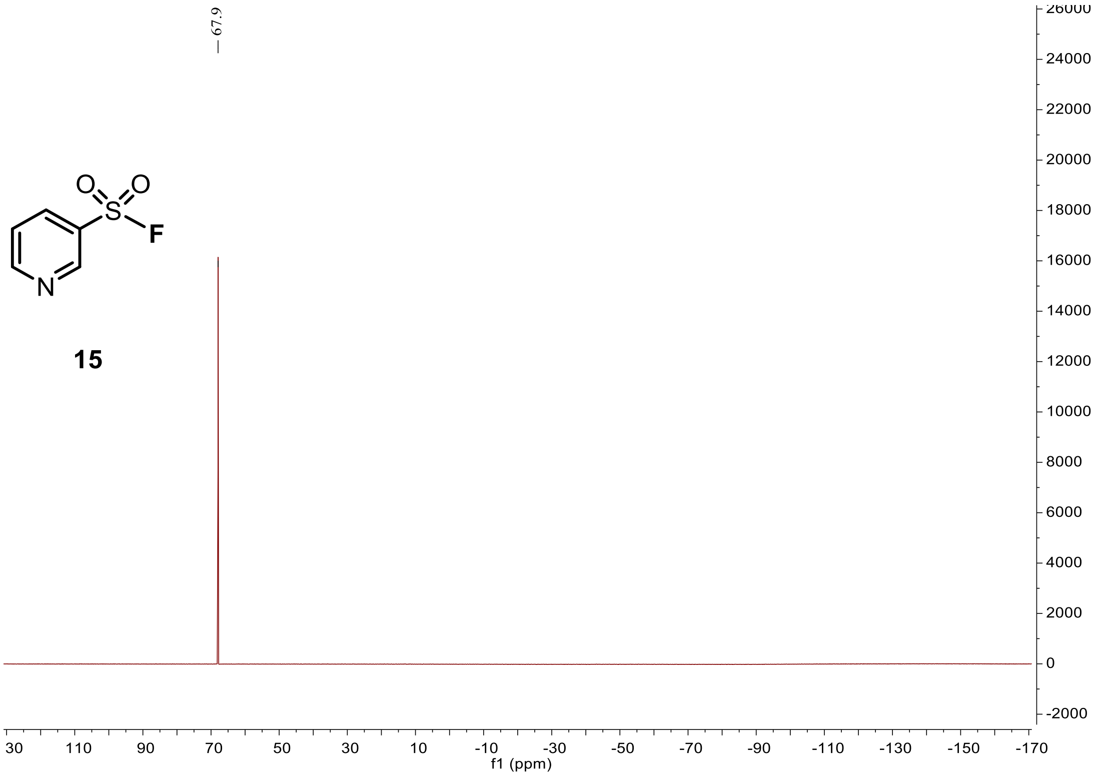

Supplementary Figure 53. ^{19}F-NMR (282 MHz, $CDCl_3$) spectrum of **15**

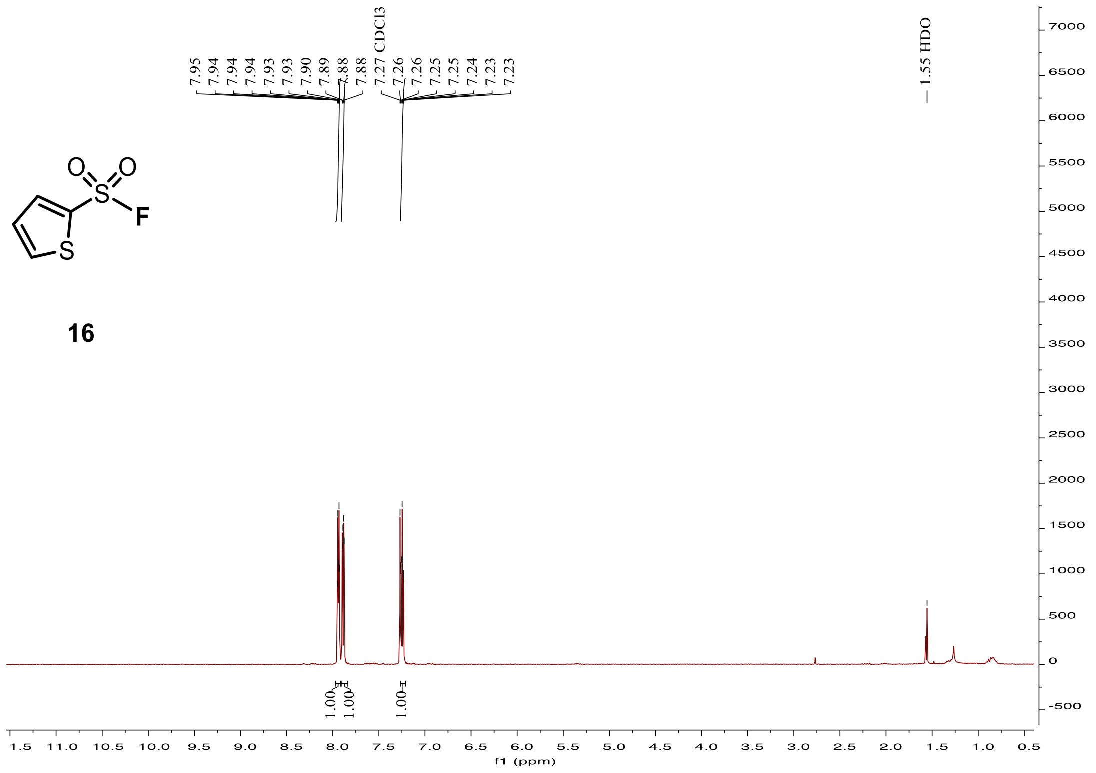

16

Supplementary Figure 54. ^{1}H-NMR (300 MHz, $CDCl_3$) spectrum of **16**

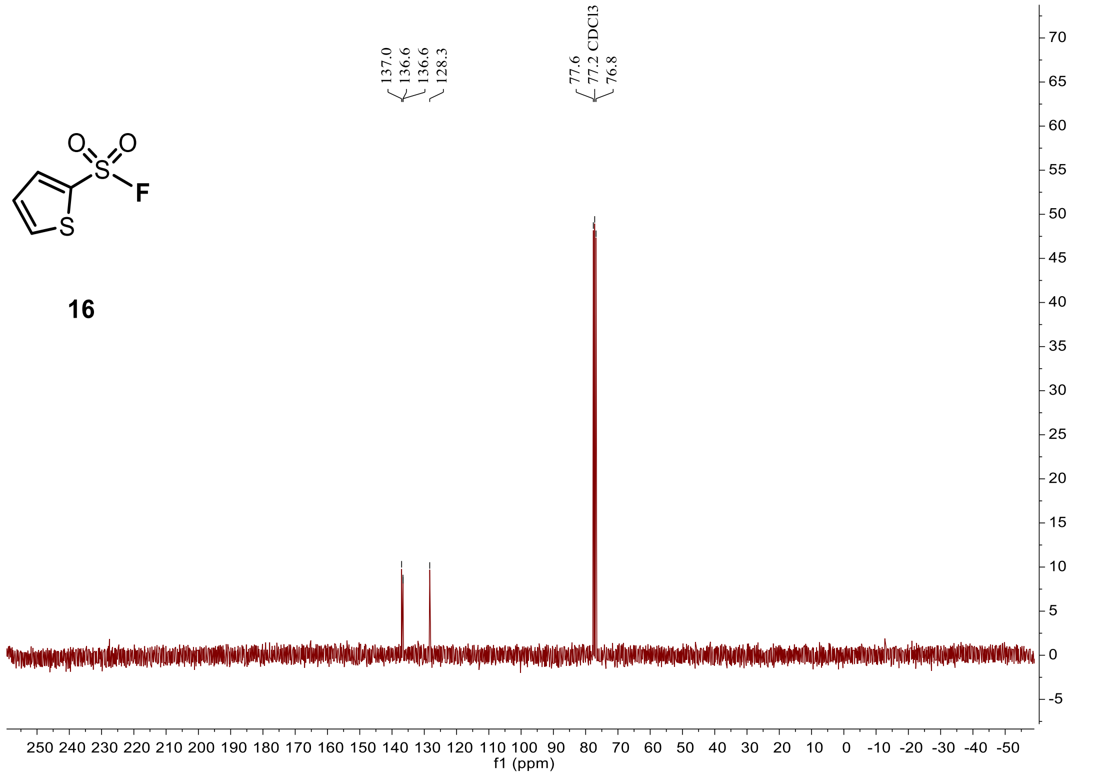

16

Supplementary Figure 55. ^{13}C-NMR (75 MHz, $CDCl_3$) spectrum of **16**

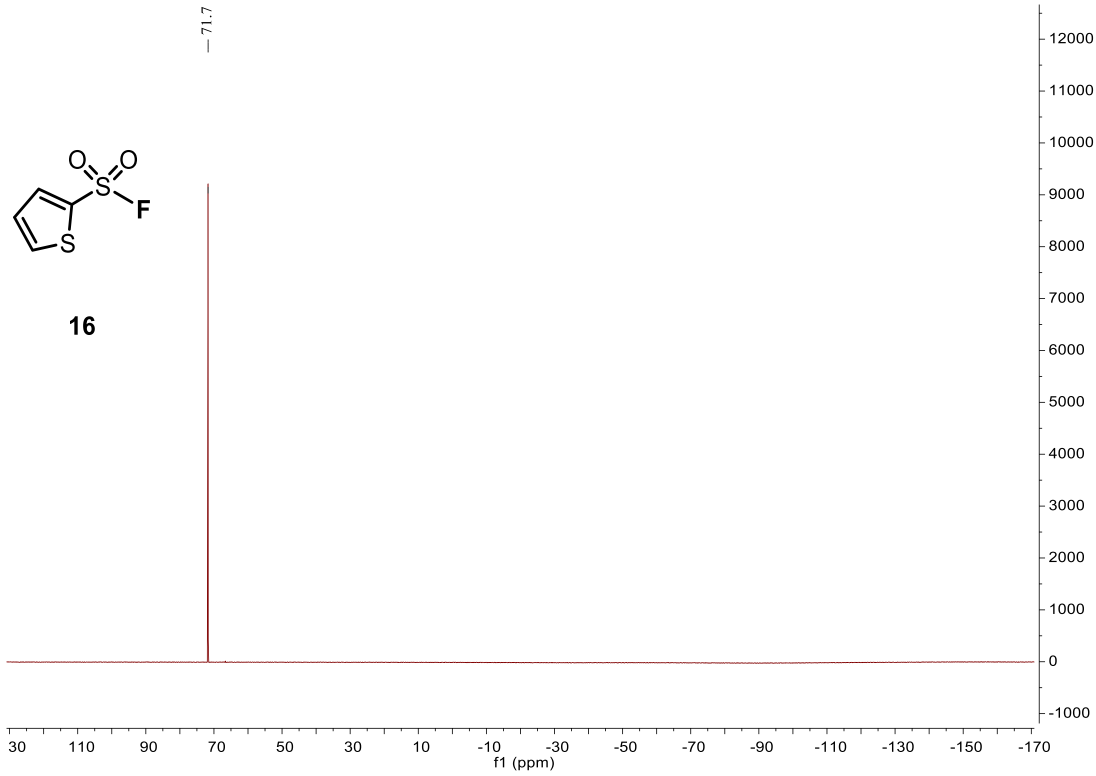

Supplementary Figure 56. ^{19}F-NMR (282 MHz, $CDCl_3$) spectrum of **16**

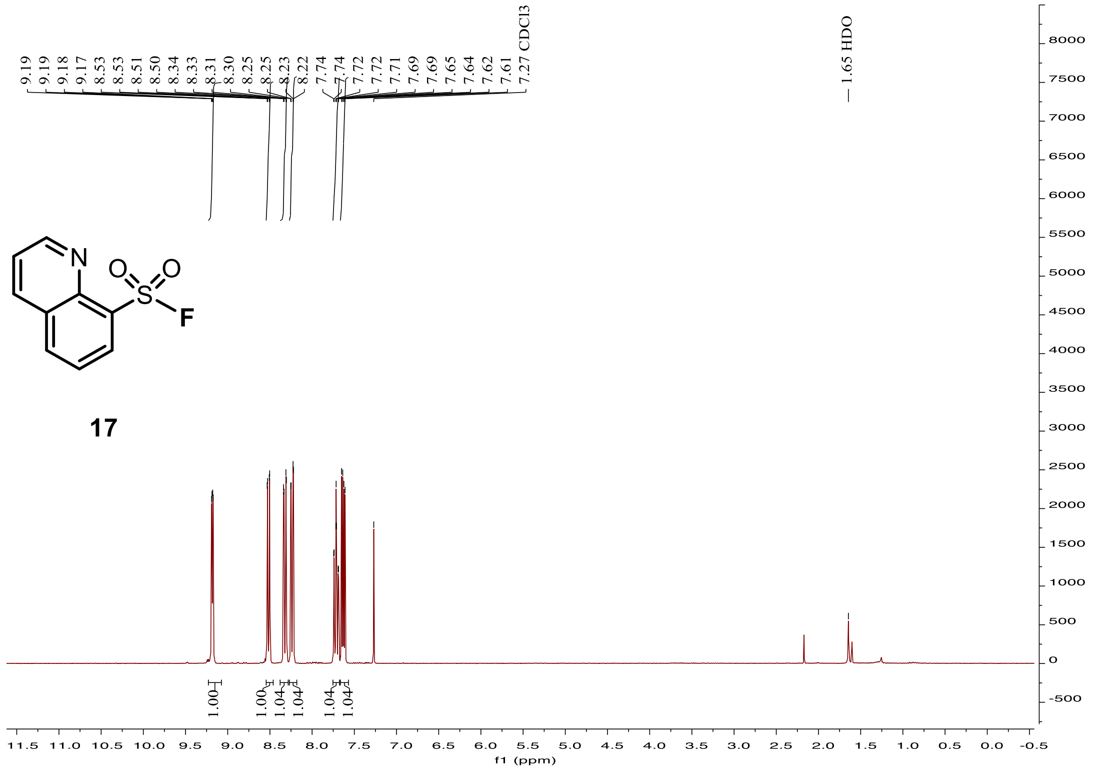

Supplementary Figure 57. ^{1}H-NMR (300 MHz, $CDCl_3$) spectrum of **17**

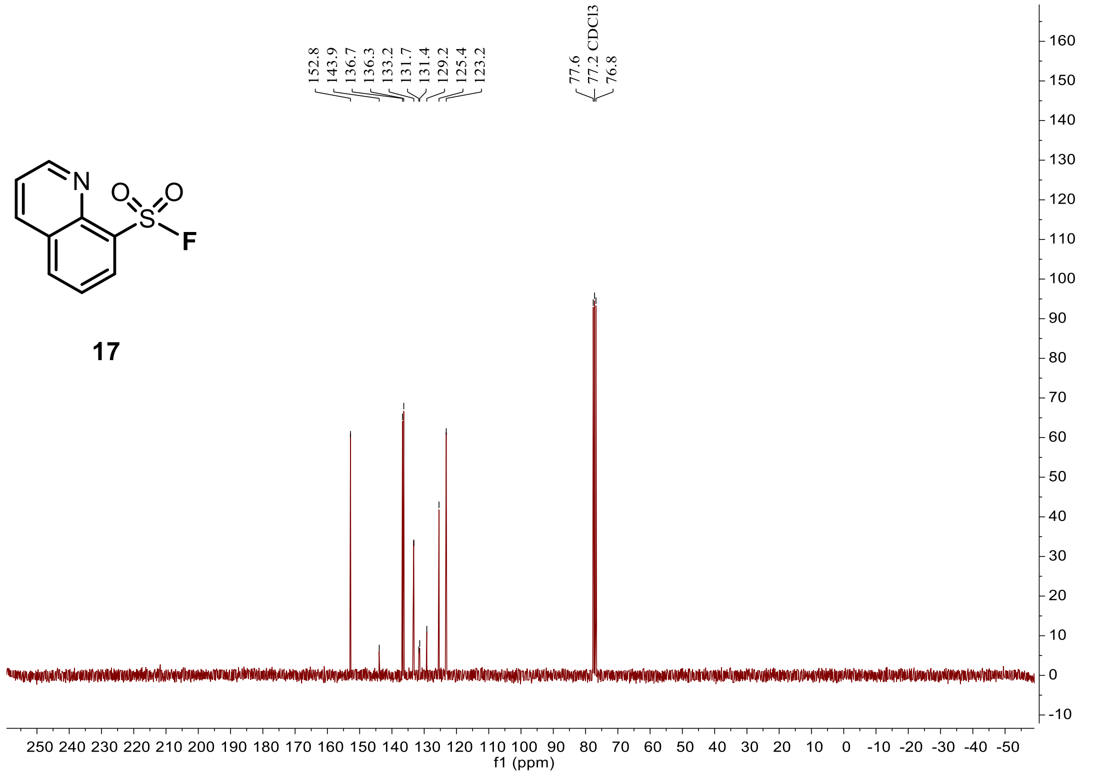

Supplementary Figure 58. ^{13}C-NMR (75 MHz, $CDCl_3$) spectrum of **17**

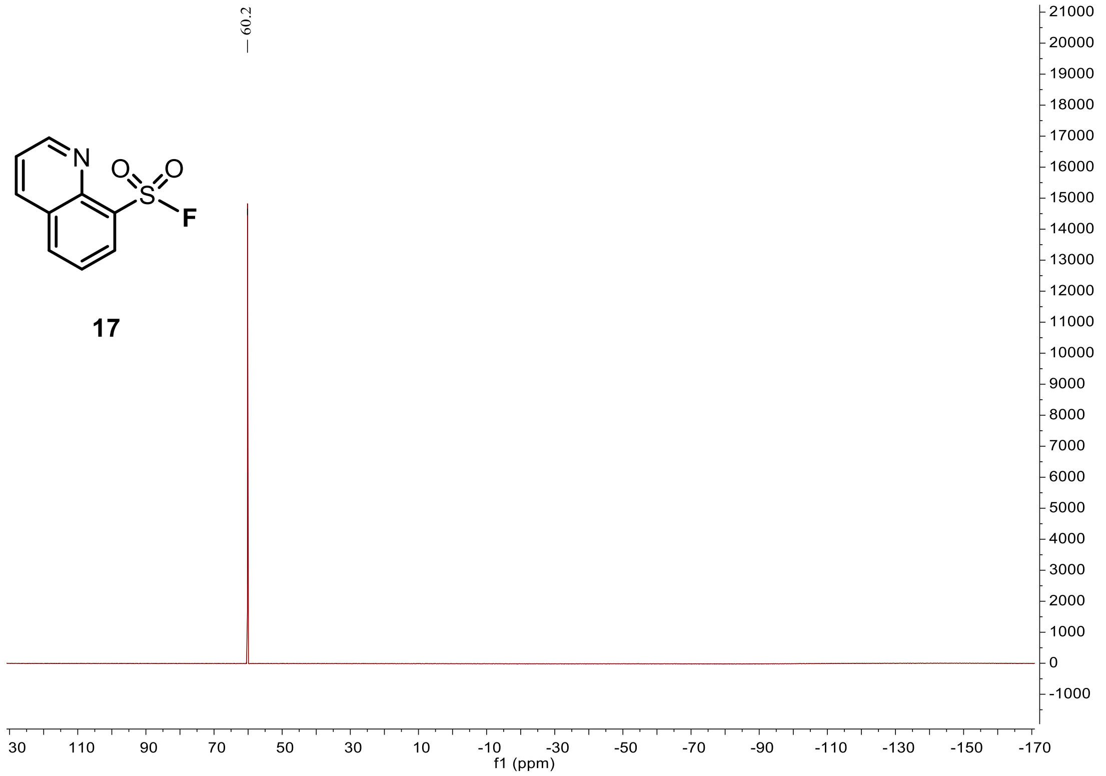

Supplementary Figure 59. ^{19}F-NMR (282 MHz, $CDCl_3$) spectrum of **17**

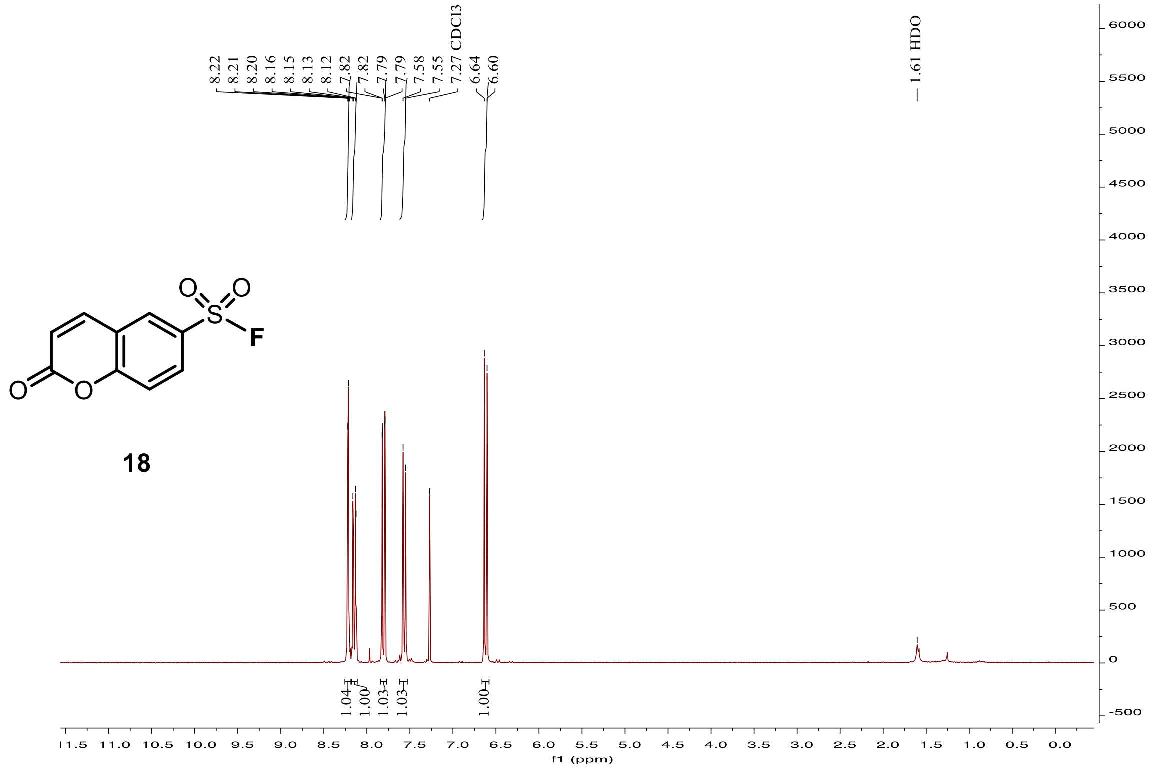

Supplementary Figure 60. ^{1}H-NMR (300 MHz, $CDCl_3$) spectrum of **18**

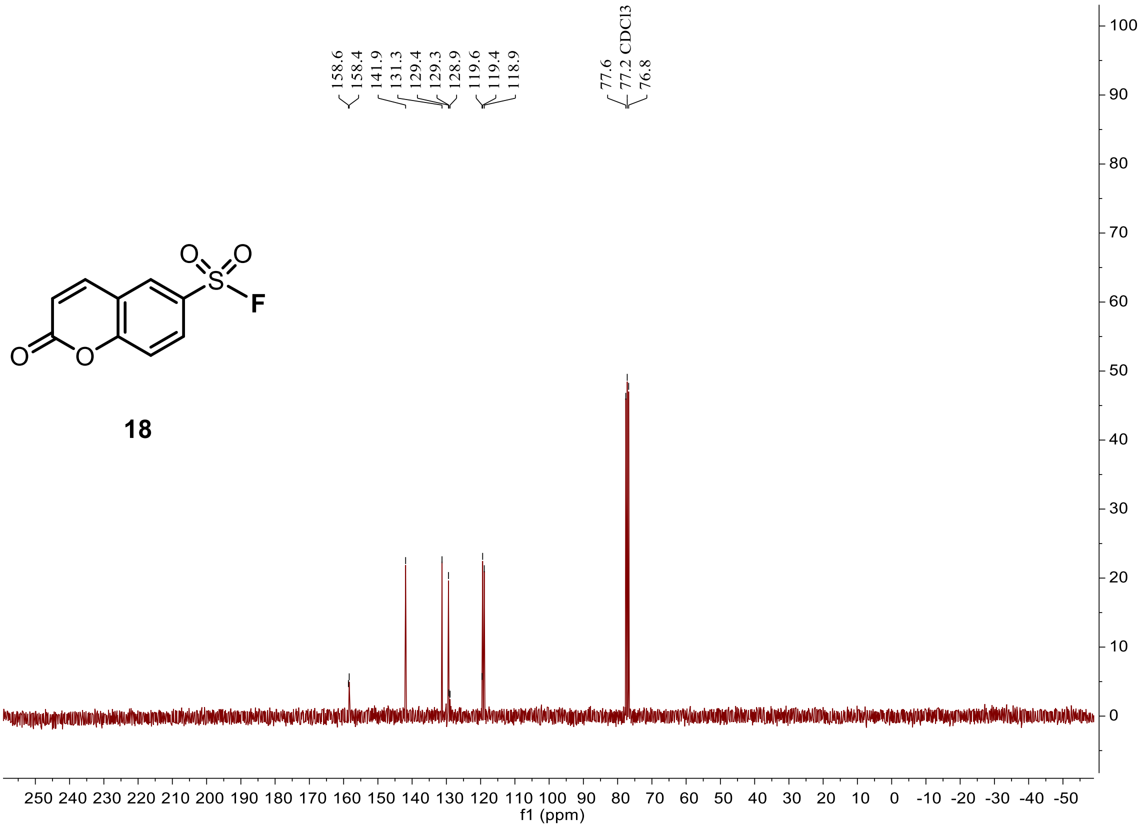

Supplementary Figure 61. ^{13}C-NMR (75 MHz, $CDCl_3$) spectrum of **18**

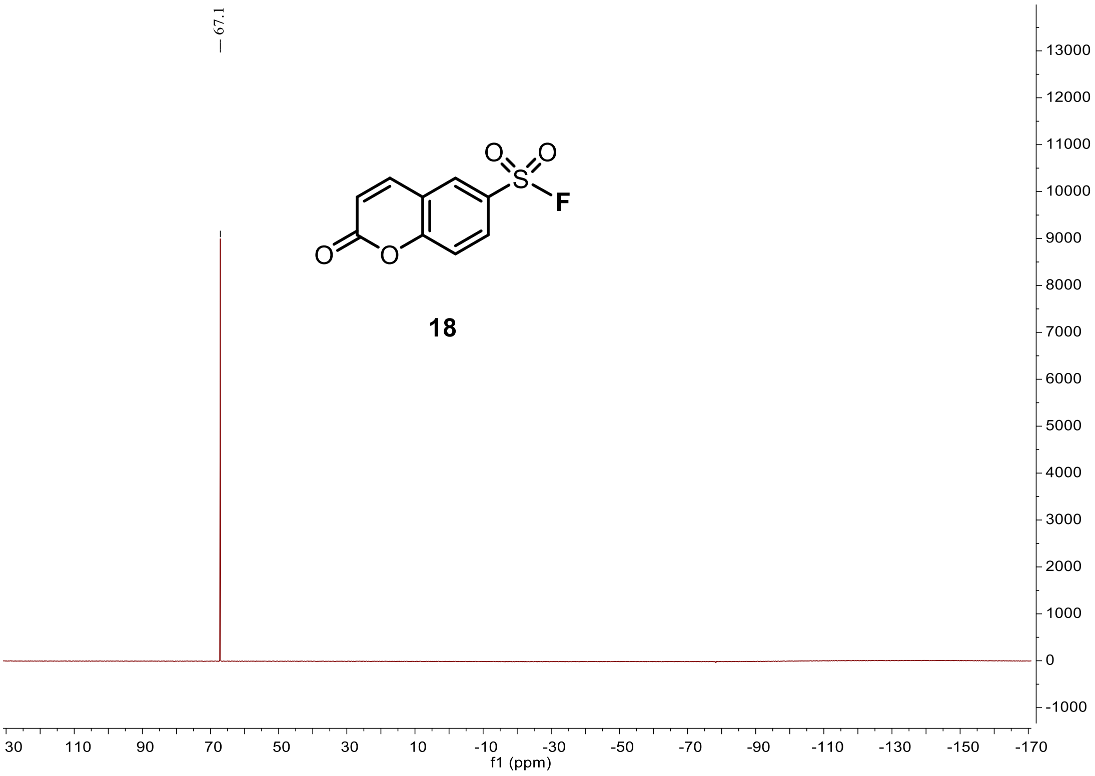

Supplementary Figure 62. ^{19}F-NMR (282 MHz, $CDCl_3$) spectrum of **18**

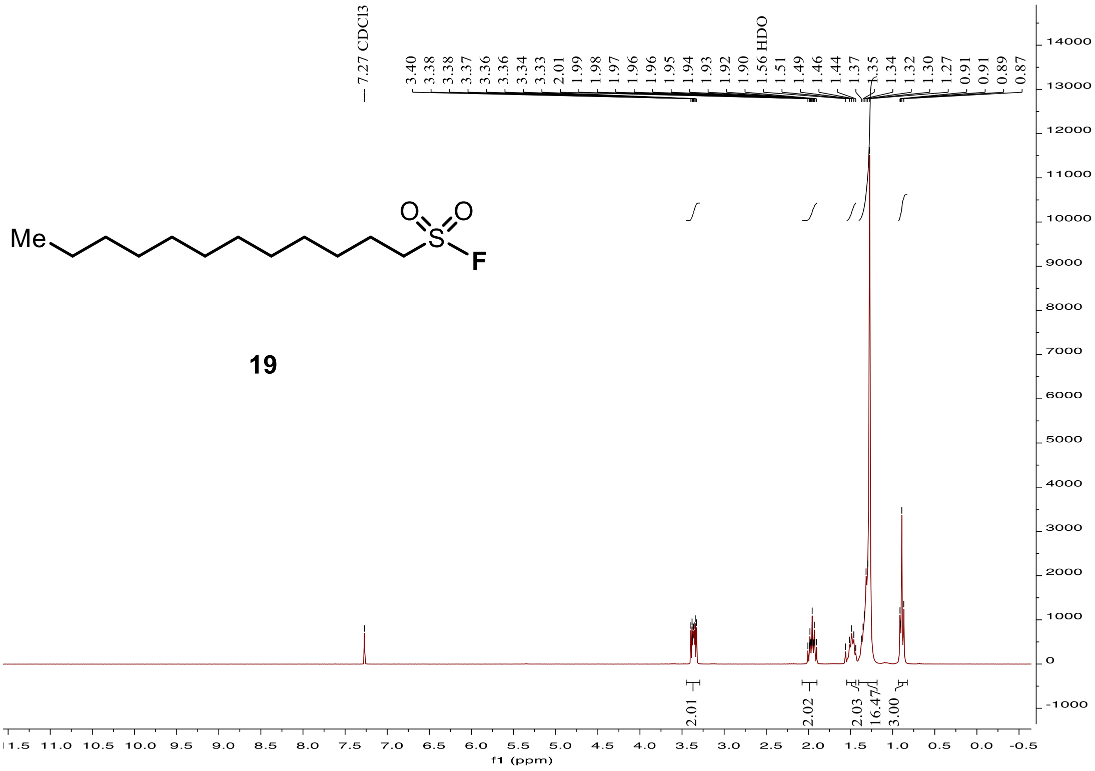

Supplementary Figure 63. ^{1}H-NMR (300 MHz, $CDCl_3$) spectrum of **19**

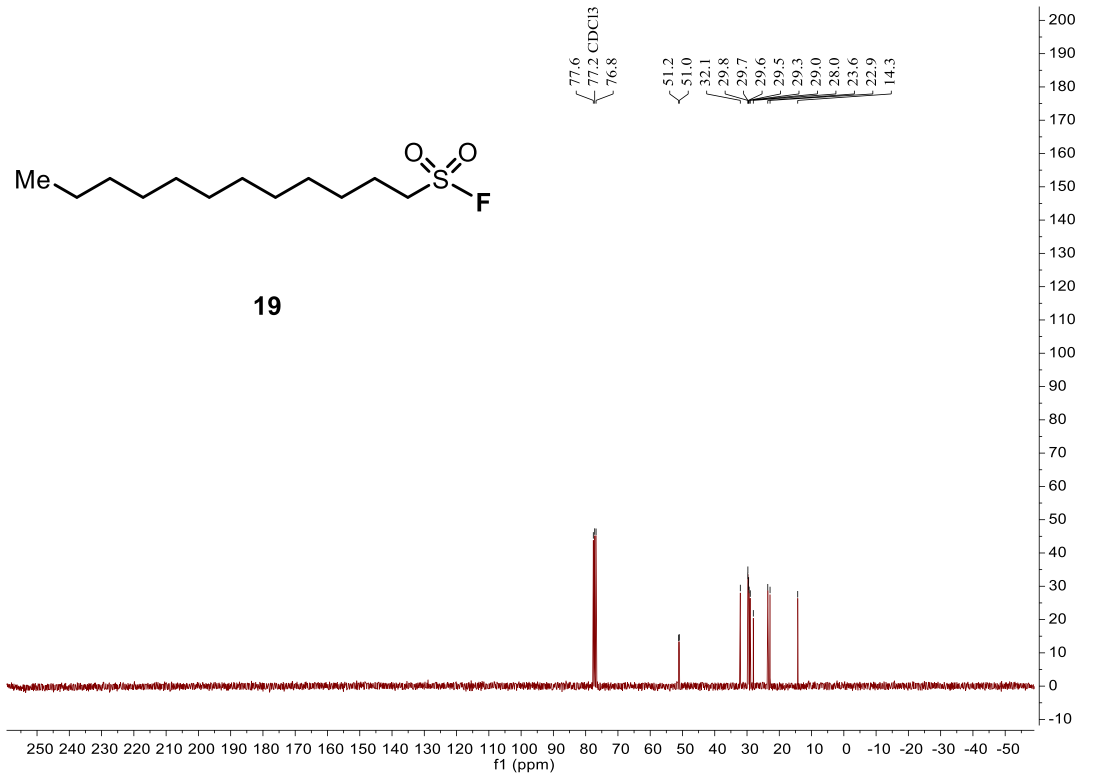

Supplementary Figure 64. ^{13}C-NMR (75 MHz, $CDCl_3$) spectrum of **19**

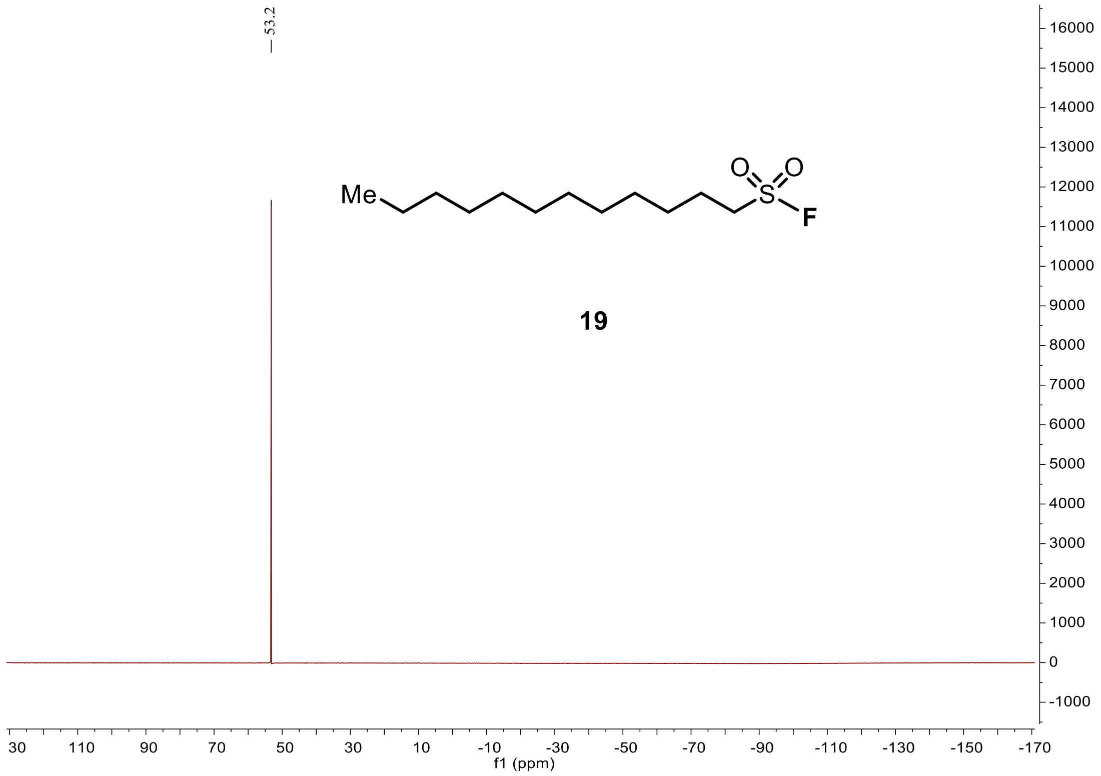

Supplementary Figure 65. ^{19}F-NMR (282 MHz, $CDCl_3$) spectrum of **19**

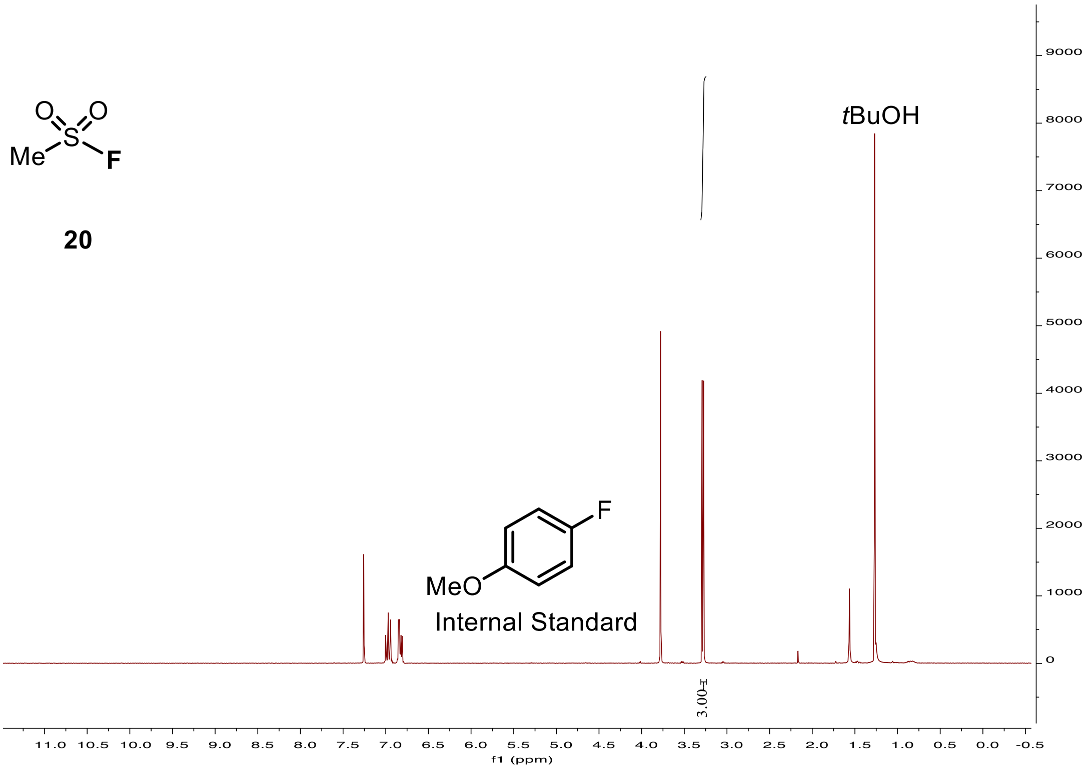

Supplementary Figure 66. Crude ^{1}H-NMR (300 MHz, $CDCl_3$) spectrum of **20**

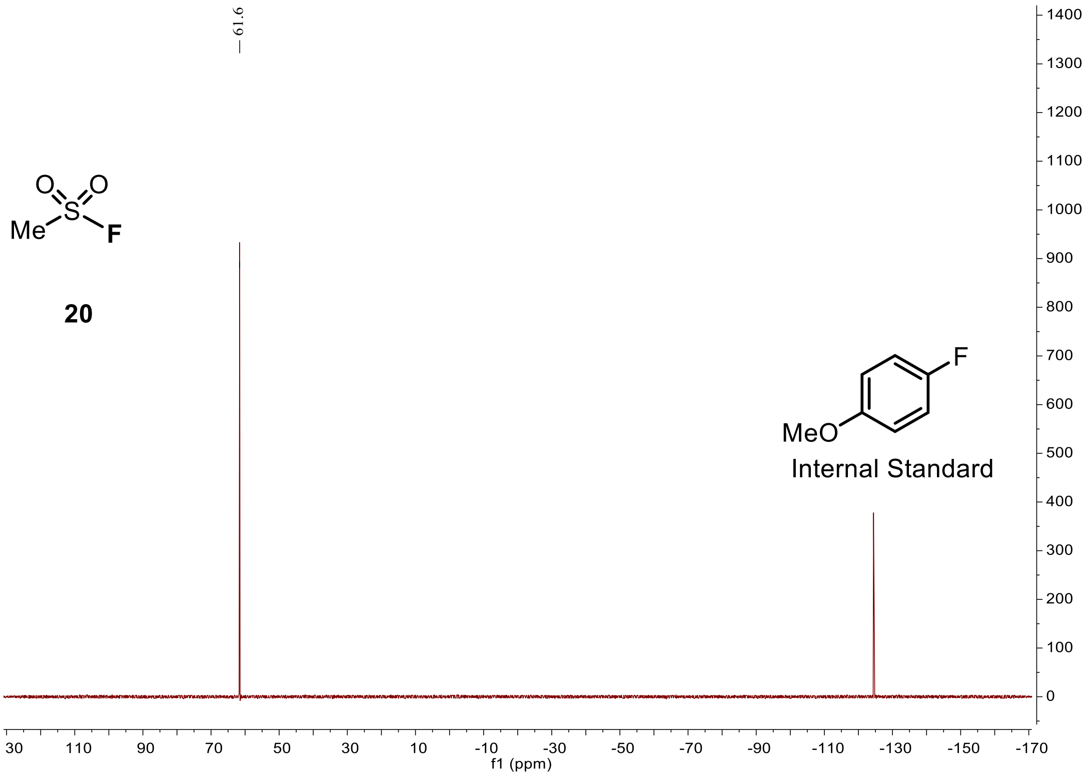

Supplementary Figure 67. Crude ^{19}F-NMR (282 MHz, $CDCl_3$) spectrum of **20**

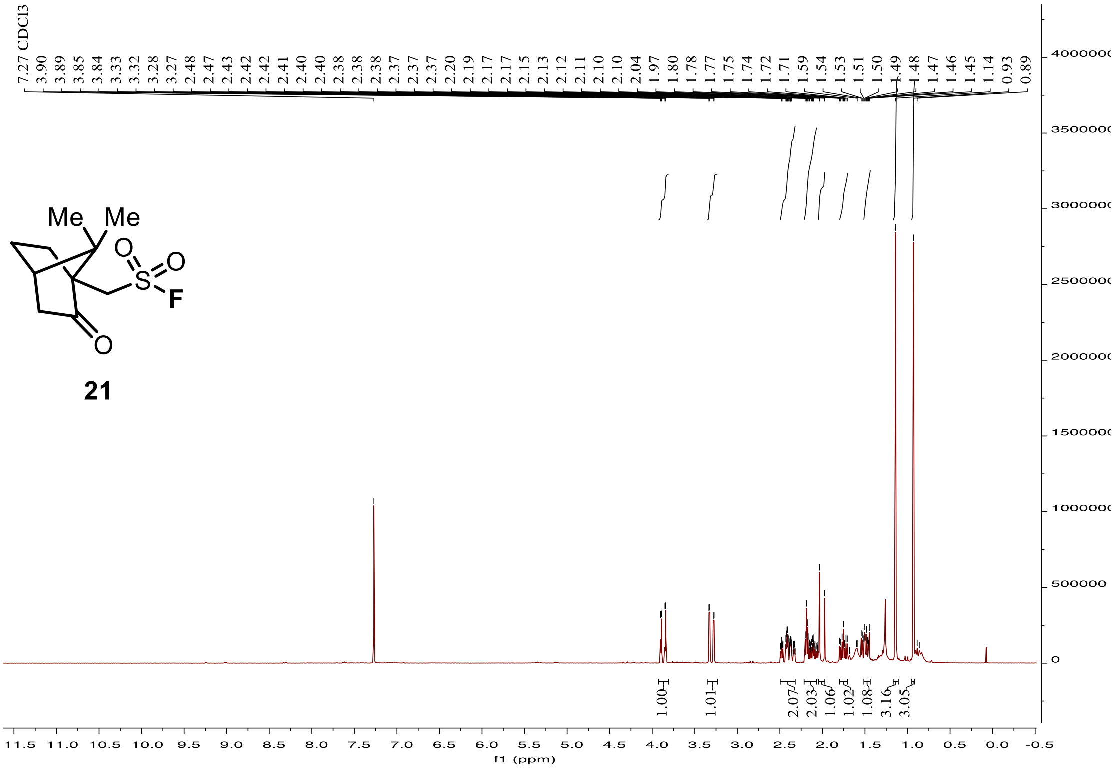

Supplementary Figure 68. ^{1}H-NMR (300 MHz, $CDCl_3$) spectrum of **21**

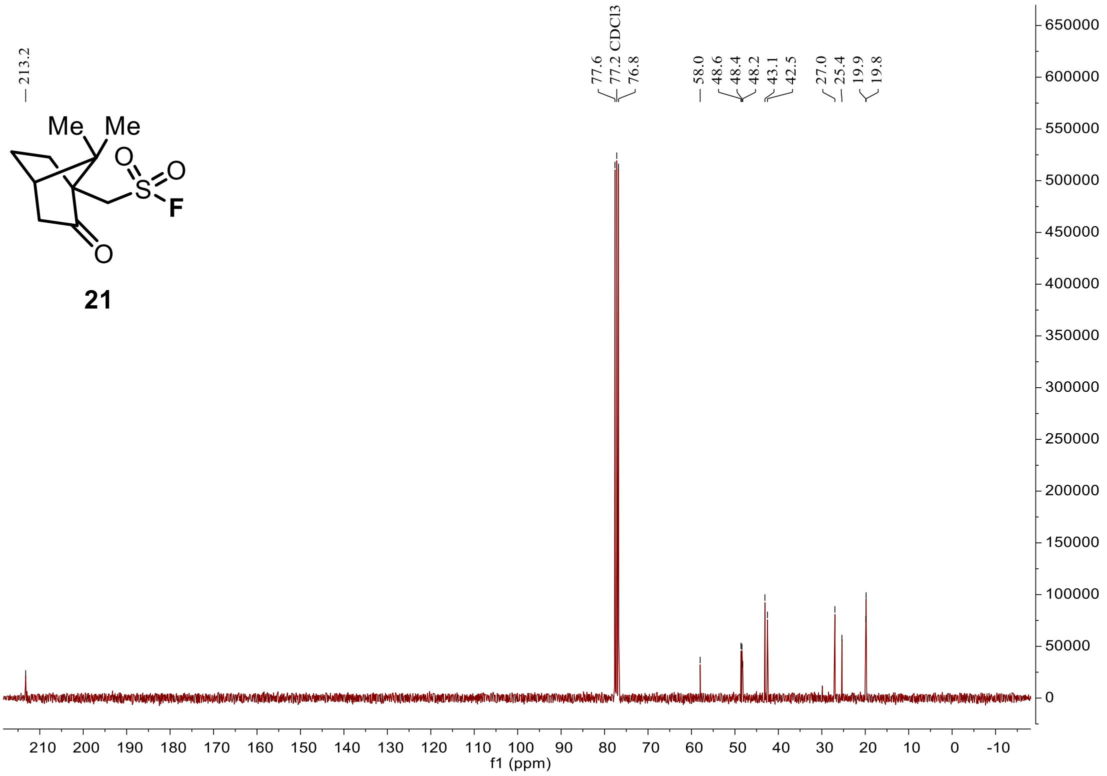

Supplementary Figure 69. ^{13}C-NMR (75 MHz, $CDCl_3$) spectrum of **21**

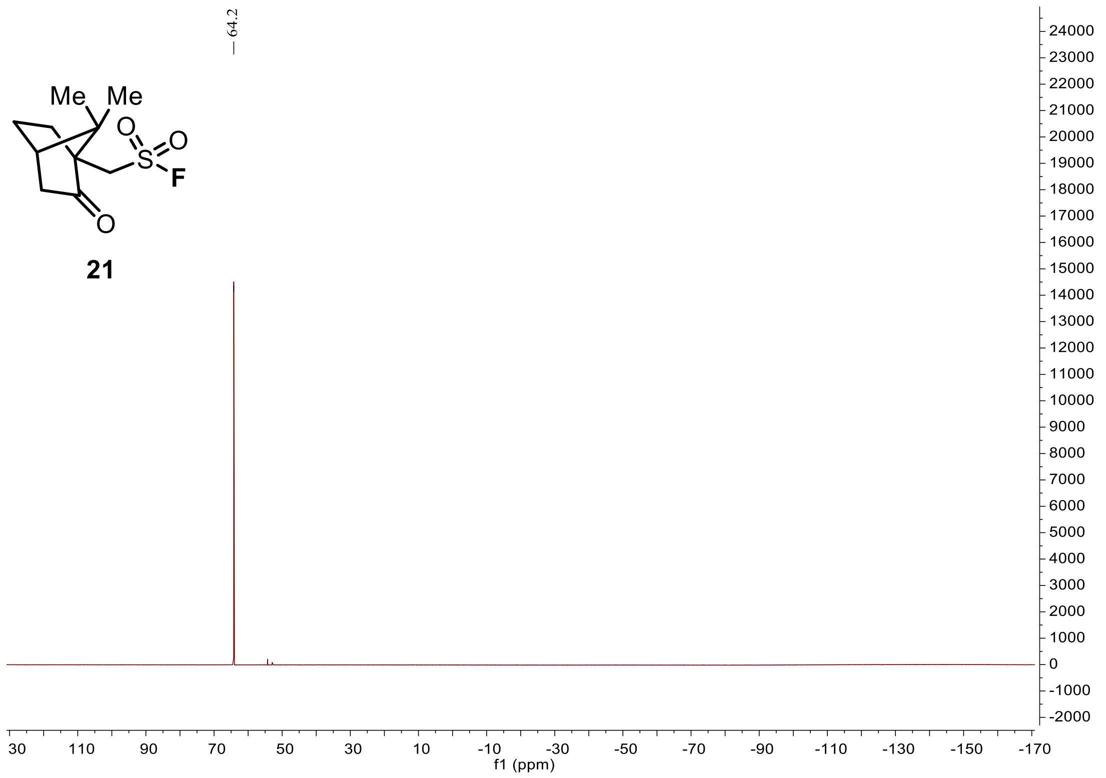

Supplementary Figure 70. ^{19}F-NMR (282 MHz, $CDCl_3$) spectrum of **21**

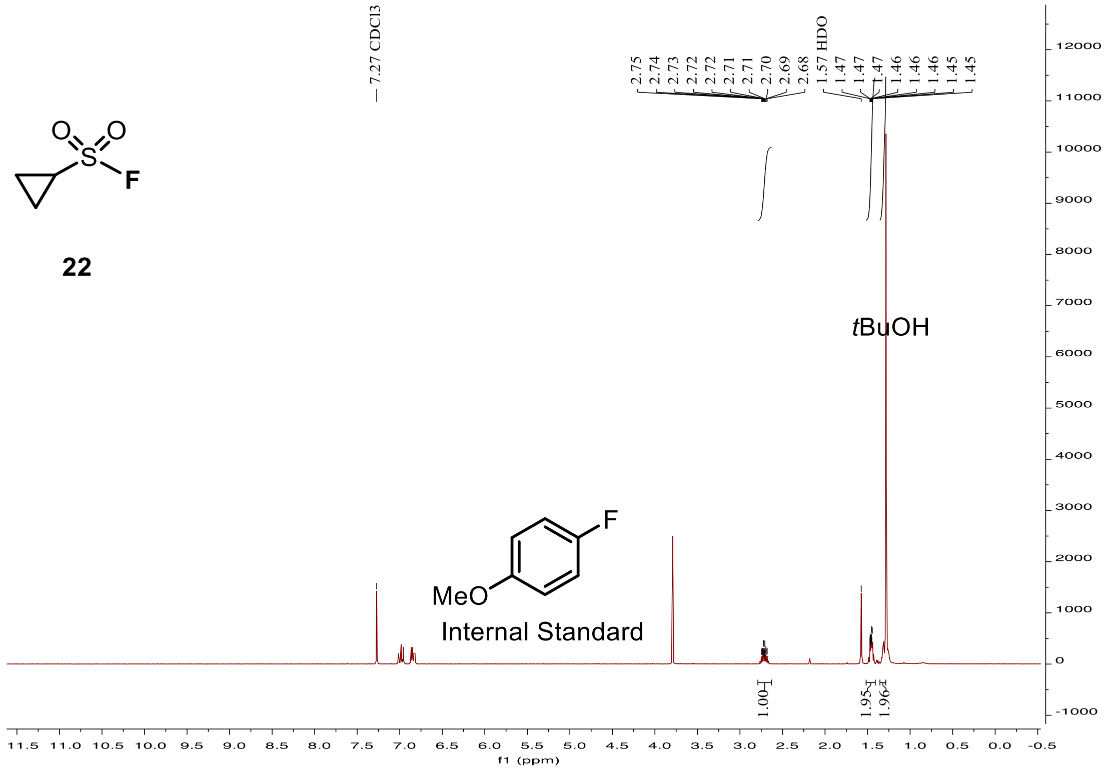

Supplementary Figure 71. Crude ^{1}H-NMR (300 MHz, $CDCl_3$) spectrum of **22**

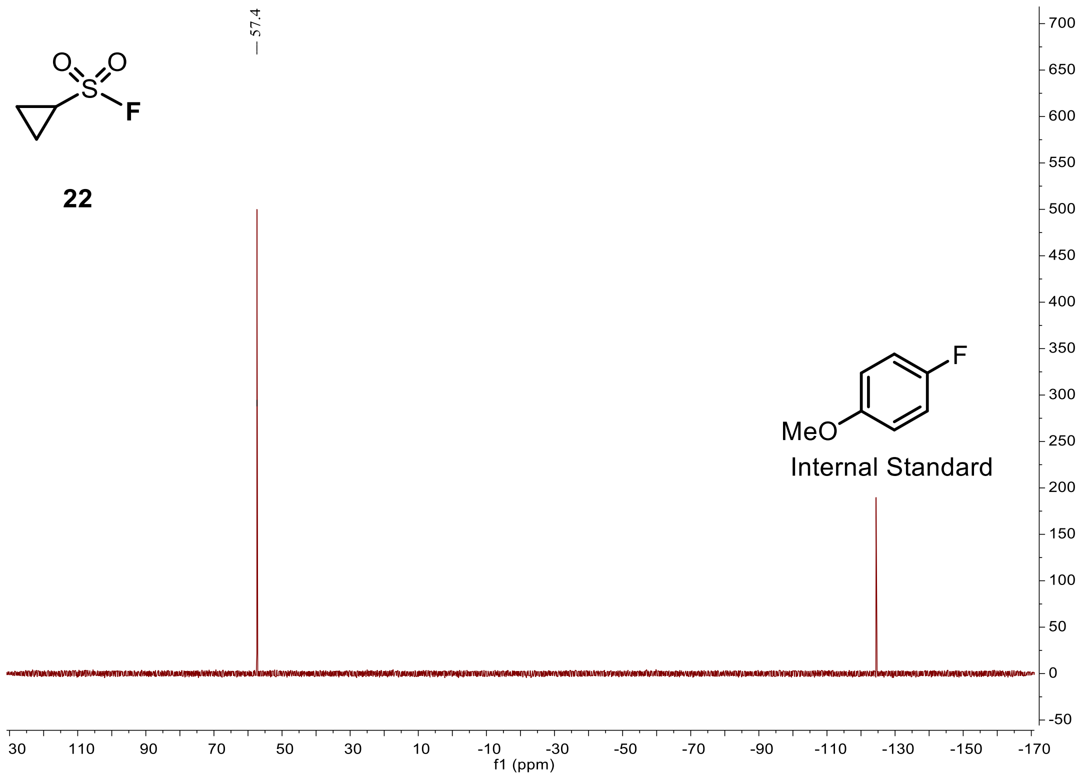

Supplementary Figure 72. Crude ^{19}F-NMR (282 MHz, $CDCl_3$) spectrum of **22**

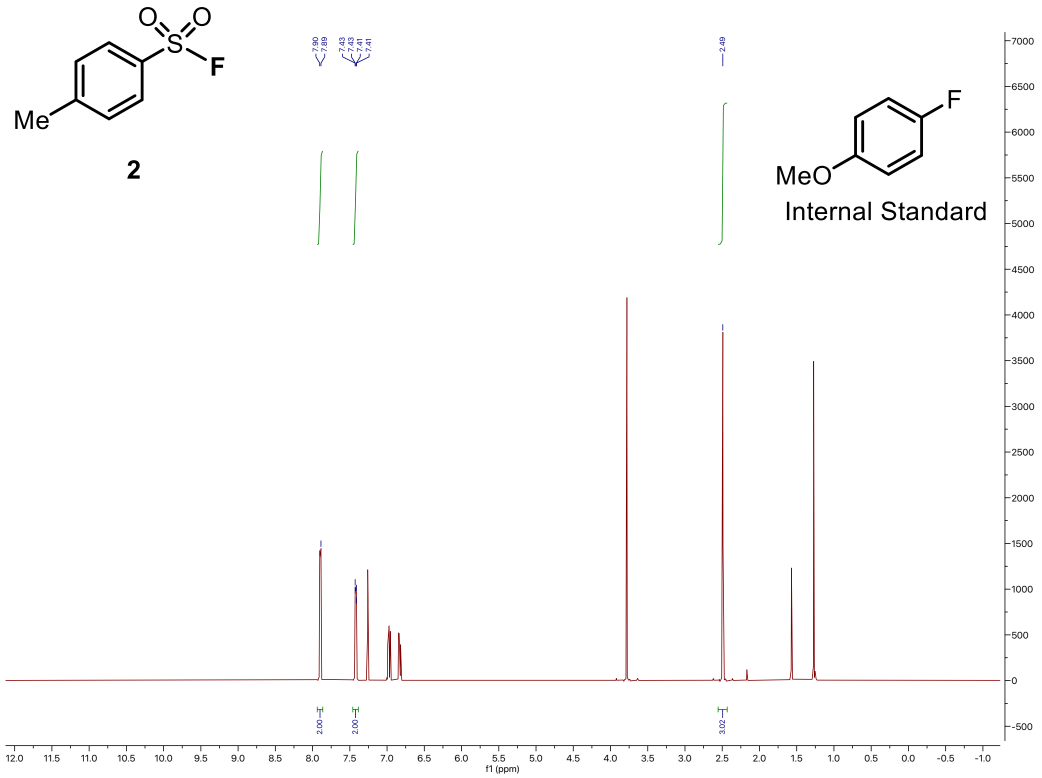

Supplementary Figure 73. Crude ^{19}F-NMR spectrum after ball milling of **38**

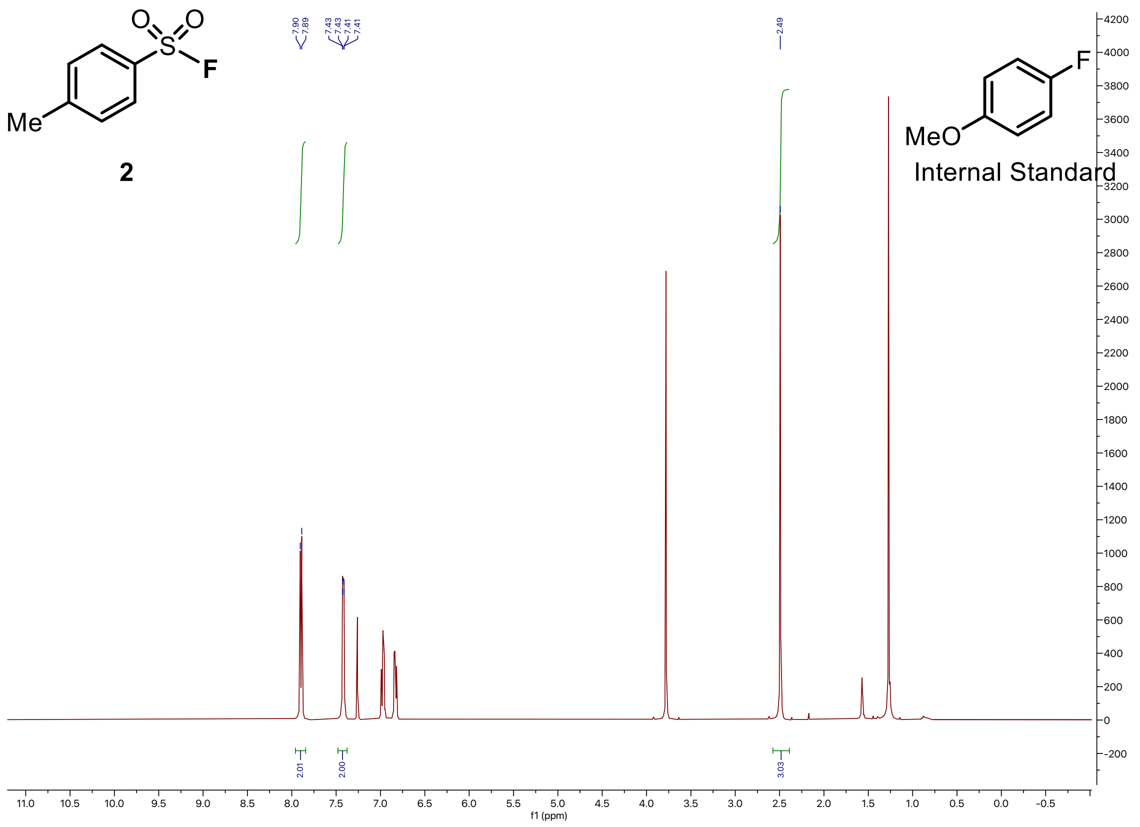

Supplementary Figure 74. Crude ^{19}F-NMR spectrum after ball milling of **39**

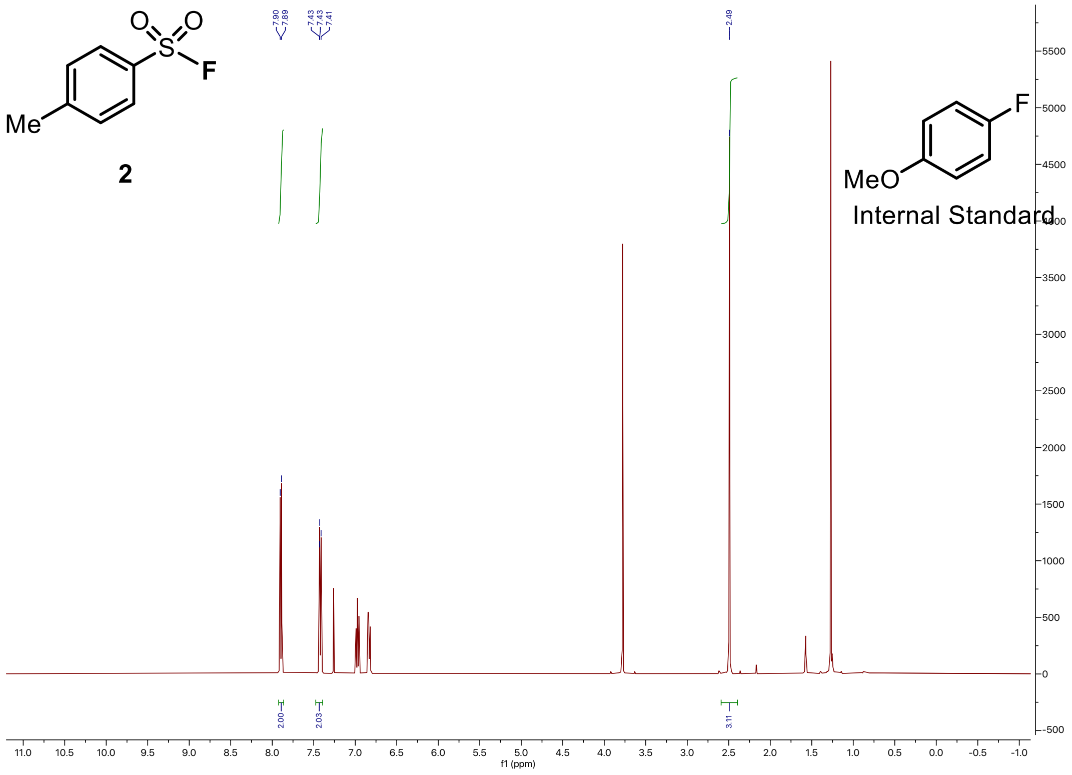

Supplementary Figure 75. Crude ^{19}F-NMR spectrum after ball milling of **40**

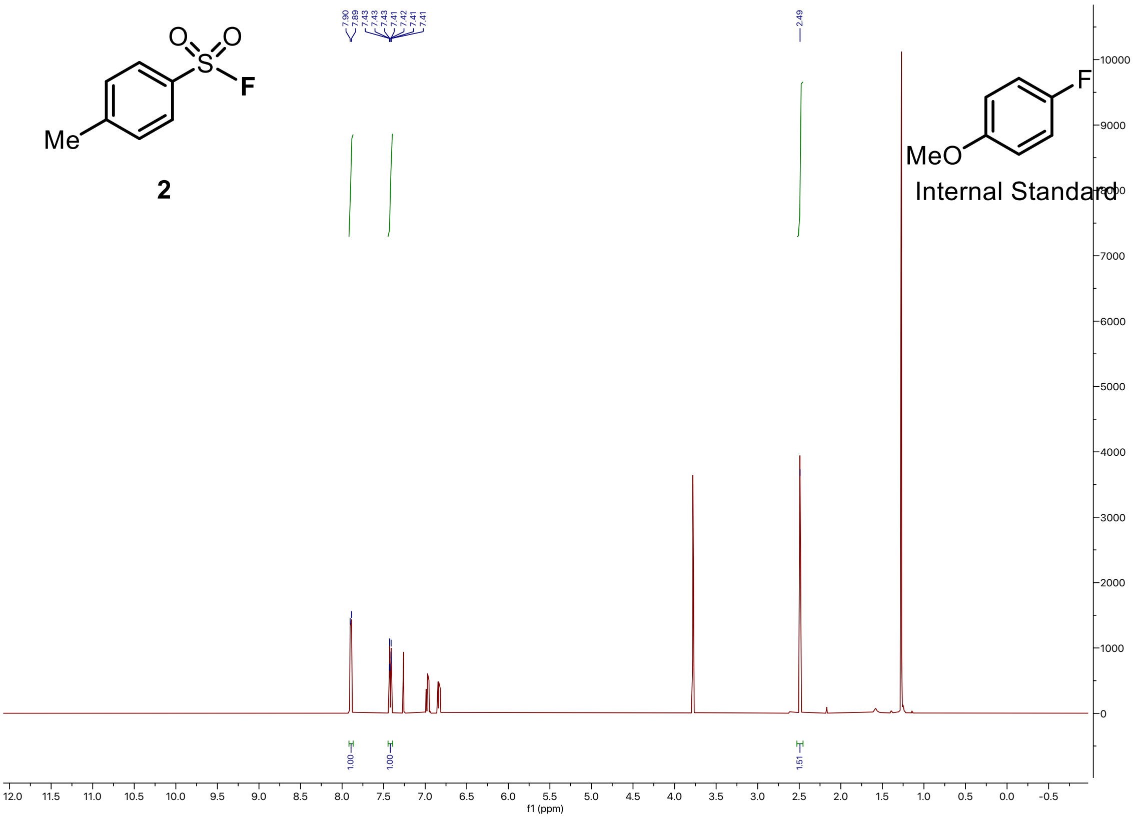

Supplementary Figure 76. Crude ^{19}F-NMR spectrum after ball milling of **41**

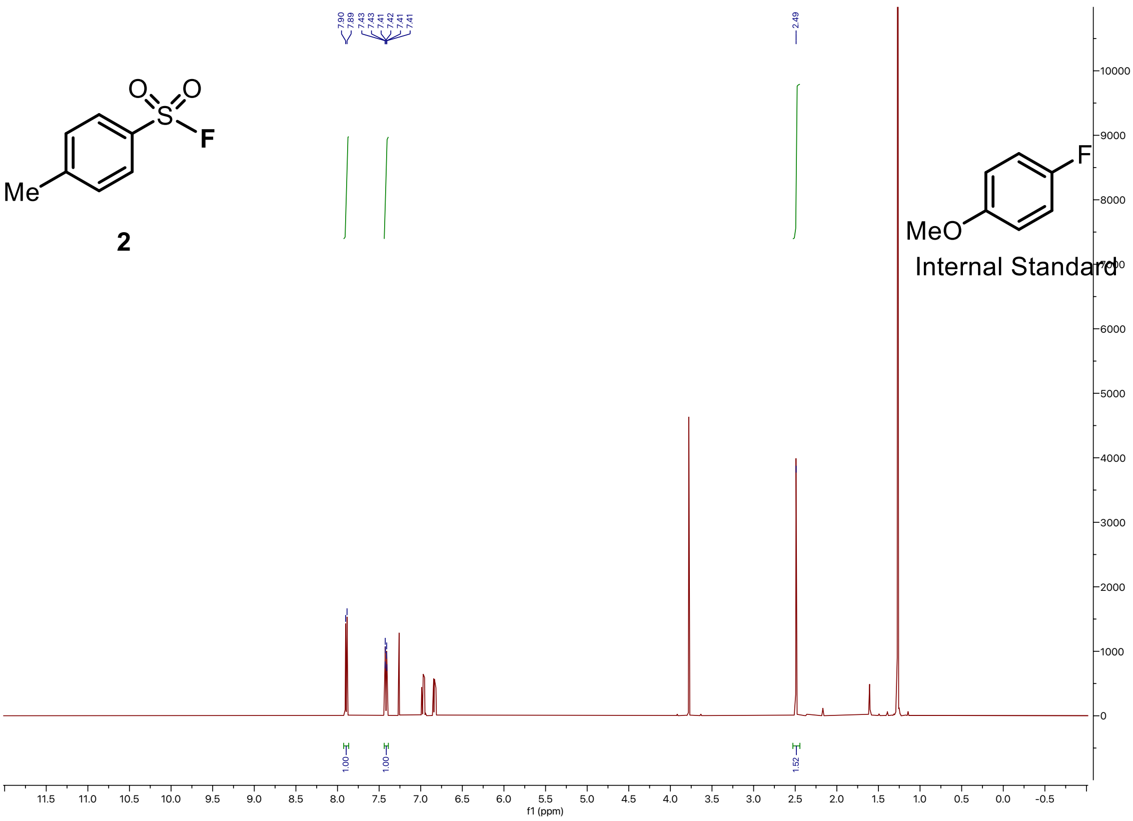

Supplementary Figure 77. Crude ^{19}F-NMR spectrum after ball milling of **42**

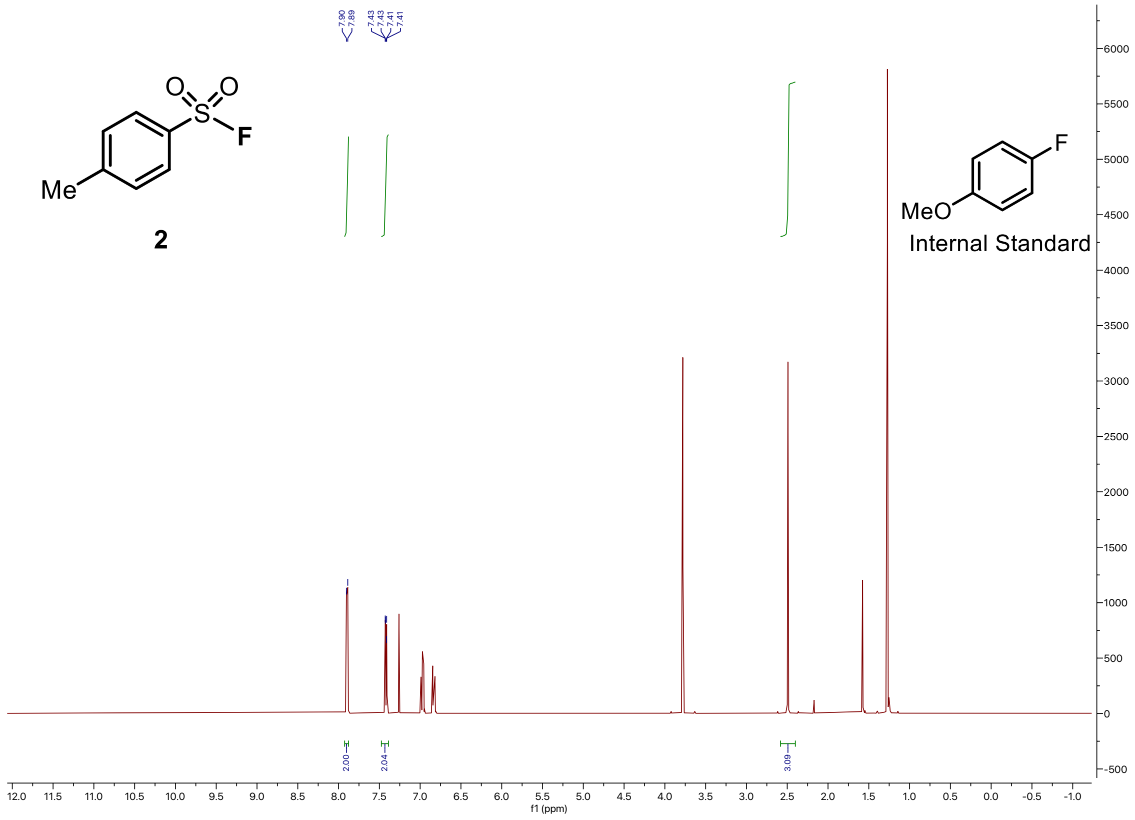

Supplementary Figure 78. Crude ^{19}F-NMR spectrum after ball milling of **43**

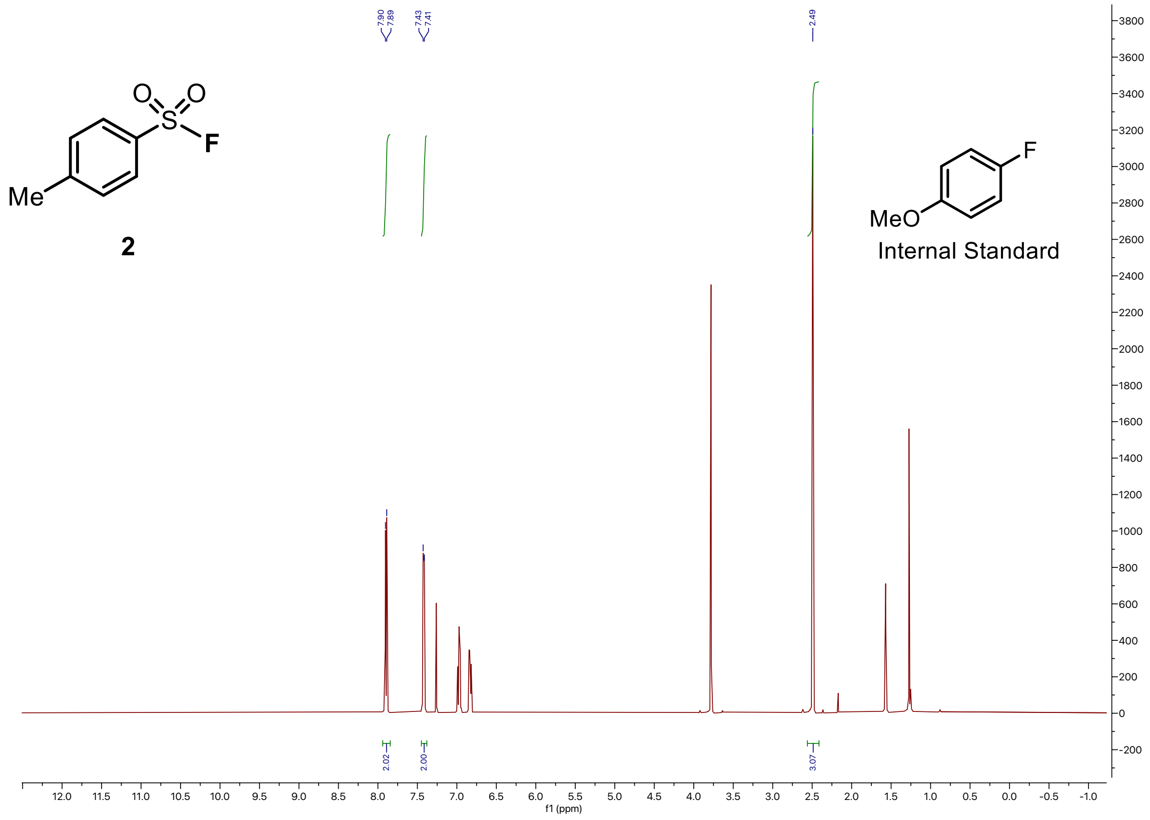

Supplementary Figure 79. Crude ^{19}F-NMR spectrum after ball milling of **44**

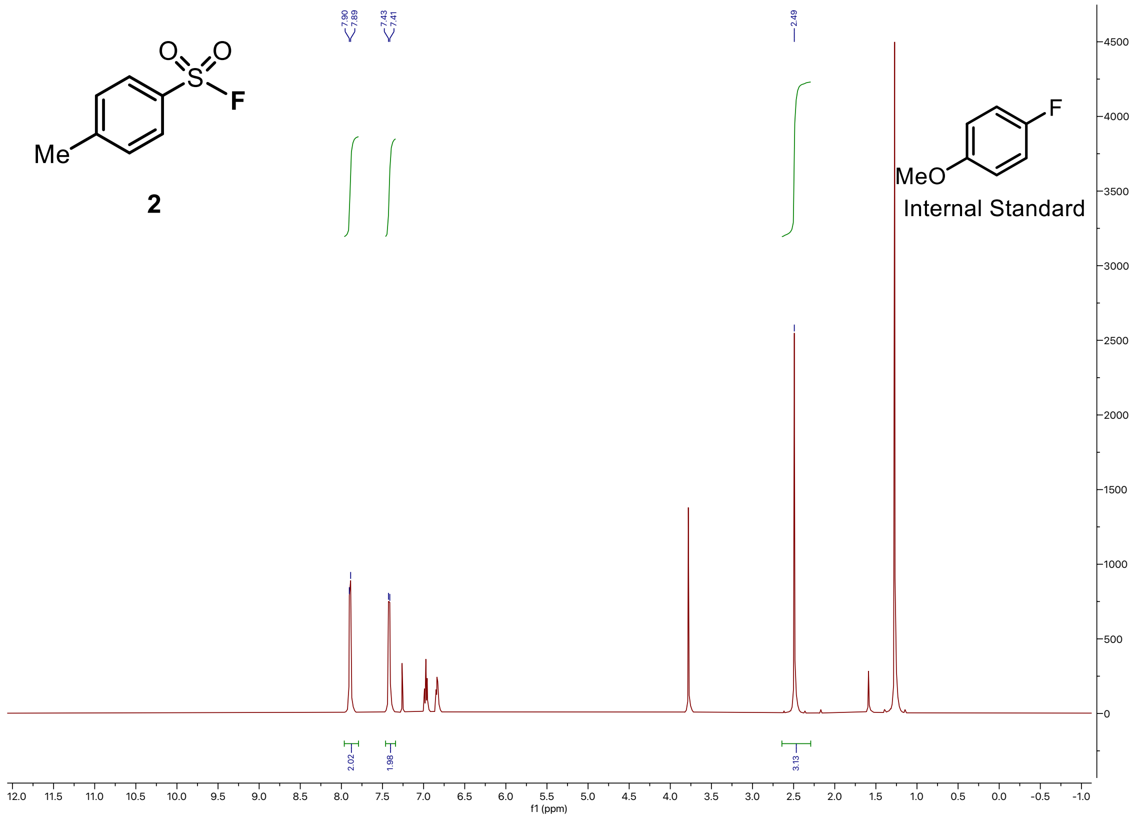

Supplementary Figure 80. Crude ^{19}F-NMR spectrum after ball milling of **45**

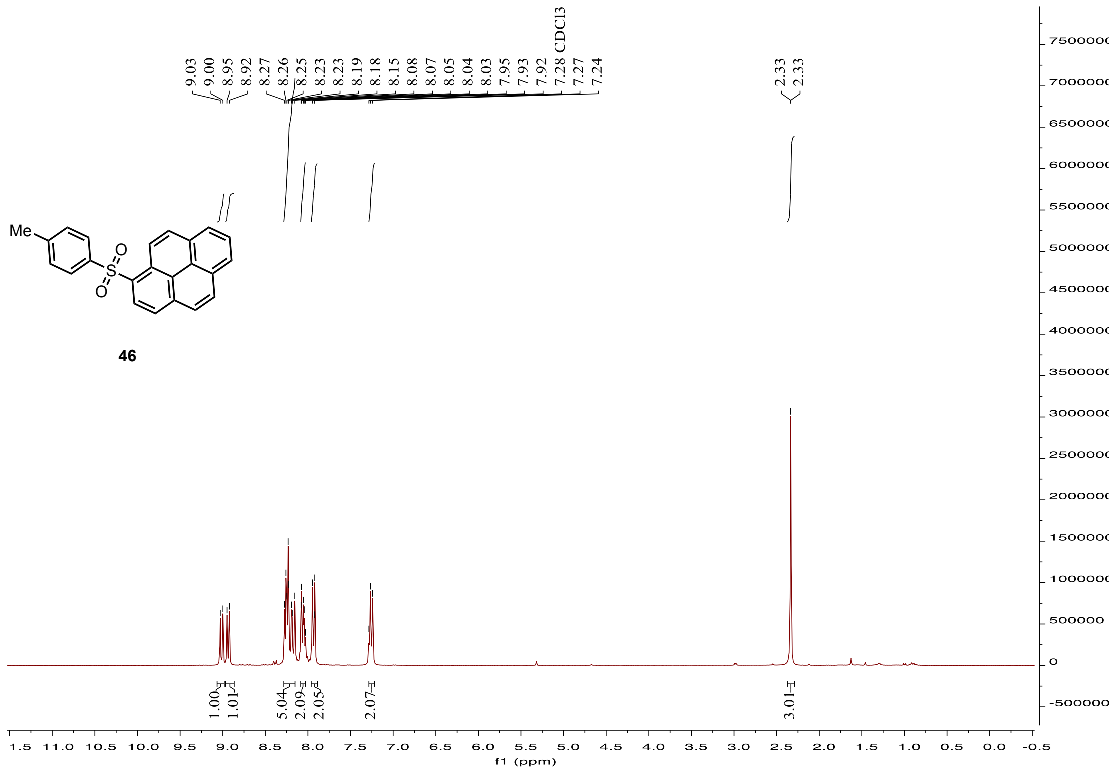

Supplementary Figure 81. ^{1}H NMR Spectrum of **46**

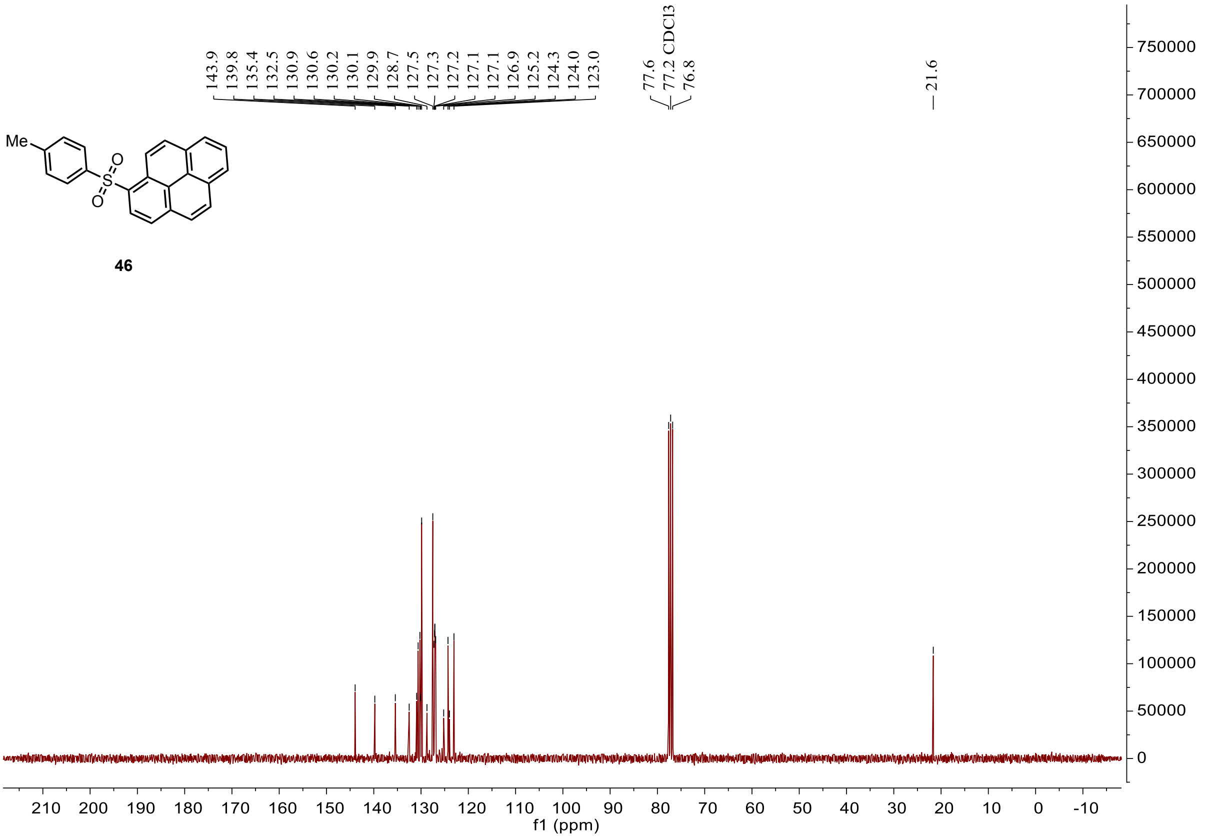

Supplementary Figure 82. ^{13}C-NMR (75 MHz, $CDCl_3$) spectrum of **46**

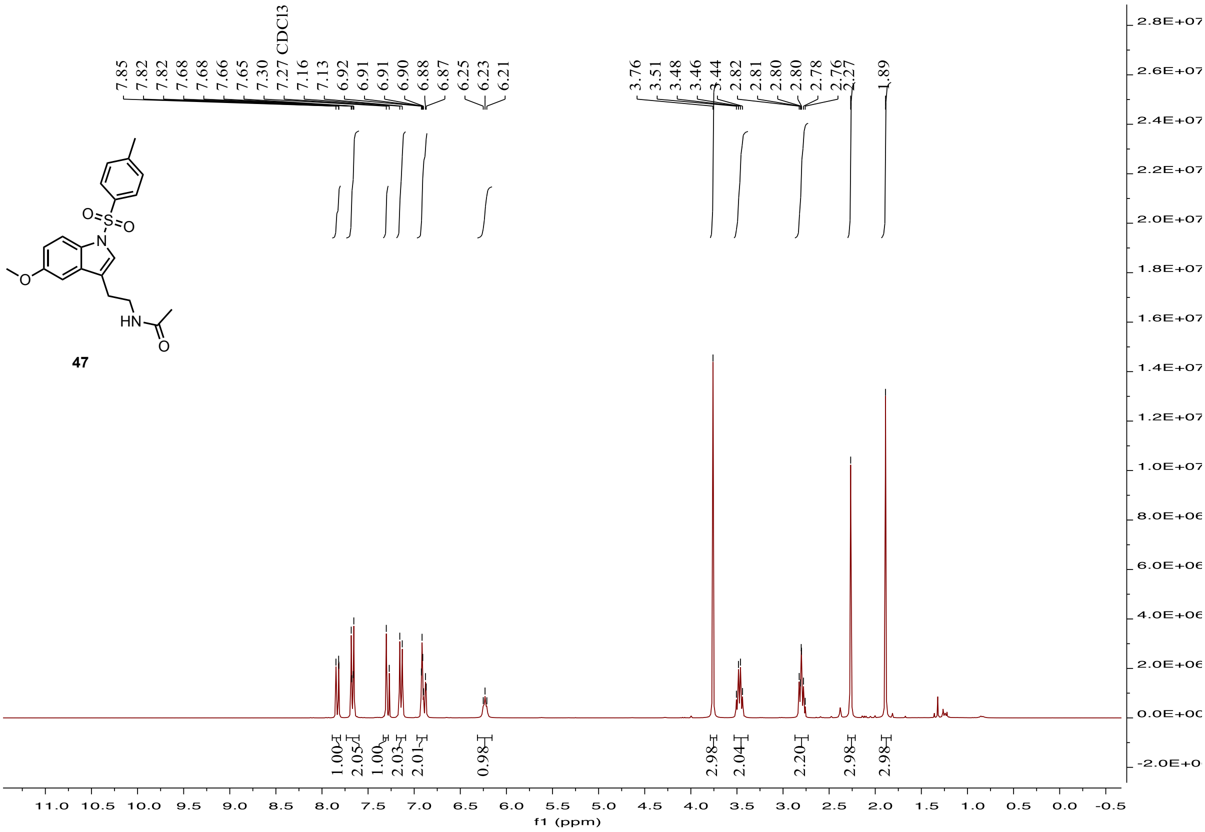

Supplementary Figure 83. ^{1}H-NMR (300 MHz, $CDCl_3$) spectrum of **47**

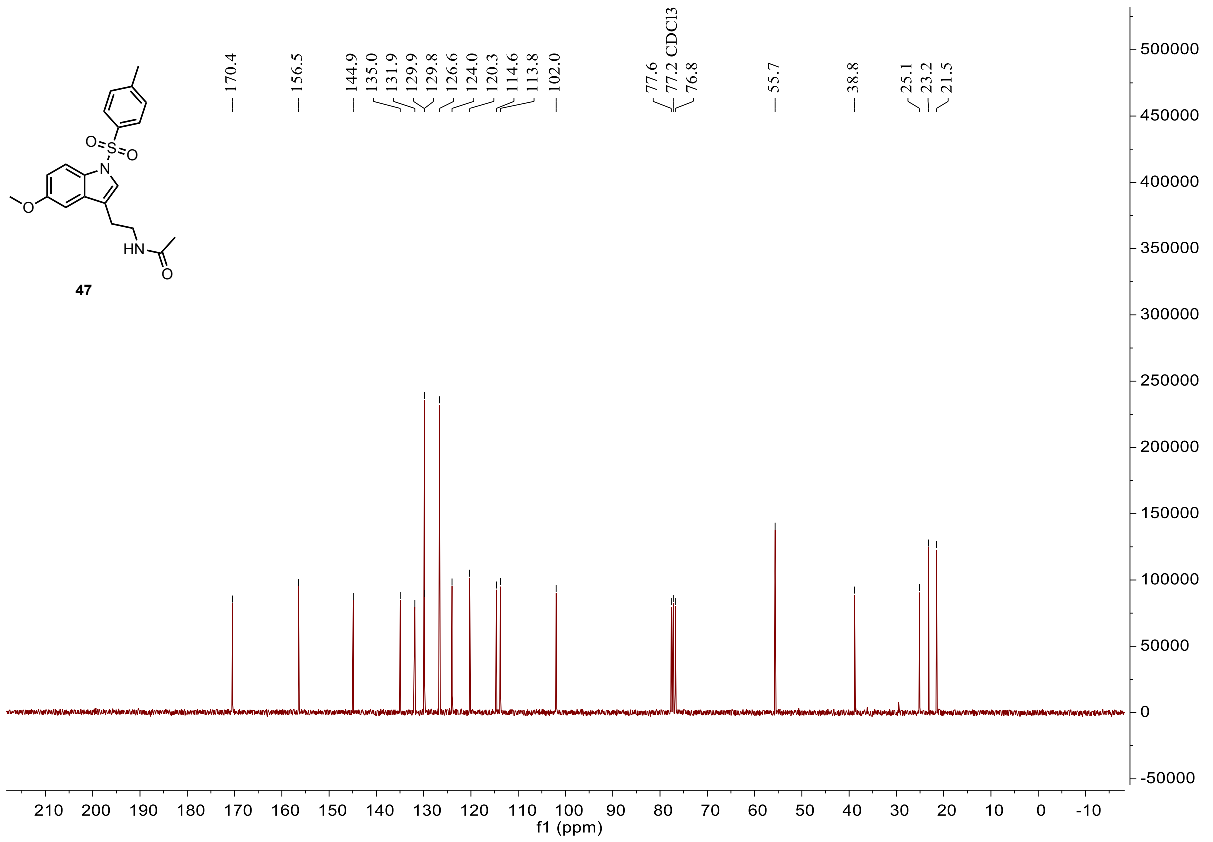

Supplementary Figure 84. ^{13}C-NMR (75 MHz, $CDCl_3$) spectrum of **47**

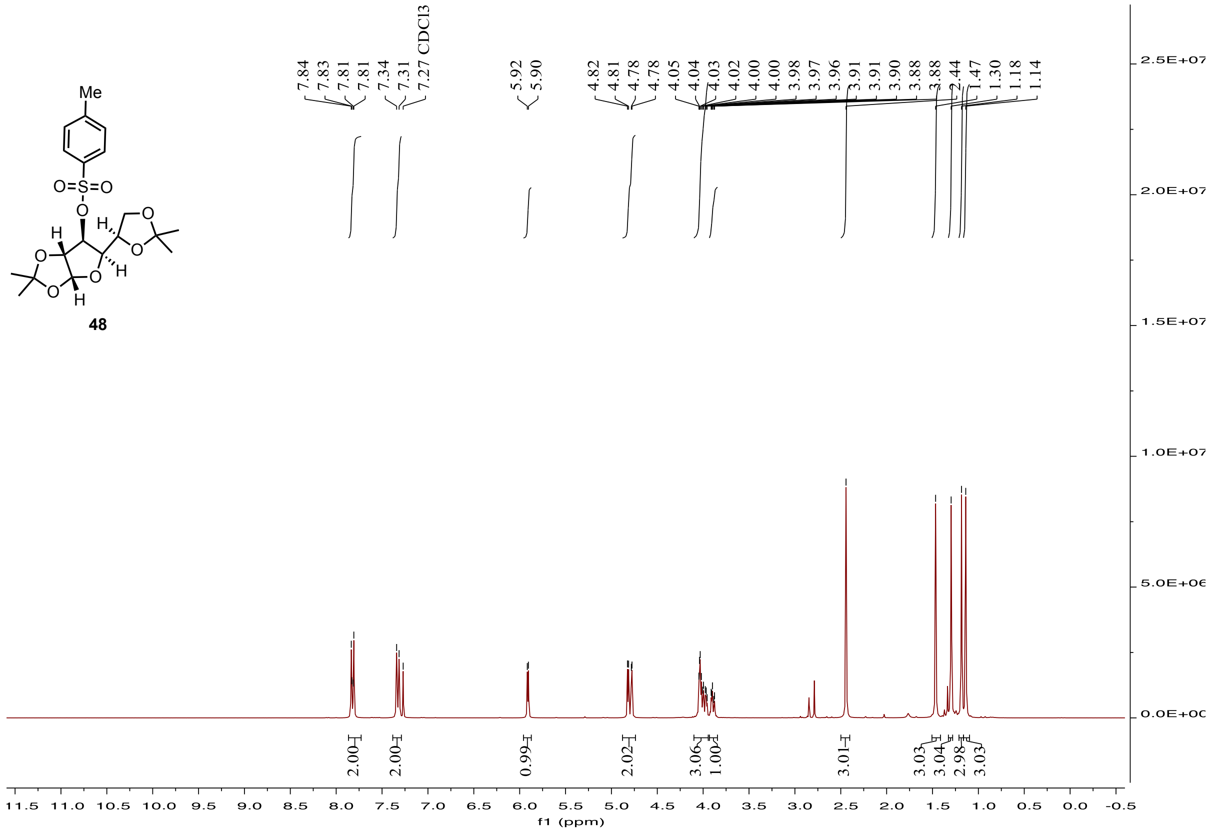

Supplementary Figure 85. ^{1}H-NMR (300 MHz, $CDCl_3$) spectrum of **48**

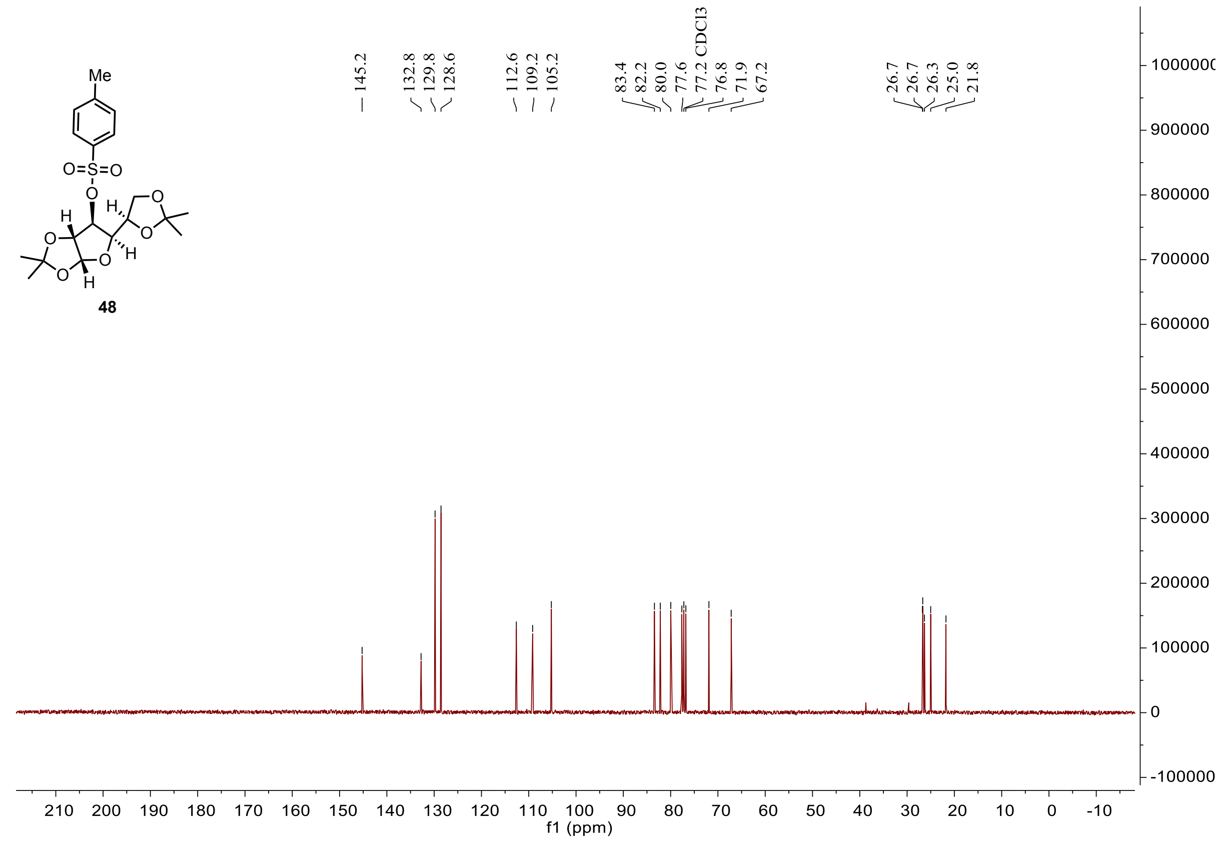

Supplementary Figure 86. ^{13}C-NMR (75 MHz, $CDCl_3$) spectrum of **48**

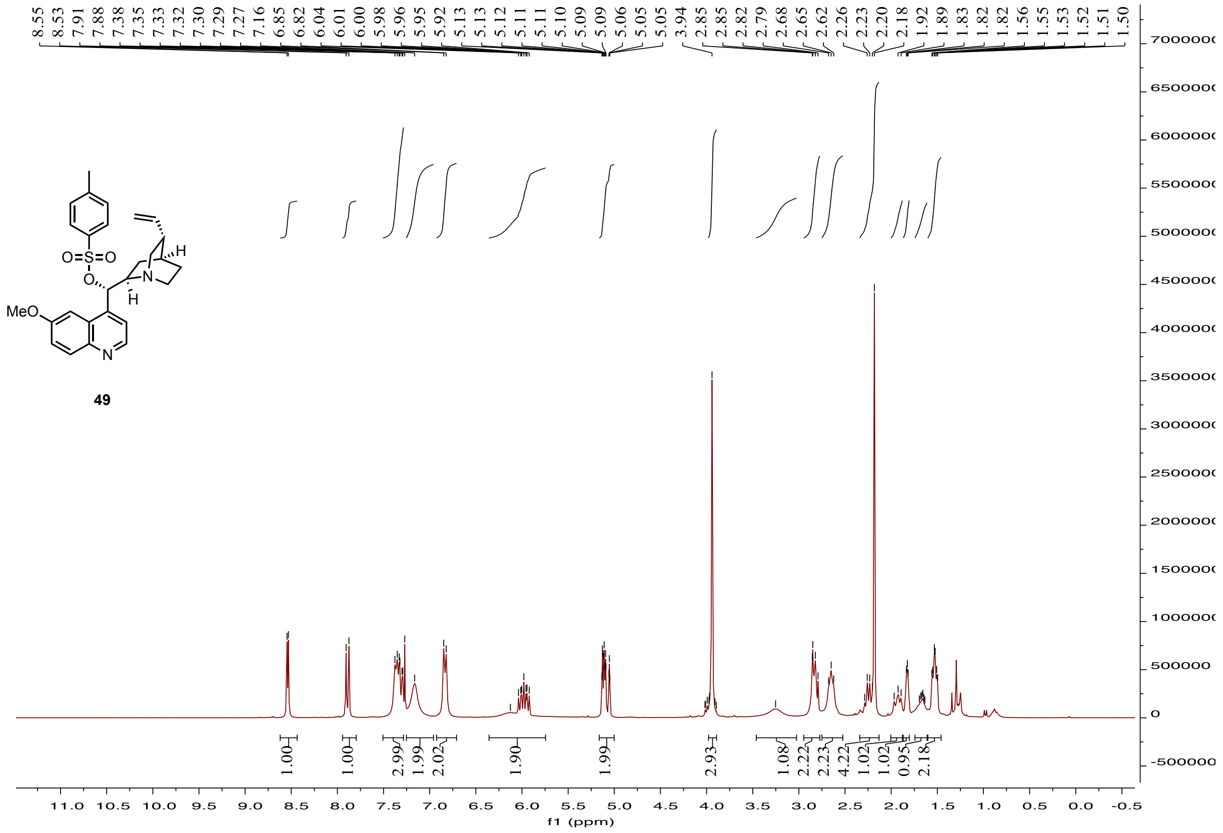

Supplementary Figure 87. ^{1}H-NMR (300 MHz, $CDCl_3$) spectrum of **49**

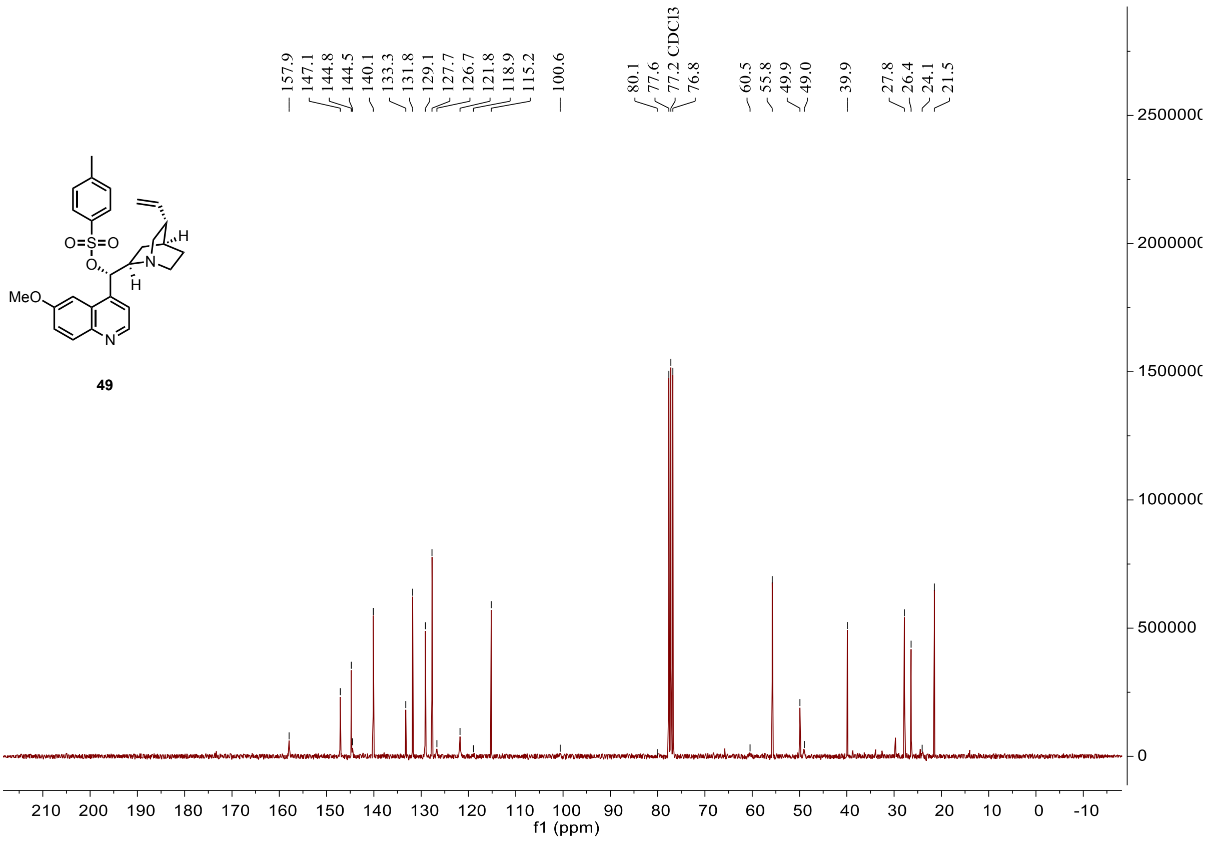

Supplementary Figure 88. ^{13}C-NMR (75 MHz, $CDCl_3$) spectrum of **49**

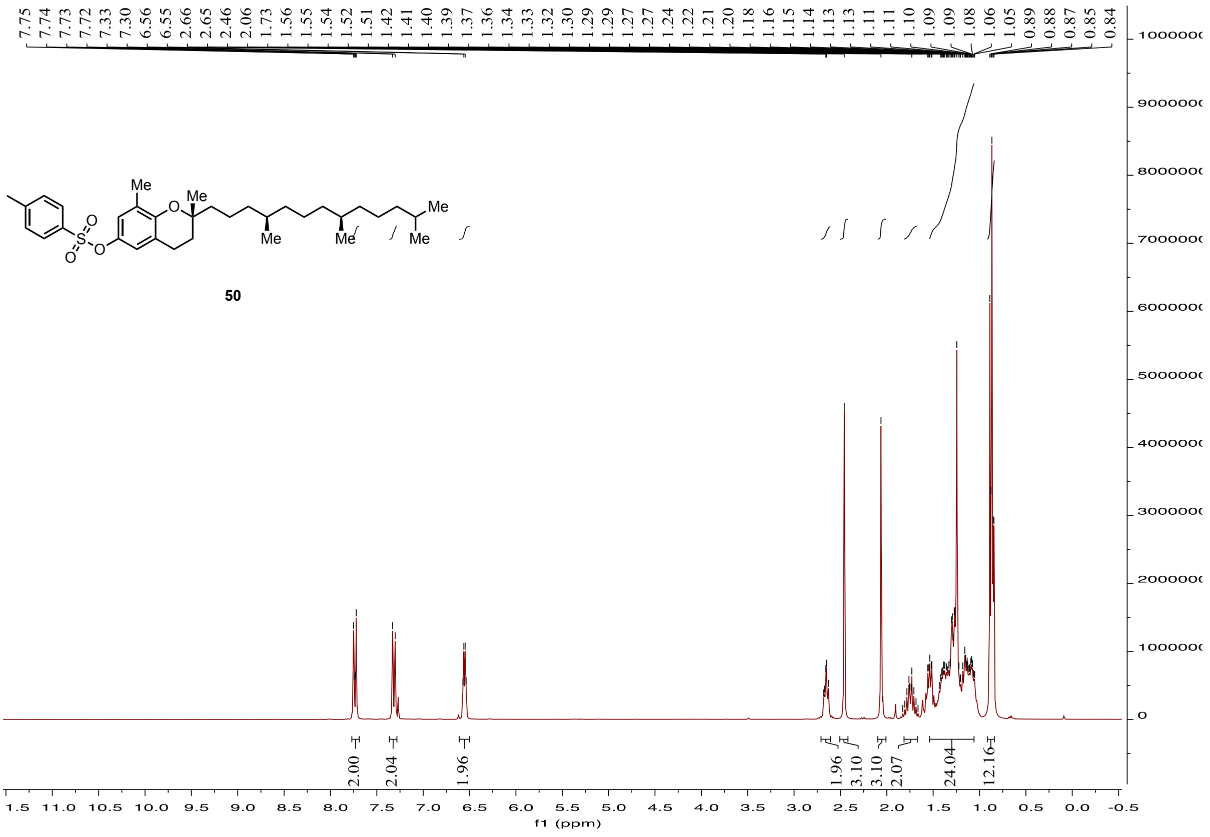

Supplementary Figure 89. ^{1}H-NMR (300 MHz, $CDCl_3$) spectrum of **50**

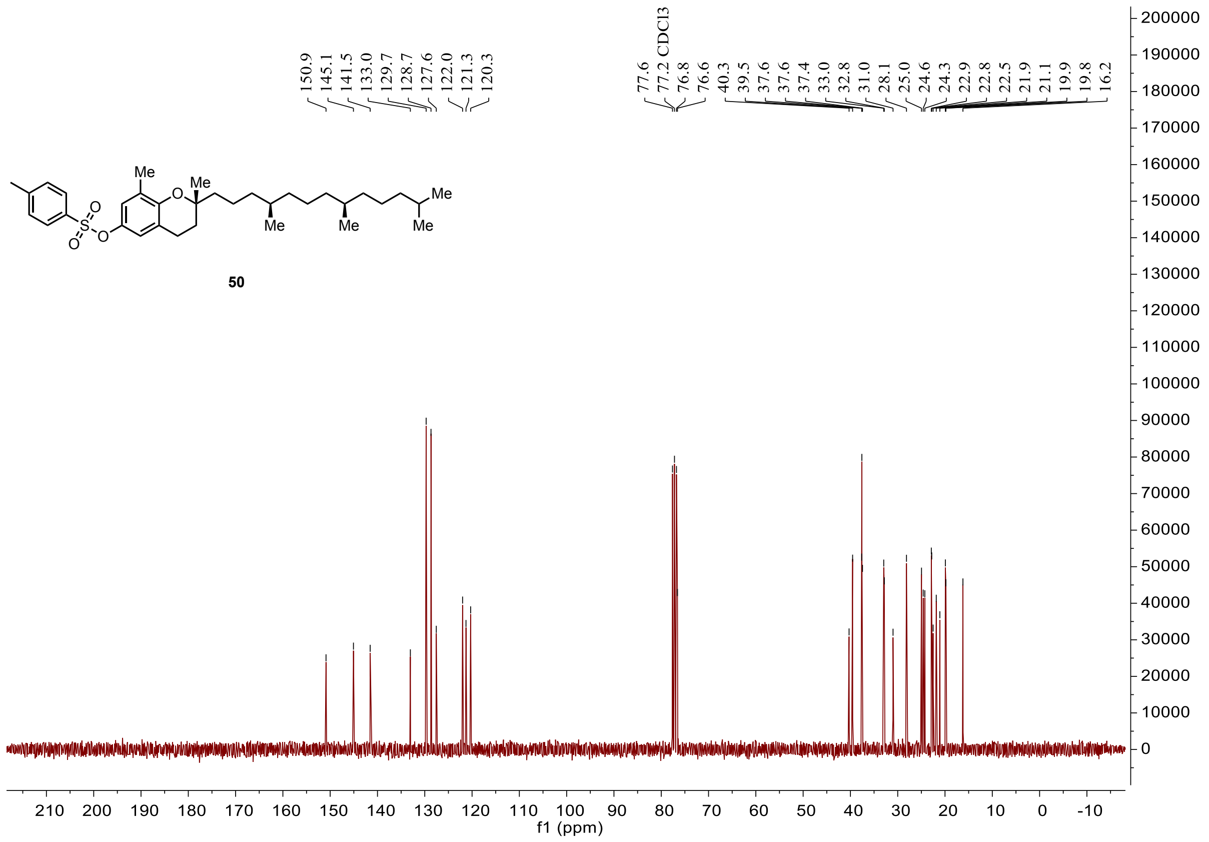

Supplementary Figure 90. ^{13}C-NMR (75 MHz, $CDCl_3$) spectrum of **50**

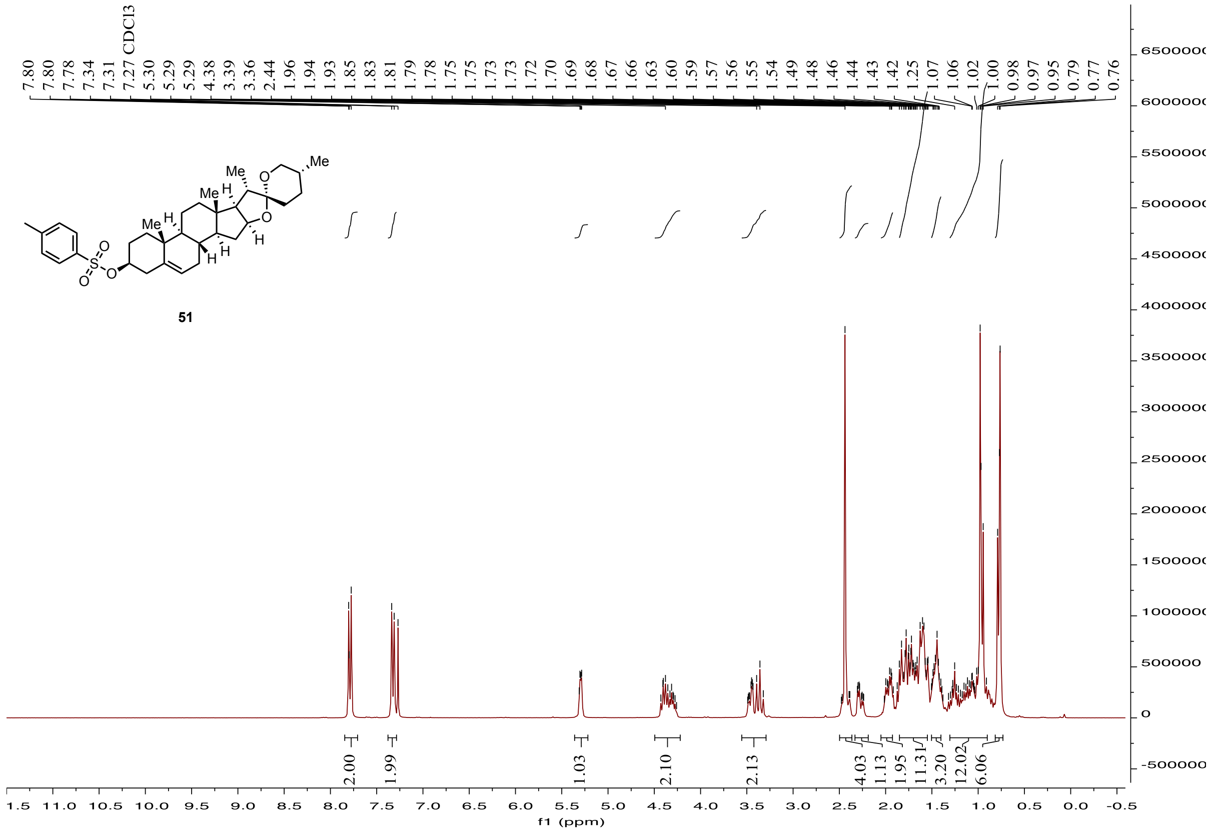

Supplementary Figure 91. ^{1}H-NMR (300 MHz, $CDCl_3$) spectrum of **51**

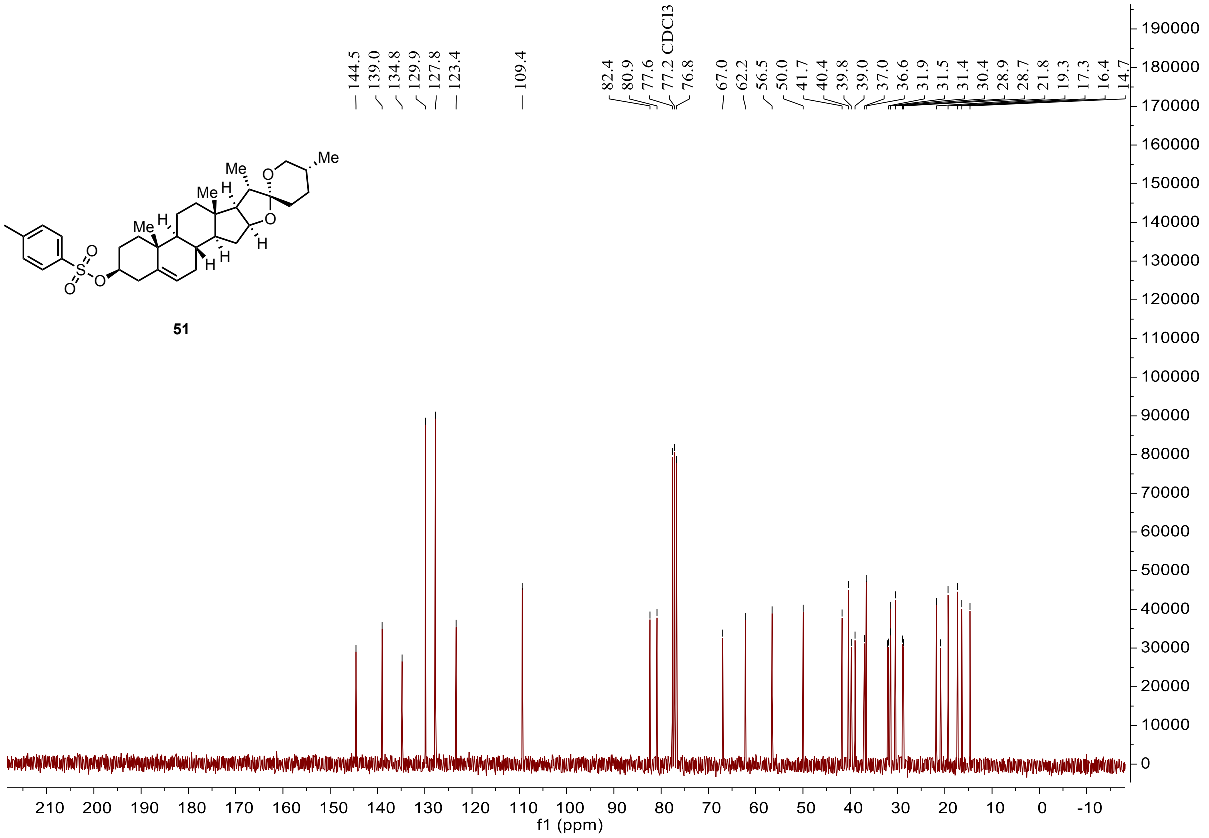

Supplementary Figure 92. ^{13}C-NMR (75 MHz, $CDCl_3$) spectrum of **51**

7 References

1. Matesic, L., Wyatt, N.A., Fraser, B.H., Roberts, M.P., Pham, T.Q., and Greguric, I. Ascertaining the suitability of aryl sulfonyl fluorides for [18F] radiochemistry applications: a systematic investigation using microfluidics. *J. Org. Chem.* **78**, 11262-11270 (2013).
2. Liu, Y., Yu, D., Guo, Y., Xiao, J.C., Chen, Q.Y., and Liu, C. Arenesulfonyl Fluoride Synthesis via Copper-Catalyzed Fluorosulfonylation of Arenediazonium Salts. *Org. Lett.* **22**, 2281-2286 (2020).
3. Mukherjee, H., Debreczeni, J., Breed, J., Tentarelli, S., Aquila, B., Dowling, J.E., Whitty, A., and Grimster, N.P. A study of the reactivity of S((VI))-F containing warheads with nucleophilic amino-acid side chains under physiological conditions. *Org. Biomol. Chem.* **15**, 9685-9695 (2017).
4. Jiang, Y., Alharbi, N.S., Sun, B., and Qin, H.L. Facile one-pot synthesis of sulfonyl fluorides from sulfonates or sulfonic acids. *RSC Adv.* **9**, 13863-13867 (2019).
5. Nielsen, M.K., Ugaz, C.R., Li, W., and Doyle, A.G. PyFluor: A Low-Cost, Stable, and Selective Deoxyfluorination Reagent. *J. Am. Chem. Soc.* **137**, 9571-9574 (2015).
6. Švec, P., Eisner, A., Kolářová, L., Weidlich, T., Pejchal, V., and Růžička, A. Use of C,N-chelated di-n-butyltin(IV) fluoride for the synthesis of acyl fluorides, fluoroformates and fluorophosgene. *Tetrahedron Lett.* **49**, 6320-6323 (2008).
